# Phylogeny of *Verrucosporaceae* (*Basidiomycota*, *Agaricales*): taxonomic novelties in *Cystolepiota* and *Lepiota* from China

**DOI:** 10.3897/imafungus.17.207486

**Published:** 2026-10-09

**Authors:** Xian-Yan Zhou, Tolgor Bau

**Affiliations:** 1 College of Mycology, Jilin Agricultural University, Changchun 130118, China College of Mycology, Jilin Agricultural University Changchun China; 2 Key Laboratory of Edible Fungal Resources and Utilization (North), Ministry of Agriculture and Rural Affairs, Jilin Agricultural University, Changchun 130118, China Key Laboratory of Edible Fungal Resources and Utilization (North), Ministry of Agriculture and Rural Affairs, Jilin Agricultural University Changchun China

**Keywords:** Cryptic diversity, *Lepiota* sect. *Eriophorae* subsect. *Pseudocystolepiotae*, lepiotaceous fungi, morphology, systematics

## Abstract

The family *Verrucosporaceae*, a recently resurrected lepiotoid fungal lineage, has historically been subject to unstable generic delimitations and polyphyletic infrageneric classifications. To resolve these taxonomic ambiguities, we conducted comprehensive morphological examinations and multi-locus phylogenetic analyses (nrITS, nrLSU, *rpb2*, and *tef1-α*) based on extensive specimen sampling across China. Our results strongly support the monophyly of *Verrucosporaceae* and clarify the evolutionary boundaries among its core genera, including *Cystolepiota*, *Echinoderma*, *Lepiota*, *Pulverolepiota*, and *Smithiomyces*. Within *Lepiota*, seven formally defined sections and four unplaced distinct clades were recovered. A novel subsection, *Lepiota* sect. *Eriophorae* subsect. *Pseudocystolepiotae*, is proposed to accommodate an evolutionary transitional lineage between *Cystolepiota* and *Lepiota* sect. *Eriophorae*. Based on an integrative taxonomic approach, we describe 17 species new to science, introduce three new combinations, and reinstate *Lepiota squamulosa*. Furthermore, micromorphological observations indicate that the microstructure of the pileus and stipe coverings shares structural homology, reinforcing its reliability as a criterion for infrageneric delimitation. Ultimately, this study expands the current understanding of cryptic diversity within *Verrucosporaceae* in China and provides useful morphological and phylogenetic data for future evolutionary studies on lepiotaceous fungi.

## Introduction

*Verrucosporaceae* is a historically proposed but long-neglected family-level taxon that has recently been resurrected and redefined from *Agaricaceaesensu lato* (s.l.) (Kalichman et al. 2020). This family primarily comprises saprotrophic fungi characterized by “lepiotoid” morphological features, which play an important role in litter decomposition and nutrient cycling within forest ecosystems (Yang et al. 2019). Their typical morphological characteristics include: a pale pileus surface often covered with brightly colored squamules; free lamellae that are usually white to cream, and rarely yellow, vinaceous, or blue-green; a central stipe with a distinct annulus or annular zone; hyaline, smooth or ornamented basidiospores lacking a germ pore, which vary in shape from ellipsoid and cylindrical to distinctly penguin-shaped or spurred; the frequent presence of cheilocystidia alongside a general absence of pleurocystidia; a pileus covering structured as a trichoderm, cutis, or conioderm; and the typical presence of clamp connections (Yang et al. 2019; Li et al. 2025).

The taxonomic history of this group is intricate, revolving primarily around its oldest and most species-rich genus, *Lepiota* (Pers.) Gray. *Lepiota* was first introduced under the genus *Agaricus* L. by Persoon (1797) and later elevated to generic rank by Gray (1821). Fries (1821) restricted the genus to terrestrial, white-spored taxa. Since then, the generic circumscription has been continuously revised by subsequent mycologists, including Patouillard (1888), Fayod (1889), Morgan (1906), and Lange (1915). Kühner (1936) further established 14 sections within *Lepiota* based on micromorphological features—such as the squamule structures on the pileus and stipe, and the size and shape of basidiospores—laying the micromorphological foundation for modern infrageneric classification. Singer (1943, 1944, 1948) continually revised *Lepiota* and gradually segregated several independent genera: *Smithiomyces* Singer (Singer 1944) was erected for taxa with a smooth pileus and an extremely thin veil composed of hyphae and sphaerocysts, while *Cystolepiota* Singer (Singer 1951) was established based on a pileus covering composed of sphaerocysts and the possession of non-dextrinoid basidiospores. In his final taxonomic system, Singer (1986) consolidated *Lepiota* into eight classical sections.

During the 20^th^ century, other members of this family were successively described. Earle (1909) validly published *Chamaemyces* Battarra ex Earle. Velenovský (1921) erected *Melanophyllum* for species characterized by basidiomata that blacken upon aging and basidiospores with yellowish tints. Horak (1967) elevated *Lepiota verrucispora* Beeli, noted for its distinctly verrucose basidiospores, to the independent genus *Verrucospora* E. Horak. The generic boundaries of these taxa and their relationships with *Lepiota* remained highly controversial. Knudsen (1978, 1980) initially transferred *Lepiota* sect. *Echinatae* to *Cystolepiota*, but later returned it to *Lepiota*. Bon (1981, 1991) subsequently reassigned it to *Cystolepiota* under the subgenus *Echinoderma*, which he later elevated to generic rank. Bon (1993) also erected *Pulverolepiota* Bon to accommodate species lacking clamp connections and possessing unique pileus squamules composed of interwoven, frequently lobed, or occasionally branched cells. Despite extensive systematic efforts during this period, a stable consensus on morphology-based generic delimitations was not achieved, leading Vellinga and Huijser (1998) to tentatively reduce *Pulverolepiota* to sectional rank within *Cystolepiota*.

The advent of molecular phylogenetics provided a reliable tool to resolve these taxonomic ambiguities. Johnson and Vilgalys (1998) first demonstrated that several morphology-defined genera were not monophyletic. Based on nrITS and nrLSU sequence data, Vellinga (2003) showed that *Lepiota* sect. *Ovisporae* was polyphyletic, whereas *Cystolepiota*, *Lepiota* sect. *Echinatae*, *Melanophyllum*, and *Pulverolepiota* formed a single monophyletic clade. Within this clade, however, molecular evidence revealed that *Pulverolepiota* was phylogenetically distinct from the core *Cystolepiota* group, thereby providing support for maintaining *Pulverolepiota* as an independent genus. Notably, Vellinga (2003) also identified two subclades within *Lepiota* sect. *Echinatae* distinguished by large and small spores, respectively, laying the groundwork for future taxonomic restructuring. Vellinga (2004) further confirmed the monophyly of *Cystolepiota*, *Melanophyllum*, *Echinoderma*, and *Pulverolepiota*. Subsequent studies utilizing broader taxon sampling (Liang 2007, 2016; Sysouphanthong et al. 2011, 2020b; Hou and Ge 2020) have consistently corroborated the non-monophyletic nature of these traditional sections.

At the family level, Roze (1876) previously erected *Lepiotaceae* to accommodate these taxa, but the name was invalidly published. Jülich (1982) proposed *Verrucosporaceae* with *Verrucospora* as the type genus, a concept rarely adopted until Kalichman et al. (2020) formally proposed subsuming the lepiotoid fungi under this family [replacing the synonymous *Lepiotaceae* (Yang et al. 2024)], encompassing nine genera: *Chamaemyces*, *Cystolepiota*, *Echinoderma*, *Lepiota*, *Melanophyllum*, *Neosecotium* Singer & A.H. Sm., *Pulverolepiota*, *Smithiomyces*, and *Verrucospora*. Nevertheless, subsequent molecular phylogenetic investigations led to significant adjustments in generic placements: *Chamaemyces fracidus* (Fr.) Donk, the type species of *Chamaemyces*, along with members of *Verrucospora*, have been shown to nest within *Lepiota* (Li et al. 2025; Li et al. 2026). Consequently, *Verrucosporaceae* currently comprises eight valid genera: *Cystolepiota*, *Echinoderma*, *Lepiota*, *Melanophyllum*, *Neosecotium*, *Pulverolepiota*, *Verrucospora*, and *Smithiomyces*, although *Neosecotium* currently lacks supporting molecular data (Li et al. 2025).

With the family-level framework stabilizing, recent research has shifted toward precise generic delimitation and infrageneric classification. Hou and Ge (2020) demonstrated that although some *Lepiota* species are macro-morphologically similar to *Echinoderma*, they belong to independent lineages differentiable by basidiospore size. Qu et al. (2023) resurrected the independent status of *Pulverolepiota* and observed that *Cystolepiota* and *Melanophyllum* clustered into a major clade, prompting Zhou and Bau (2024) to propose the concept of *Cystolepiotasensu lato* (s.l.). Focusing on *Lepiota*, Sarawi et al. (2025a) conducted a multi-locus reassessment that reaffirmed the monophyly of *Echinoderma*. This study also addressed the polyphyly of the traditional *Lepiota* sect. *Echinatae* s.l., reallocating species into the new *Lepiota* sect. *Eriophorae* and identifying others as a basal lineage of *Lepiota* sect. *Lepiota*. Sarawi et al. (2025a) further highlighted that pileus covering microstructure exhibits high evolutionary conservatism, making it a reliable criterion for sectional delimitation, whereas basidiospore shape demonstrates considerable convergence.

Although the generic and sectional framework of *Verrucosporaceae* is becoming clearer, comprehensive documentation of its species diversity remains challenging. The core genus *Lepiota*, in particular, exhibits high morphological convergence with allied taxa like *Echinoderma*, leading to persistent taxonomic confusion that necessitates broader regional data. Furthermore, in the ecologically diverse regions of China, the species richness within this family remains largely under-explored. Therefore, based on extensive specimen sampling across China and supported by a family-level phylogenetic reconstruction, this study focuses on the taxonomic revision of major lineages within *Verrucosporaceae*. By integrating multi-locus molecular data with detailed micromorphological analyses, we describe 17 species new to science, propose a new subsection within *Lepiota*, and provide supplementary morphological and phylogenetic observations on the *Melanophyllum haematospermum* complex. These findings contribute to a more natural classification and a clearer understanding of the evolutionary boundaries among these historically contentious groups.

## Materials and methods

### Sampling and morphological analyses

Approximately 950 specimens, collected from China between 1996 and September 2025, were examined in this study. Macroscopic morphological characteristics, habitat information, and field photographs were documented during field trips. Color terms follow Kornerup and Wanscher (1967), and morphological descriptions follow Vellinga (1988). Specimen processing, micro-morphological observation and measurement, and scanning electron microscopy (SEM) of basidiospores were conducted following previously described methods Zhou and Bau (2024).

### DNA extraction, PCR amplification, and sequencing

Total genomic DNA was extracted from fresh or dried specimens using the NuClean PlantGen DNA Kit (CWBIO, Beijing, China). Four loci were amplified by PCR: the nuclear ribosomal internal transcribed spacer (nrITS), the nuclear ribosomal large subunit (nrLSU), the RNA polymerase II second largest subunit (*rpb2*), and the translation elongation factor 1-alpha (*tef1-α*). The primer pairs used were ITS1F/ITS4 (White et al. 1990; Gardes and Bruns 1993), LR0R/LR5 (Vilgalys and Hester 1990), RPB2-6F/RPB2-7.1R (Liu et al. 1999), and EF1-983F/EF1-1567R (Rehner and Buckley 2005), respectively. PCR protocols and thermocycling conditions followed previous studies (Zhou and Bau 2024). The PCR products were sequenced by Sangon Biotech (Shanghai, China). All newly generated sequences have been deposited in GenBank (Suppl. material 1).

### Phylogenetic studies

To evaluate the phylogenetic relationships within the family *Verrucosporaceae*, phylogenetic trees were reconstructed based on a concatenated dataset of nrITS, nrLSU, *rpb2*, and *tef1-α* sequences. The analysis included representative species from the genera *Cystolepiota*, *Echinoderma*, *Lepiota*, *Melanophyllum*, *Pulverolepiota*, *Smithiomyces*, and *Verrucispora*, with *Coprinus* species selected as the outgroup based on previous studies (Zhou and Bau 2024; Li et al. 2025). The final dataset consisted of 467 nrITS, 167 nrLSU, 100 *rpb2*, and 85 *tef1-α* sequences, among which 353 sequences were newly generated in this study (158 nrITS, 85 nrLSU, 55 *rpb2*, and 55 *tef1-α*), and the remaining sequences were retrieved from GenBank (Suppl. material 1).

Sequences for each locus were aligned independently using MAFFT v.7.110 (Katoh et al. 2019), followed by manual inspection and optimization in MEGA v.7.0.26 (Kumar et al. 2016). Unreliable aligned regions and gaps were removed using the “-automated1” command in trimAl (Capella-Gutiérrez et al. 2009). The aligned sequences were then concatenated using the “Concatenate Sequence” function in PhyloSuite v.2 (Zhao et al. 2025), yielding a combined alignment of 2731 base pairs (bp). The sequence partitions were defined as follows: nrITS (1–671 bp), nrLSU (672–1493 bp), *rpb2* (1494–2152 bp), and *tef1-α* (2153–2731 bp). Gaps were treated as missing data.

Phylogenetic reconstructions were performed using Maximum Likelihood (ML) and Bayesian Inference (BI). To account for evolutionary heterogeneity across different gene regions, a partitioned analysis was applied to the concatenated dataset. For the ML analysis, ModelFinder v.3.0.1 (Kalyaanamoorthy et al. 2017) was employed to select the best-fit nucleotide substitution models under the Akaike Information Criterion (AIC). The ML tree was then inferred using IQ-TREE v.3.0.1 (Nguyen et al. 2015) with an edge-linked partition model. The selected best-fit models were TVM+F+R8 for nrITS, GTR+R4 for rpb2, and GTR+F+I+R4 for both nrLSU and *tef1-α*. Node support was evaluated using 1,000 ultrafast bootstrap (Minh et al. 2013) replicates and the Shimodaira–Hasegawa approximate likelihood-ratio test (SH-aLRT) (Guindon et al. 2010).

For the BI analysis, optimal partition models were determined using ModelFinder v.3.0.1 (Kalyaanamoorthy et al. 2017) based on the Bayesian Information Criterion (BIC), yielding GTR+F+I+G4 for nrITS and nrLSU, GTR+G4 for *rpb2*, and GTR+I+G4 for *tef1-α*. The BI analysis was executed in MrBayes v.3.2.7a (Ronquist et al. 2012), running two independent runs for 60,000,000 generations. The runs were set to terminate once the average standard deviation of split frequencies (ASDSF) dropped below 0.01. The initial 25% of the sampled trees were discarded as burn-in, and the effective sample size (ESS) values for all parameters were verified to be well above 200. The resulting phylogenetic trees were visualized and annotated using FigTree v.1.4.3 (Rambaut 2016) and tvBOT v.2.6.1 (Xie et al. 2023).

## Results

### Phylogenetic analyses

**Figure 1.**
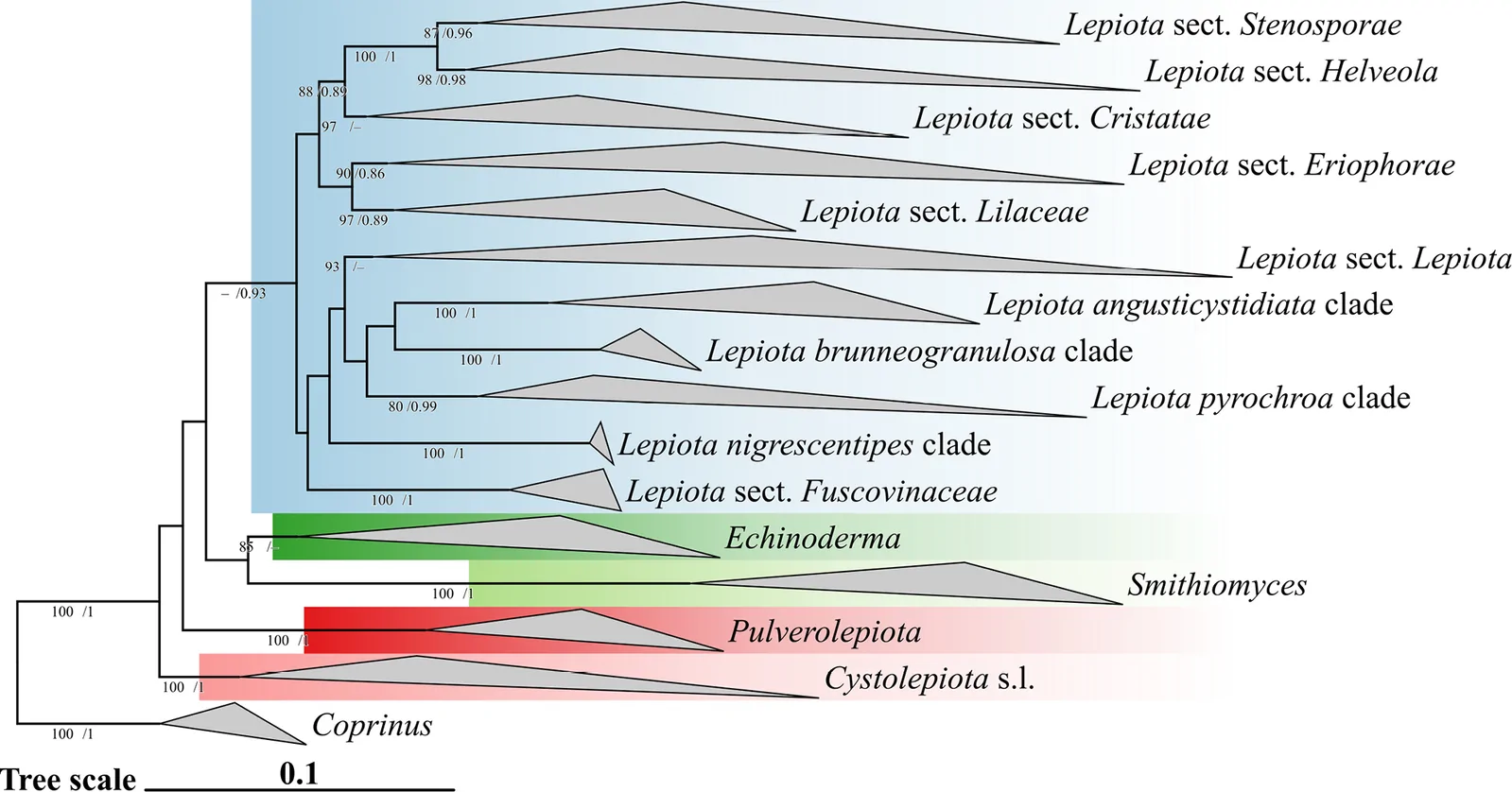
Phylogenetic overview of *Verrucosporaceae* inferred from a concatenated dataset of nrITS, nrLSU, *rpb2*, and *tef1-α* sequences. The tree was constructed using Bayesian Inference (BI). Collapsed branches represent sectional or generic lineages. Maximum Likelihood ultrafast bootstrap support values (UFBoot ≥ 80%) and Bayesian posterior probabilities (PP ≥ 0.85) are indicated at the nodes (UFBoot/PP).

**Figure 2.**
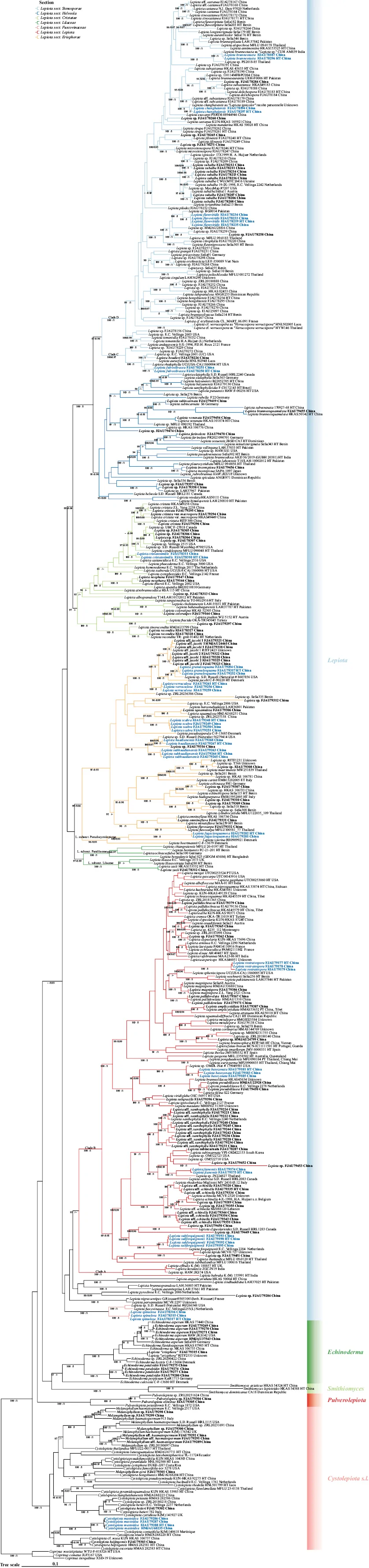
Detailed phylogenetic tree of *Verrucosporaceae* inferred from a concatenated dataset of nrITS, nrLSU, *rpb2*, and *tef1-α* sequences. The tree was constructed using Bayesian Inference (BI). Maximum Likelihood ultrafast bootstrap support values (UFBoot ≥ 80%) and Bayesian posterior probabilities (PP ≥ 0.85) are indicated at the nodes (UFBoot/PP). Newly described species are highlighted in bold blue, and newly generated sequences are marked in bold black.

**Figure 3.**
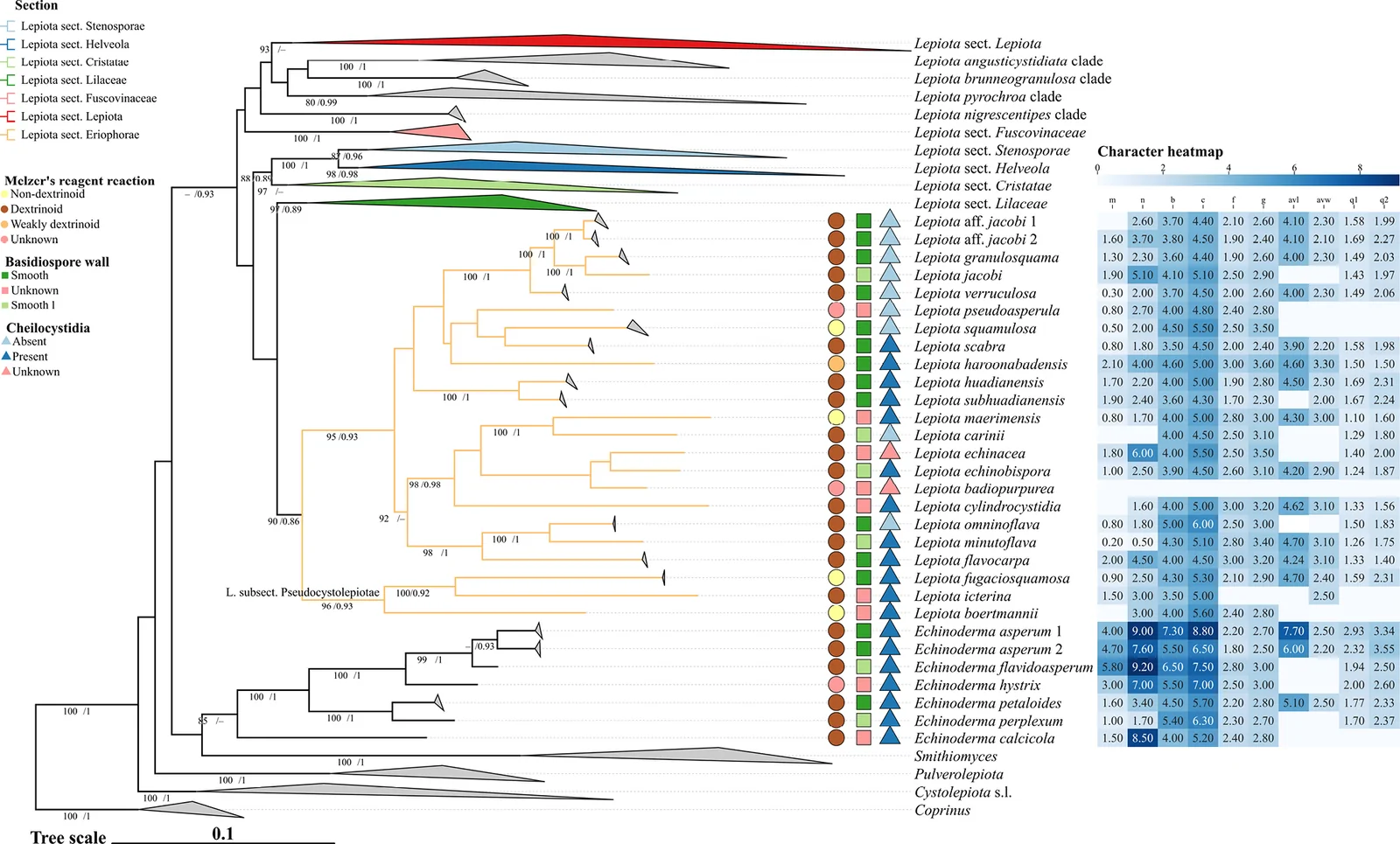
Detailed phylogenetic tree of *Verrucosporaceae* inferred from a concatenated dataset of nrITS, nrLSU, *rpb2*, and *tef1-α* sequences. The tree was constructed using Bayesian Inference (BI). This tree provides species-level resolution for *Echinoderma*, *Lepiota* sect. *Pseudocystolepiotae*, and *Lepiota* sect. *Eriophorae*, with all other lineages collapsed for clarity. Maximum Likelihood ultrafast bootstrap support values (UFBoot ≥ 80%) and Bayesian posterior probabilities (PP ≥ 0.85) are indicated at the nodes (UFBoot/PP). Selected diagnostic micro-morphological characters are mapped onto the topology. In the character heatmap, m–n denotes the range of pileus diameter (cm); b–c × f–g represents the range of basidiospore dimensions; and q1–q2 denotes the range of the Q value (length/width ratio). In the basidiospore wall column, “smooth” indicates smooth walls confirmed by SEM, while “smooth l” denotes smooth walls reported in literature.

The phylogenetic relationships of *Verrucosporaceae* were reconstructed based on a concatenated dataset of nrITS, nrLSU, *rpb2*, and *tef1-α* sequences. Because the topologies generated by Maximum Likelihood (ML) and Bayesian Inference (BI) were largely congruent without significant conflicts at major nodes, only the BI phylogenetic tree is presented (Figs 1, 2, 3), with branch support values exceeding the defined thresholds (UFBoot > 80% / PP > 0.85) indicated at the respective nodes. In the resulting trees, newly described species are highlighted in bold blue, and newly generated sequences in bold black. Rooted with *Coprinus* as the outgroup, the genera *Lepiota*, *Smithiomyces*, *Echinoderma*, *Cystolepiota* s.l., and *Pulverolepiota* were all resolved as monophyletic groups (Fig. 1).

*Cystolepiota* s.l. is positioned at the base of *Verrucosporaceae*, forming a strongly supported monophyletic clade (100/1) comprising *Cystolepiota* and *Melanophyllum* (Figs 1, 2). The principal morphological synapomorphy of this clade is a pileus covering composed of loosely arranged sphaerocysts, coupled with the presence of clamp connections (Vellinga 2001a). However, the lamellae of *Melanophyllum* typically exhibit vinaceous, or blue-green hues that become blackish upon drying, whereas those of *Cystolepiota* are generally white and either discolor or remain unchanged upon drying (Vellinga 2001a, 2001d; Qu et al. 2023; Zhou and Bau 2024). A novel species described herein, *Cystolepiota mastoidea*, nests within this clade. It can be readily distinguished from other *Cystolepiota* species by its discoloring basidiomata and distinctive clavate cheilocystidia with a capitate apex.

The *Pulverolepiota* clade solely encompasses species of this genus (Figs 1, 2), forming a strongly supported monophyletic group (100/1). Macroscopically, it closely resembles members of *Cystolepiota*; however, the pileal squamules of *Pulverolepiota* are composed of elongated and inflated cells. Furthermore, its basidiospores exhibit a slow dextrinoid reaction in Melzer’s reagent, and clamp connections are absent (Vellinga and Huijser 1998; Qu et al. 2023).

Species of *Smithiomyces* similarly cluster into a well-supported clade (100/1) (Figs 1, 2). The diagnostic features of this genus include a heteromerous fragmentary veil on the surface of the pileus and stipe, ornamented basidiospores, and the presence of clamp connections (Ge et al. 2021).

The genus *Echinoderma* was recovered as monophyletic in the present study (Figs 1, 2, 3), albeit with moderate statistical support (85/–). Currently, this genus accommodates six recognized species. With the exception of the basally diverging *E. calcicole* (Knudsen) Bon, the remaining five taxa form a strongly supported core clade (100/1). Morphologically, the primary diagnostic feature of this genus is the presence of verrucose to echinate squamules on the pileus surface, which are microscopically composed of globose cells arranged in chains (Sarawi et al. 2025a).

Within *Lepiota* (–/0.93), the phylogenetic reconstruction identified 11 major clades (Figs 1, 2), seven of which correspond to formally defined sections: *L.* sect. *Stenosporae*, *L.* sect. *Helveolae*, *L.* sect. *Cristatae*, *L.* sect. *Lilaceae*, *L.* sect. *Fuscovinaceae*, *L.* sect. *Lepiota*, and *L.* sect. *Eriophorae* (including the newly established subsect. *Pseudocystolepiotae*), The remaining four represent currently unplaced lineages, namely the *L. angusticystidiata* clade, *L. brunneogranulosa* clade, *L. pyrochroa* clade, and *L. nigrescentipes* clade.

*Lepiota* sect. *Fuscovinaceae* occupies the most basal position within the genus (Figs 1, 2). Macroscopically, it resembles species of *L.* sect. *Eriophorae* and *Echinoderma*, featuring echinate to repent-villose squamules; however, its pileus covering is organized as a trichoderm, and it lacks clamp connections (Vellinga 2001b; Sarawi et al. 2025a). This section currently contains only two species: *Lepiota fuscovinacea* and the newly discovered *L. spinulosa*. The latter primarily differs from the closely related *L. fuscovinacea* by lacking vinaceous pileus tones and possessing significantly larger basidiospores.

*Lepiota* sect. *Lepiota* was recovered as monophyletic but lacked strong support (93/–) (Figs 1, 2). Species within this section further diverged into two clades. Clade A (100/1) contains the majority of *L.* sect. *Lepiota* species, including four new species described in this study. In contrast, the basal Clade B (100/1) comprises three species: *L. efibulis* Knudsen, *L. keralensis* Pious & T.K.A. Kumar, and *L. babruka* T.K.A. Kumar & Manim. Microscopically, the pileal squamules of *L. efibulis* and *L. keralensis* consist of sphaerocysts arranged in chains (Vellinga 2001b; Pious and Arun 2025). In *L. babruka*, located at the base of Clade B, its pileus covering transitions from a repent cutis at the margin to a fasciculate trichoderm centrally, with terminal hyphae appearing as a trichoepithelium (Arun Kumar and Manimohan 2009). The pileus surface characteristics of these three basal species differ markedly from the typical trichoderm structure defining Clade A, which likely contributes to the moderate phylogenetic support for *L.* sect. *Lepiota* as a whole.

*Lepiota* sect. *Lilaceae* forms a monophyletic group that bifurcates into two clades corresponding to established subsections (97/0.89) (Figs 1, 2). *Lepiota* subsect. *Paralilaceae* (100/1), currently containing four species, is characterized by pileal squamules with orange-brown tones, a hymeniform pileus covering, and typically dextrinoid basidiospores (except in *L. chiangraiensis* Sysouph., Thongkl. & K.D. Hyde). The other subsection, *L.* subsect. *Lilaceae* (100/1), encompasses three species characterized by purplish-brown squamules, a similar hymeniform pileus covering, and non-dextrinoid basidiospores (Sarawi et al. 2025a).

*Lepiota* sect. *Eriophorae* was recovered as a broad monophyletic group (Figs 1, 2, 3) with moderate nodal support (90/0.86). Within this expanded section, two distinct sister clades are resolved. The first clade corresponds to the core *L.* sect. *Eriophorae* (95/0.93), which encompasses six newly described species from the present study. Its core diagnostic features include verrucose to conical pileal squamules and a pileus covering predominantly composed of three cell layers. The second clade is herein established as a novel subsection, *L.* sect. *Eriophorae* subsect. *Pseudocystolepiotae* (96/0.93), currently comprising three species. Morphologically, this subsection exhibits a transitional morphology that bridges the two clades. The basal species, *L. boertmannii*, shares the conical pileal squamules and three-layered pileus covering typical of the core *L.* sect. *Eriophorae* (Knudsen 1980). However, the remaining two species in this subsection (*L. icterina* and the newly described *L. fugaciosquamosa*) diverge by possessing powdery squamules and a pileus covering restricted to only two cell layers (Knudsen 1978).

*Lepiota* sect. *Cristatae* forms a monophyletic group (97/–) but lacks Bayesian posterior probability support (Figs 1, 2). Macroscopically, its pileus covering remains intact at the disc but disrupts into small, block-like squamules toward the margin. The pileus covering is composed of hymeniform cells, and the basidiospores are ellipsoid or spurred (Sarawi et al. 2025a). One new species, *Lepiota cristatosimilis*, is described within this section.

*Lepiota* sect. *Helveolae* forms a strongly supported monophyletic clade (98/0.98) (Figs 1, 2), encompassing the majority of *Lepiota* species known to contain amatoxins. Species in this section exhibit variable basidiome coloration, typically possess a distinct annular zone on the stipe, and have a trichodermal pileus covering (Sarawi et al. 2025a).

*Lepiota* sect. *Stenosporae* (Figs 1, 2) was also recovered as a monophyletic group (87/0.96), further dividing into two subclades. Clade D (100/1) possesses a trichodermal pileus covering, and the species within its lineages exhibit significant phylogenetic clustering by coloration (Zhou and Bau 2026). Two new species with orange-brown tones (*Lepiota brunneotacta* and *L. changbaiensis*) and one with yellow tones (*L. flavoviridis*) were discovered in this subclade. Conversely, Clade C (100/0.97) features a pileus covering composed of a cutis, and a new species, *Lepiota fulvoolivacea*, is proposed within this lineage.

### Taxonomy

#### Micromorphology of pileus and stipe coverings

**Figure 4.**
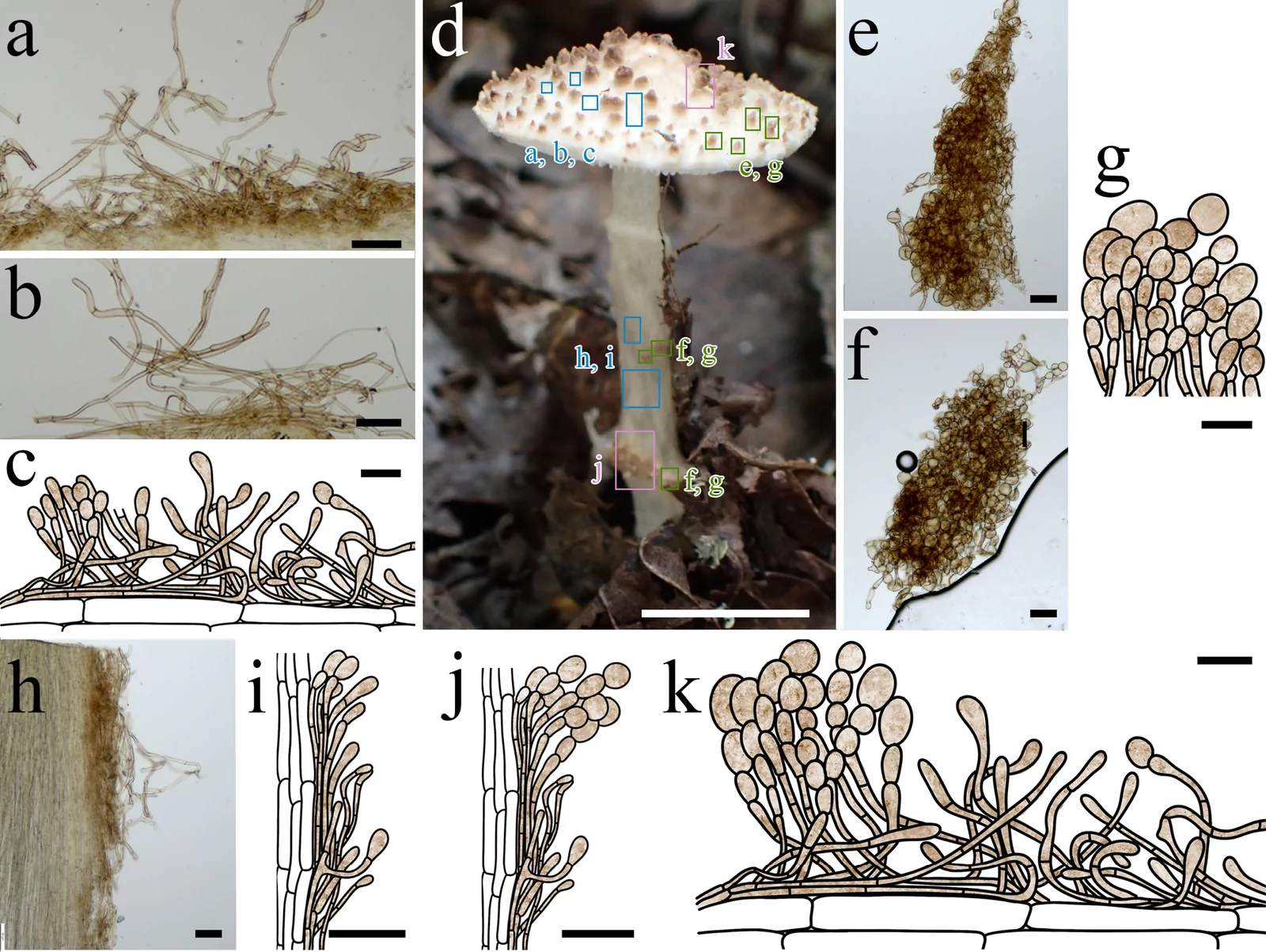
Micromorphology of pileus and stipe coverings in *Echinoderma petaloides*. **a, b**. Fibrous-floccose squamules on pileus surface, composed of elongate hyphae with inflated terminal cells. **c**. Line drawing of the fibrous-floccose pileus squamules. **d**. Basidiomes of *Echinoderma petaloides* (FJAU79274). **e**. Echinulate-verrucose squamules on pileus surface, formed by globose cells arranged in chains. **f**. Echinulate-verrucose squamules on stipe surface, similarly composed of globose chains. **g**. Line drawing of the echinulate-verrucose squamules. **h**. Fibrous-floccose squamules on stipe surface, composed of elongate hyphae with inflated apices. **i**. Line drawing of the fibrous-floccose stipe squamules. **j**. Line drawing of stipe covering: a three-layered structure with a basal layer of broad, hyaline hyphae, an intermediate layer of elongate hyphae (fibrous squamules), and an apical layer of globose chains (echinulate-verrucose squamules). **k**. Line drawing of pileus covering, showing the same three-layered architecture. Scale bars: 1 cm (**d**); 50 μm (**a–c, e–k**).

**Figure 5.**
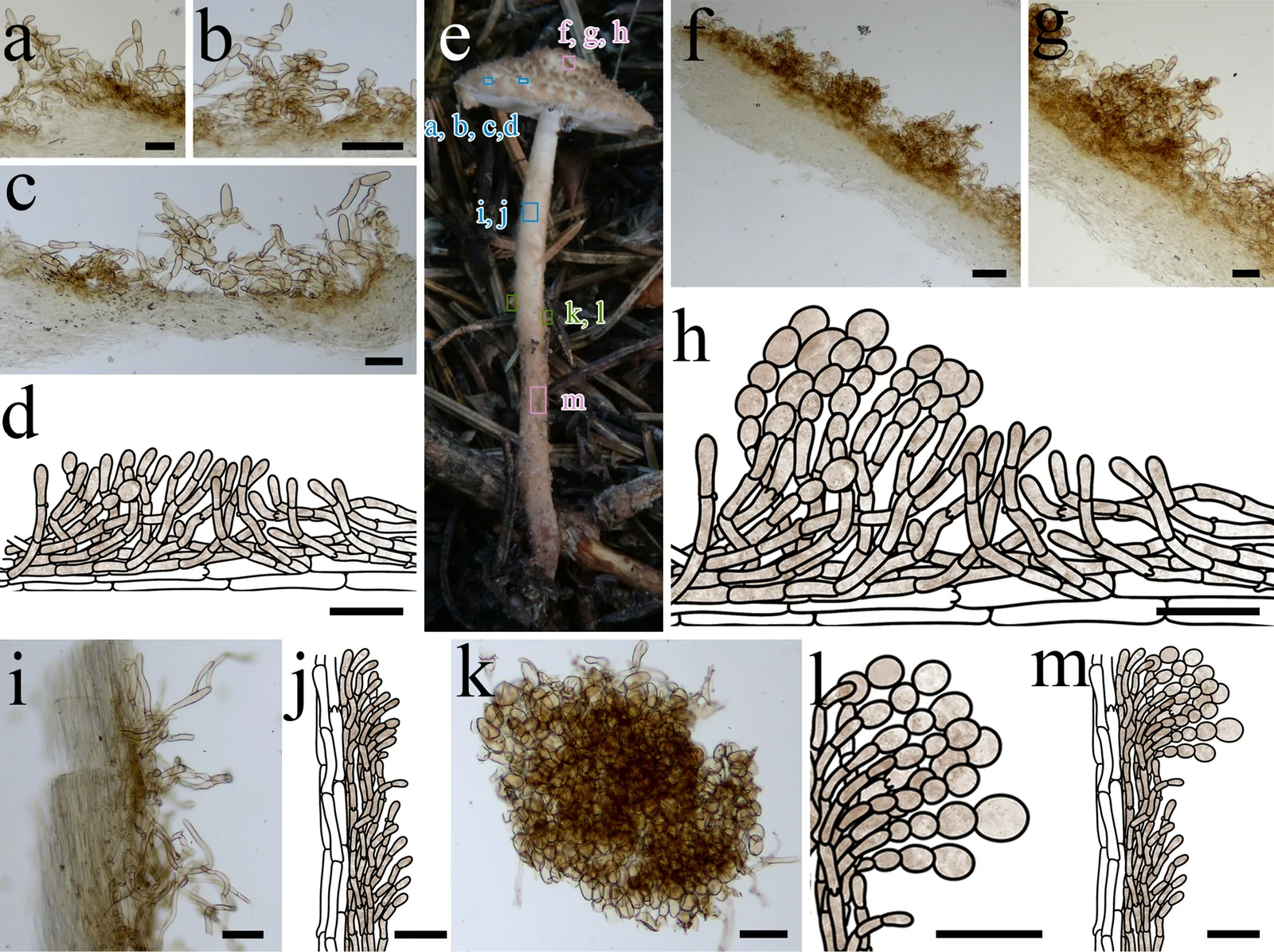
Micromorphology of pileus and stipe coverings in *Lepiota verruculosa*. **a–c**. Fibrous-floccose squamules on pileus surface, composed of inflated cylindrical to ellipsoid cells connected in chains. **d**. Line drawing of the fibrous-floccose pileus squamules. **e**. Basidiomes of *Lepiota verruculosa* (FJAU79259). **f, g**. Pileus covering in section: a three-layered structure with a basal layer of broad, hyaline hyphae, an intermediate layer of inflated cylindrical-ellipsoid catenulate cells, and an apical layer of globose chains. **h**. Line drawing of the pileus covering. **i**. Fibrous-floccose squamules on stipe surface, composed of elongate to inflated hyphae. **j**. Line drawing of the fibrous-floccose stipe squamules. **k**. Echinulate-verrucose squamules on stipe surface, composed of globose cells in chains. **l**. Line drawing of the echinulate-verrucose stipe squamules. **m**. Line drawing of stipe covering: a three-layered structure as in the pileus. Scale bars: 1 cm (**e**); 50 μm (**a–d, f–m**).

**Figure 6.**
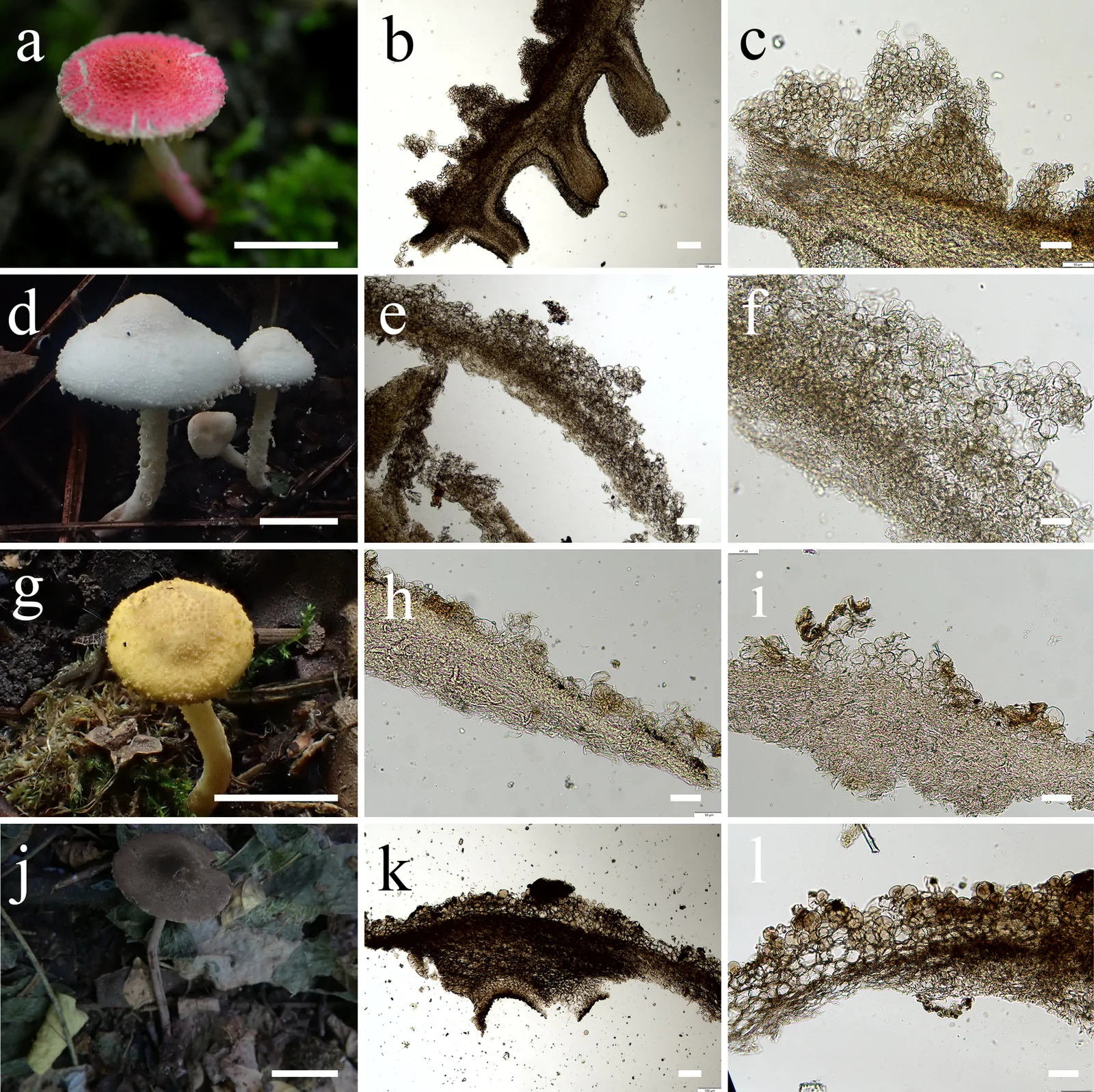
Pileus covering micromorphology in *Lepiota*, *Cystolepiota* and *Melanophyllum* (two-layered type). **a**. *Lepiota squamulosa* (FJAU79306). **b, c**. Pileus covering: two layers, a basal context layer and an upper layer of loosely arranged sphaerocysts not forming chains. **d**. *Lepiota fugaciosquamosa* (FJAU79282). **e, f**. Pileus covering with the same two-layered structure. **g**. *Cystolepiota luteosquamulosa* (FJAU79481). **h, i**. Pileus covering two-layered, sphaerocysts loose, not in chains. **j**. *Melanophyllum haematospermum* (FJAU79299). **k, l**. Pileus covering showing the two-layered structure. Scale bars: 1 cm (**a, d, g, j**); 100 μm (**b, e, k**); 50 μm (**c, f, h, i, l**).

To establish a consistent terminology for subsequent taxonomic descriptions, the pileus and stipe squamule microstructures of *Lepiota* sect. *Eriophorae*, *Echinoderma*, *Cystolepiota* and *Melanophyllum* were examined based on the framework of Knudsen (1978, 1980). Consistent with this framework, two main types of pileus surface squamules can be recognized: Type I (three-layered) and Type II (two-layered) (Figs 4, 5, 6). They are distinguished by the presence or absence of an intermediate fibrous layer, the cellular composition of this layer, the cellular arrangement in the uppermost layer.

Type I – Three-layered structure with apical globose chains (Figs 4, 5). The basal layer consists of broad, hyaline hyphae emerging directly from the pileus or stipe context. The intermediate layer forms the macroscopically visible fibrous-floccose squamules, composed of either narrow hyphae with inflated terminal cells (Fig. 4a–c, h, i) or inflated cylindrical to ellipsoid catenulate cells (Fig. 5a–c). The apical layer comprises globose to subglobose cells arranged in distinct chains, forming the echinulate-verrucose squamules on both pileus and stipe surfaces (Figs 4e–g, 5k, l). Line drawings of the complete three-layered structure of the pileus and stipe are shown in Figs 4j, k, 5h, m. This three-layered structure is exemplified by *Echinoderma petaloides* (Fig. 4) and *Lepiota verruculosa* (Fig. 5).

Based on the cellular composition of the intermediate layer, Type I can be further divided into two subtypes:

Subtype IA: intermediate layer composed of elongate hyphae (Fig. 4).

Subtype IB: intermediate layer composed of inflated cylindrical to ellipsoid catenulate cells (Fig. 5).

Type II – Two-layered pileus covering (Fig. 6). Observed in certain *Lepiota* species, *Cystolepiota* and *Melanophyllum*, this simpler structure lacks an intermediate fibrous layer. It consists solely of the basal context layer and an overlying layer of loosely arranged sphaerocysts that do not form chains (Fig. 6b, c, e, f, h, i, k, l). Macroscopically, these correspond to granulose or fine pulverulent squamules; lacking chain-like cohesion, these squamules are highly friable and deciduous.

**Figure 7.**
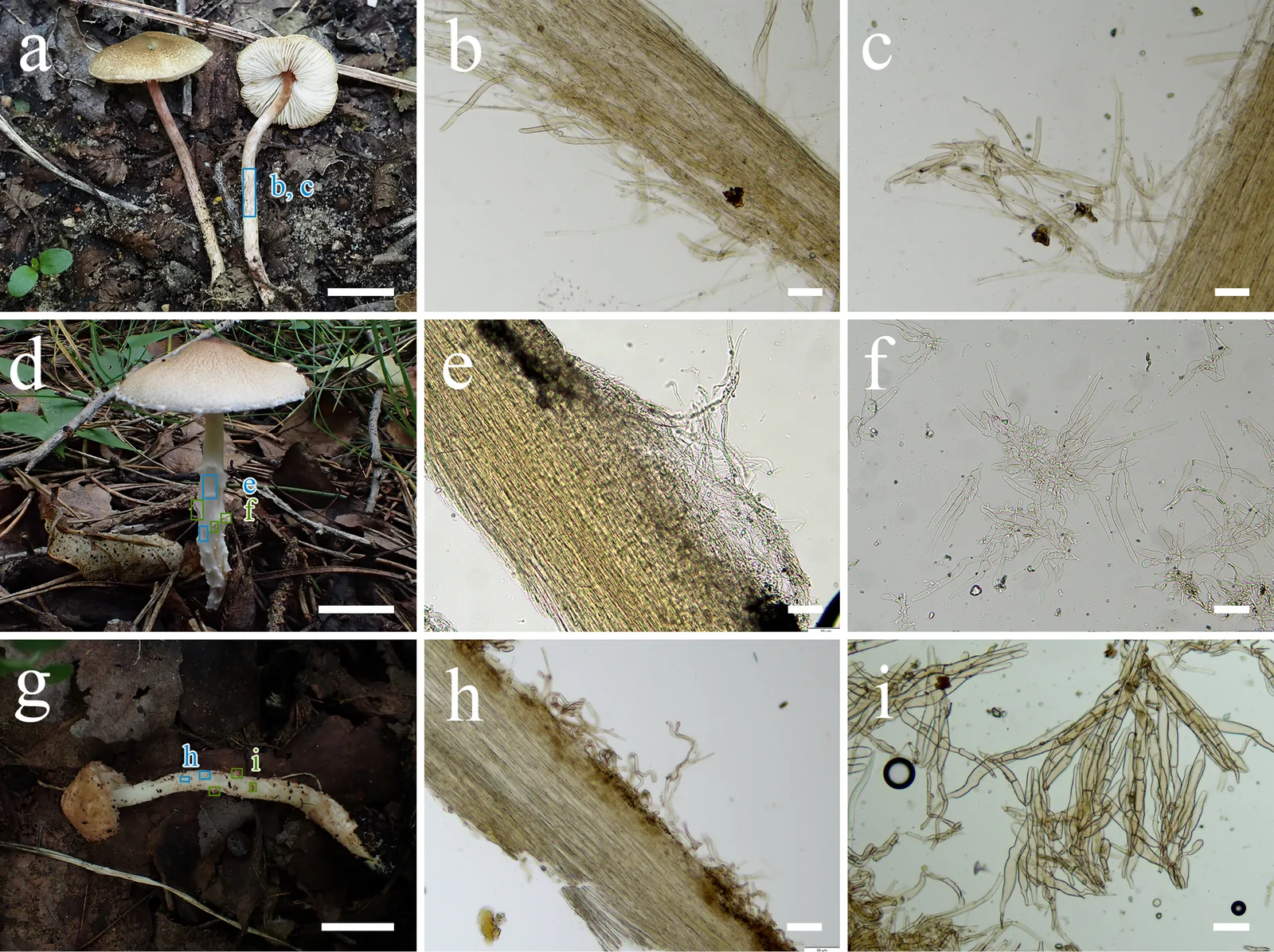
Stipe surface structures in *Lepiota* species. **a**. *Lepiota fulvoolivacea* (FJAU78251). **b, c**. Stipe surface forming a cutis of elongate, narrow hyphae. **d**. *Lepiota subforquignonii* (FJAU79189). **e**. Stipe surface forming a cutis with interwoven hyphae. **f**. Granular-verrucose squamules overlying the fibrous stipe surface, forming a trichoderm with clavate to subcylindrical terminal cells. **g**. *Lepiota spinulosa* (FJAU78319). **h**. Stipe surface forming a cutis with interwoven hyphae. **i**. Stipe surface as in *L. subforquignonii*, with trichoderm-like clusters. Scale bars: 1 cm (**a, d, g**); 50 μm (**b, c, e, f, h, i**).

Stipe covering structures (Fig. 7). In the *Lepiota* species examined, the stipe surface is typically covered by fibrous-floccose squamules composed of a cutis of interwoven, elongate hyphae (Fig. 7b, c, e, h). The granular-verrucose squamules overlying these fibrous elements are microscopically similar to those on the pileus surface, forming a trichoderm of palisade-like hyphae with clavate to subcylindrical terminal cells (Fig. 7f, i).

##### Cystolepiota

Taxon classificationAnimaliaAgaricalesAgaricaceae

Singer, Lilloa 25: 281 (1952)

C65437A0-B171-510C-BDFA-0E703ED851D6

Fig. 8

###### Type species.

*Cystolepiota constricta* Singer, Lilloa 25: 283 (1952).

###### Notes.

Traditionally, this genus has been divided into two sections based on the dextrinoid reaction of basidiospores in Melzer’s reagent: *C.* sect. *Pseudoamyloideae* Singer & Clémençon (dextrinoid) and *C.* sect. *Cystolepiota* Singer (non-dextrinoid) (Singer and Clémençon 1972). However, phylogenetic analyses indicate that neither section forms a monophyletic group (Zhou and Bau 2024). In this study, a novel species of *Cystolepiota* is described.

**Figure 8.**
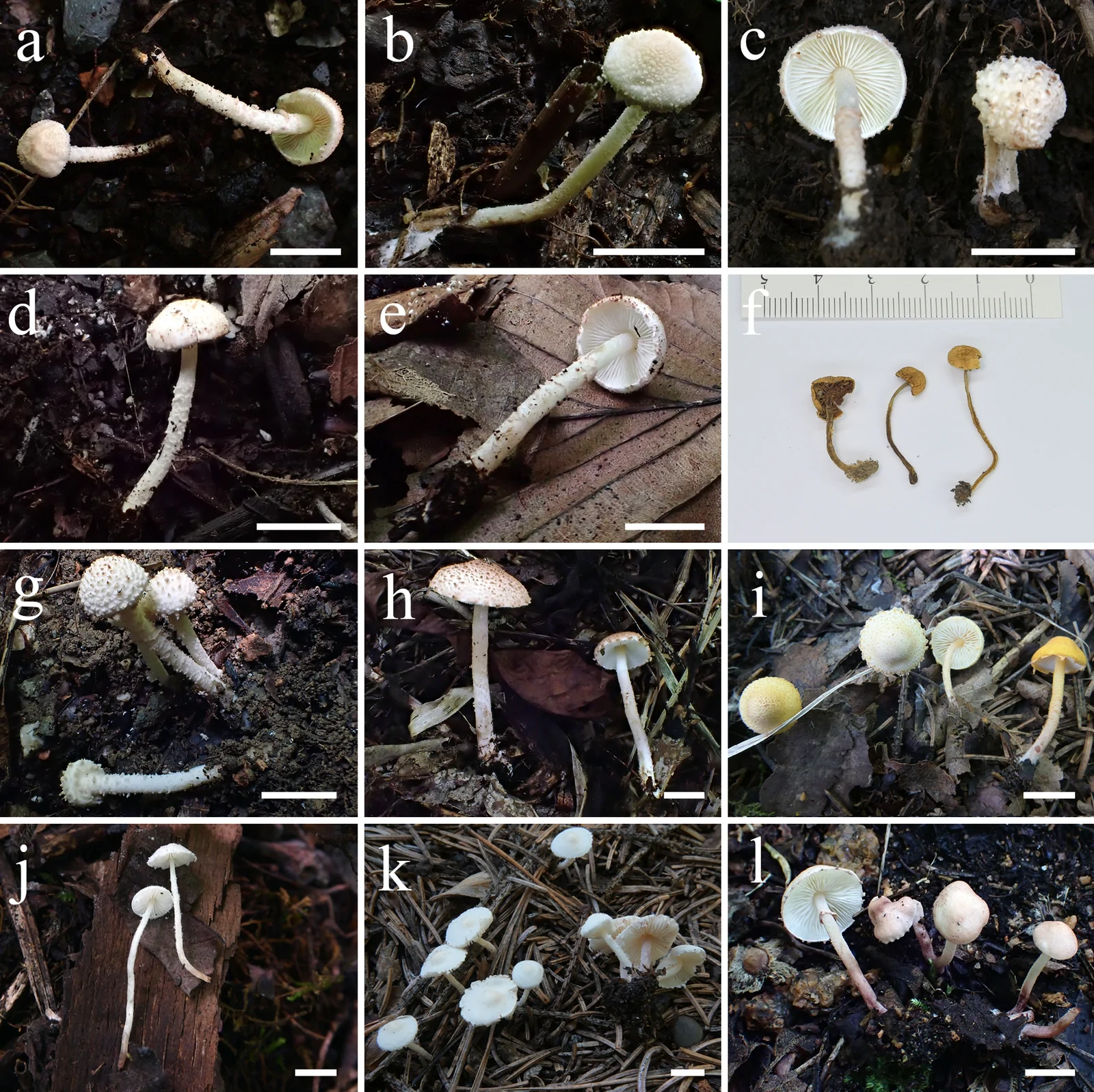
Basidiomata of *Cystolepiota*. **a–f**. *Cystolepiota mastoidea* (**a** FJAU79287; **b** HMJAU68235; **c** FJAU79286; **d, e** FJAU79288; **f** from left to right: FJAU79286, FJAU79288, FJAU79285); **g**. *Cystolepiota changbaishanensis* (FJAU79476); **h**. *Cystolepiota beijingensis* (FJAU79303); **i**. *Cystolepiota luteosquamulosa* (FJAU79477); **j**. *Cystolepiota pseudoseminuda* (FJAU9478); **k**. *Cystolepiota seminuda* (FJAU49479); **l**. *Cystolepiota aff. seminuda* (FJAU79480). Scale bars: 1 cm.

##### Cystolepiota mastoidea

Taxon classificationAnimaliaAgaricalesAgaricaceae

T. Bau & X.Y. Zhou sp. nov.

9DC89A89-E369-5113-9ABB-CB0CDC7E43CE

Index Fungorum: IF906076

Figs 8a–f, 9a, 10

###### Etymology.

“*mastoidea*” (from Greek mastos, meaning breast or nipple, and Latin suffix -oideus, meaning resembling) refers to the distinct nipple-like or breast-shaped apex of the cheilocystidia.

###### Holotype.

CHINA • Liaoning: Benxi city, Tuotanggou Greenstone Valley National Forest Park, 41°4'8"N, 124°12'59"E, alt. ca. 560 m, coll. L.H. Li, FJAU79288.

###### Diagnosis.

*Cystolepiota mastoidea* is macroscopically similar to *C. changbaishanensis*, but differs significantly by its smaller, ellipsoid to cylindrical basidiospores (3.5–4.2 × 1.7–2.4 μm) and mostly lecythiform to clavate cheilocystidia with a capitate apex.

###### Description.

Basidiomata small. Pileus 0.8–1.4 cm in diam., campanulate to hemispherical when young, becoming hemispherical to plano-convex with age; surface white to cream, densely covered with orange white (6A2) to pinkish white (7A2–A3) verrucose to granular squamules, squamules darker at the center; margin occasionally with velar remnants, concolorous with the squamules. Lamellae free, white to cream, crowded; lamellulae in 3 tiers; edge concolorous. Stipe 0.3–3.5 × 0.2–0.3 cm, subcylindrical, slightly broadening towards the base, cream; annular zone present; nearly glabrous above the annular zone, densely covered with concolorous to pinkish white granular squamules below. Context in pileus white, thin, unchanging; in stipe cream, turning reddish brown (9E5–E8) towards base. Upon drying, the pileus and stipe turn orange (6A5–A7), and the lamellae turn brownish orange (5C5–C7). Odor and taste not recorded.

**Figure 9.**
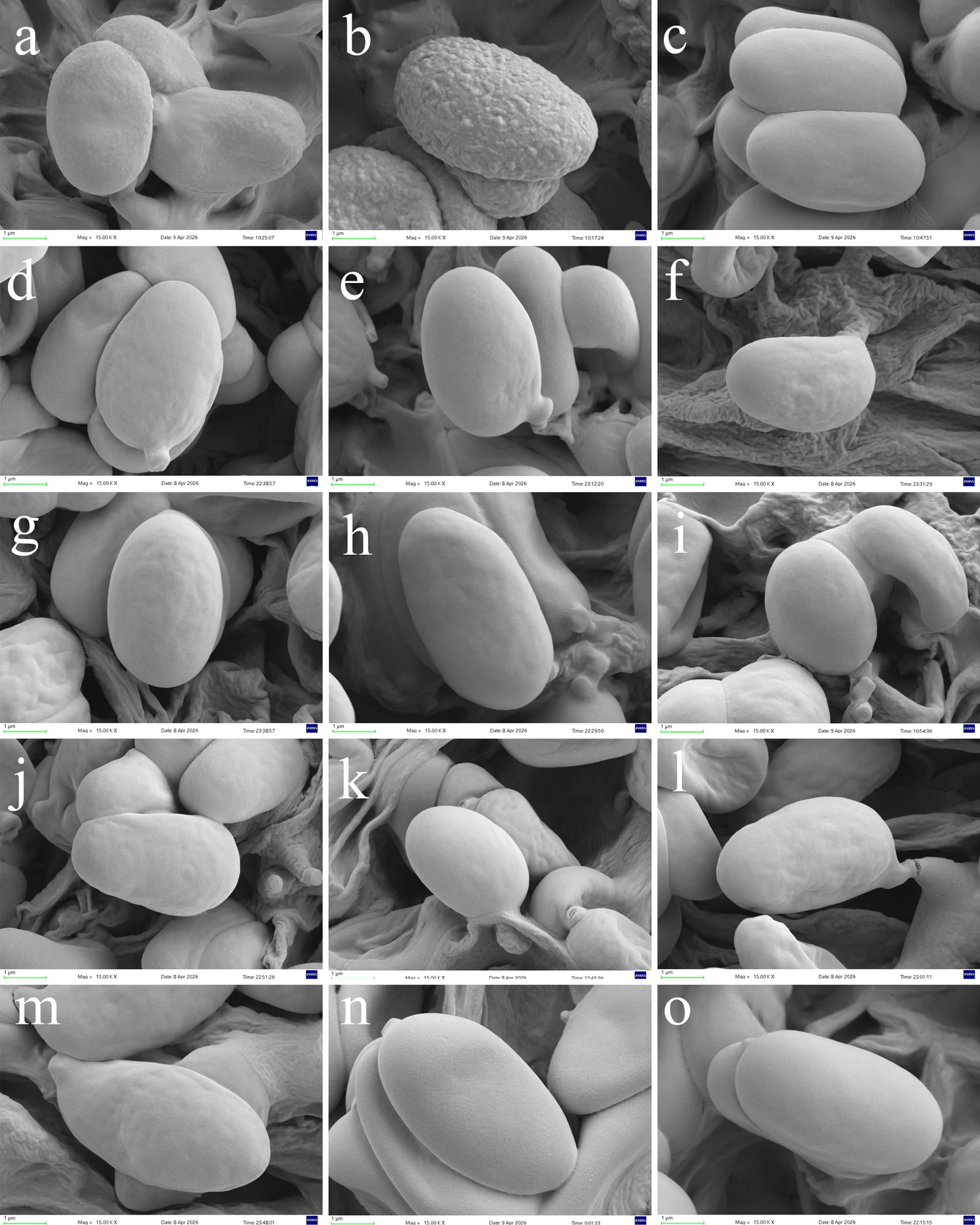
Basidiospores of *Cystolepiota*, *Echinoderma*, *Lepiota*, and *Melanophyllum* species under SEM. **a**. *Cystolepiota mastoidea* (FJAU79288); **b**. *Melanophyllum aff. haematospermum* (FJAU79289); **c**. *Lepiota flavocarpa* (FJAU79333); **d**. *L. granulosquama* (FJAU79253); **e**. *L. huadianensis* (FJAU79268); **f**. *L. aff. jacobi* 1 (FJAU79321); **g**. *L. aff. jacobi* 2 (FJAU79323); **h**. *L. scabra* (FJAU79248); **i**. *L. squamulosa* (FJAU79306); **j**. *L. subhuadianensis* (FJAU79264); **k**. *L. verruculosa* (FJAU79255); **l**. *L. fugaciosquamosa* (FJAU79282); **m**. *Echinoderma asperum* 1 (HMJAU37543); **n**. *E. asperum* 2 (FJAU79273); **o**. *E. petaloides* (FJAU79274).

Basidiospores [40/2/2] 3.5–4.2 × 1.7–2.4 μm, avl × avw = 3.8 × 2.0 μm, Q = 1.48–2.14, Qav = 1.82–1.88; ellipsoid to cylindrical; non-dextrinoid, metachromatic, thin-walled; smooth under LM, with small warts under SEM. Basidia 12–20 × 4–8 μm, clavate, grayish yellow (4B3–B5), thin-walled, 4-spored, occasionally 2-spored. Cheilocystidia 21–45 × 6–11 μm, lecythiform to clavate or broadly clavate with a capitate apex, grayish yellow (4B3–B8), slightly thick-walled. Pleurocystidia not observed. Pileus covering 2-layered: basal hyphae hyaline, thin-walled; apical layer composed of sparsely arranged hyphae and abundant inflated cells; inflated cells subglobose, obovoid, oblong to sphaeropedunculate, sometimes 2–5 connected in short moniliform chains, easily detachable, 12–58 μm, with brownish orange (6C4–C7) intracellular pigments. Stipe covering similar to pileus covering in layer structure, cellular shape, and pigmentation. Clamp connections present.

**Figure 10.**
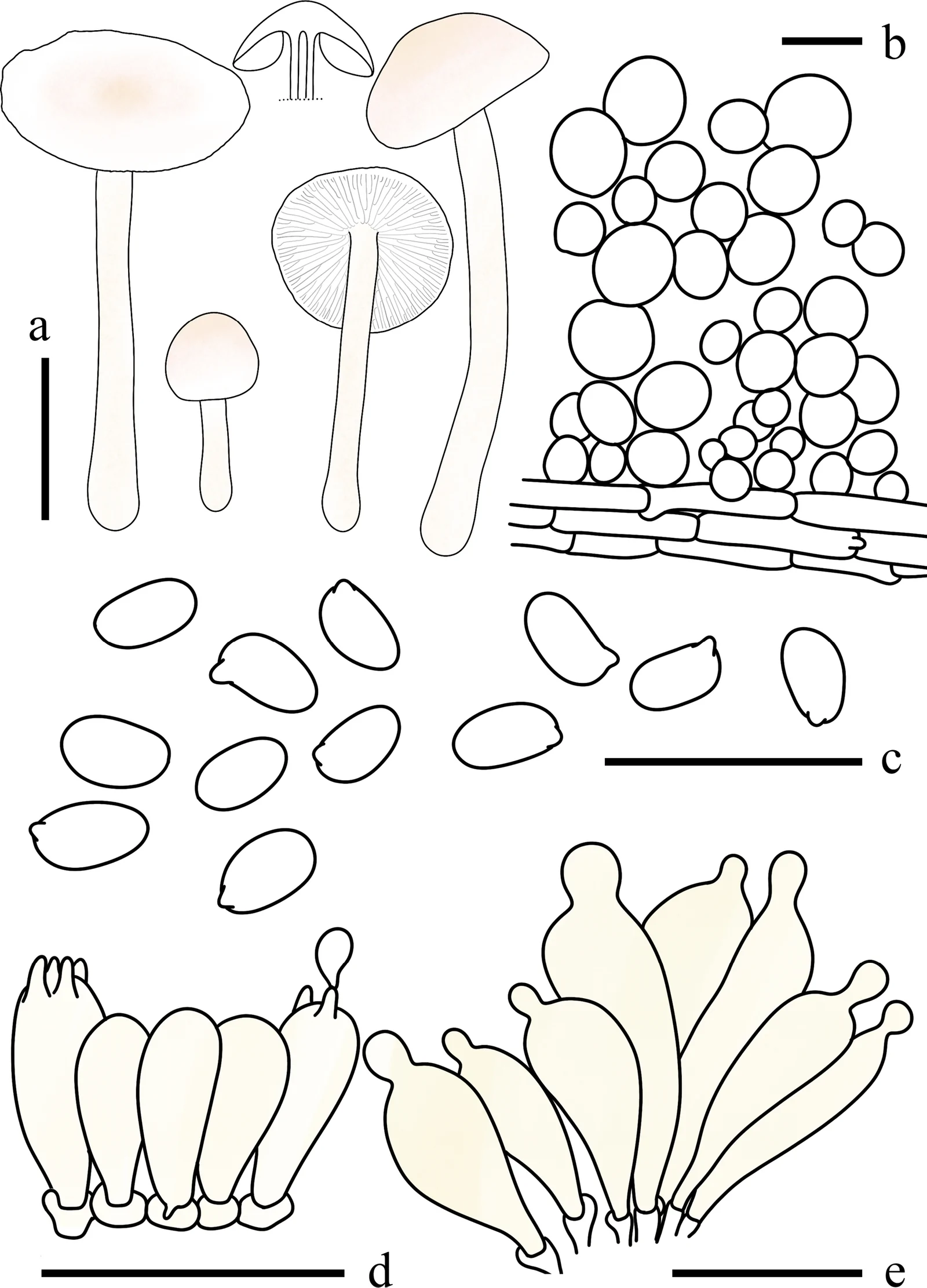
*Cystolepiota mastoidea* (FJAU79288). **a**. Basidiomata. **b**. Pileus covering. **c**. Basidiospores. **d**. Basidia and basidioles. **e**. Cheilocystidia. Scale bars: 1 cm (**a**); 50 μm (**b**); 10 μm (**c**); 20 μm (**d, e**).

###### Habit and habitat.

Solitary to scattered on humus soil in broad-leaved forests.

###### Distribution.

Jilin and Liaoning Province, China.

###### Additional specimens examined.

CHINA • Jilin: Dunhua City, State-Owned Forest farm, 27 July 2022, coll. W.N. Hou, HMJAU68235; Huadian City, Hongshi National Forest Park, 42°50'01"N, 127°07'57"E, alt. ca. 490 m, 23 August 2024, coll. T.Y. Zhang, FJAU79285, Liaoning: Benxi city, Tuotanggou Greenstone Valley National Forest Park, 41°4'8"N, 124°12'59"E, alt. ca. 560 m, coll. H. Cheng and X.Y. Zhou, FJAU79286, FJAU79287.

###### Notes.

Phylogenetically, *Cystolepiota mastoidea* clusters with *C. contusifolia* (Pegler) I. Saar and *C. nivalis* T. Bau & X.Y. Zhou in a single clade. However, these species can be readily separated based on morphological characteristics. *Cystolepiota nivalis* differs by having white basidiomata that do not discolor upon drying, as well as smooth basidiospores under SEM (Zhou and Bau 2024). Although *C. contusifolia* shares the presence of lecythiform cheilocystidia, its cheilocystidia are hyaline, whereas those of *C. mastoidea* are grayish-yellow. Additionally, *C. contusifolia* is not reported to exhibit basidiomata discoloration upon drying (Saar et al. 2024).

Macroscopically, *Cystolepiota mastoidea* is most similar to *C. changbaishanensis* T. Bau & X.Y. Zhou. Both species share pale pileus squamules that turn to a grayish-orange tone upon drying, and white lamellae that become pale brown when dried. Additionally, under SEM, both exhibit fine warts on the basidiospore surface (Zhou and Bau 2024).

However, they can be clearly distinguished by their microscopic features. First, the basidiospores of *C. changbaishanensis* [4.6–6.0(–6.4) × 2.1–3.0 μm] are significantly larger than those of *C. mastoidea* (3.5–4.2 × 1.7–2.4 μm). Furthermore, the cheilocystidia of *C. changbaishanensis* are lageniform to broadly lageniform with a distinctively long apical neck (Zhou and Bau 2024), whereas those of *C. mastoidea* are lecythiform or clavate to broadly clavate, typically featuring a capitate apex or possessing only a short neck.

##### Echinoderma

Taxon classificationAnimaliaAgaricalesAgaricaceae

(Locq. ex Bon) Bon, Doc. Mycol. 21 (82): 61 (1991)

23D962A7-20C6-52BE-B77D-084879D53372

Fig. 11

###### Type species.

*Echinoderma acutesquamosum* (Weinm., nom. sanct. Fr.) Bon (= *Echinoderma asperum* (Pers., nom. sanct. Fr.) Bon).

###### Notes.

Currently, the genus *Echinoderma* contains only six formally described species: *E. asperum* (Pers.) Bon, *E. flavidoasperum* Y.J. Hou & Z.W. Ge, *E. hystrix* (F.H. Møller & J.E. Lange) Bon, *E. perplexum* (Knudsen) Bon, *E. calcicola* (Knudsen) Bon, and *E. petaloides* (Hou and Ge 2020; Sarawi et al. 2025a; Xu et al. 2026).

**Figure 11.**
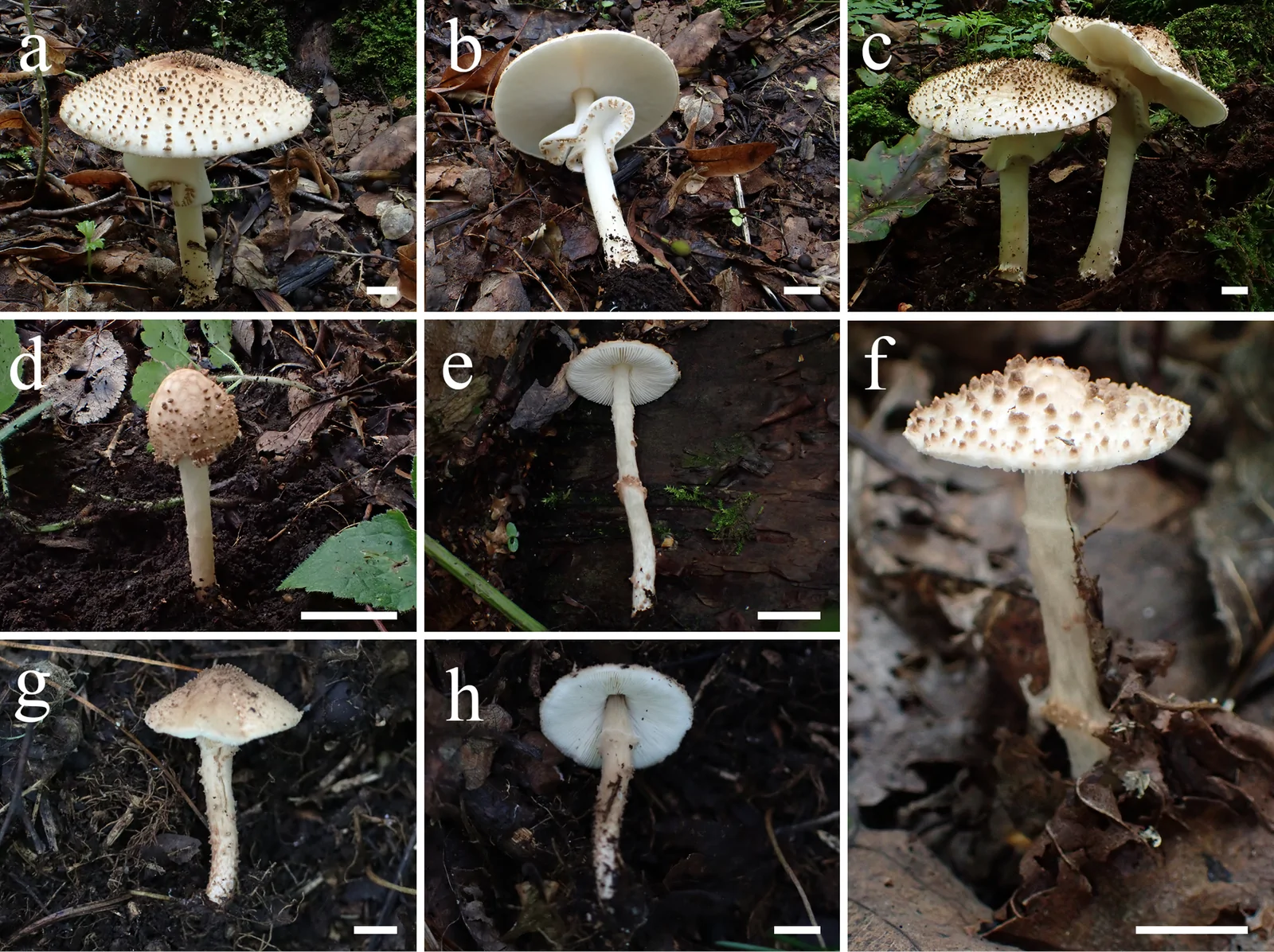
Basidiomata of *Echinoderma* species. **a–c**. *Echinoderma aff. asperum* 2 (**a, b** FJAU79273; **c** FJAU79270); **d–h**. *Echinoderma petaloides* (**d** FJAU79279; **e, f** FJAU79274; **g, h** FJAU79275). Scale bars: 1 cm.

##### Lepiota

Taxon classificationAnimaliaAgaricalesAgaricaceae

(Pers.) Gray, Nat. Arr. Brit. Pl. (London) 1: 601 (1821)

8AE1E48F-A5A8-5FC6-A2A6-9BF3025821C6

Figs 12, 13, 14, 15, 16, 17, 18, 19, 20, 21

###### Type species

**(typ. cons.)**. *Agaricus colubrinus* Pers. [= *Lepiota clypeolaria* (Bull.) P. Kumm.].

**Figure 12.**
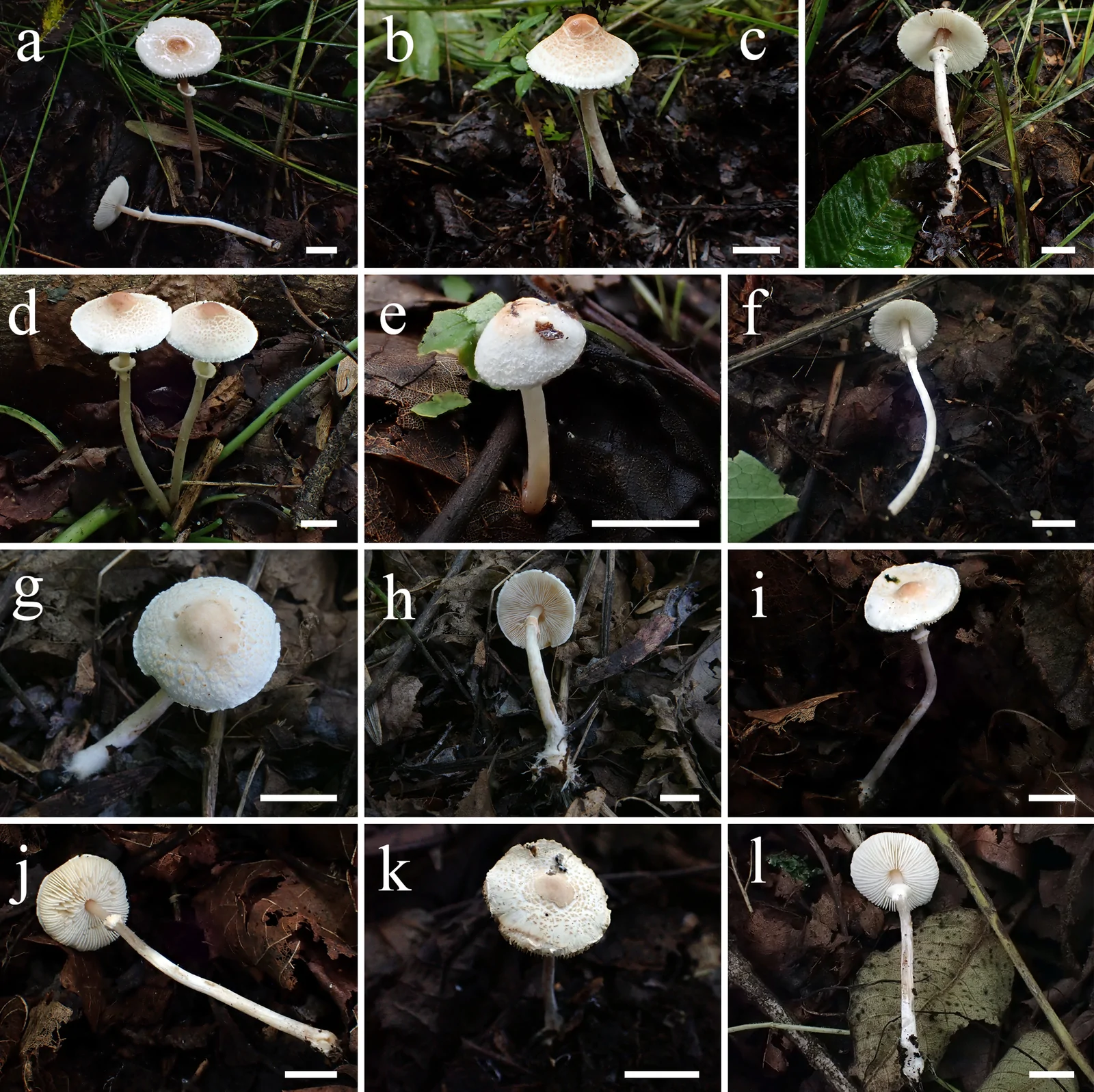
Basidiomata of *Lepiota* sect. *Cristatae* species. **a–c**. *Lepiota cristatosimilis* (**a** FJAU78310, **b, c** FJAU78311); **d–f**. *Lepiota aff. cristata* (**d** FJAU78307, **e** FJAU78304, **f** FJAU78306); **g–l**. *Lepiota recondita* (**g, h** FJAU78325, **i, j** FJAU78326, **k** FJAU78328, **l** FJAU78327). Scale bars: 1 cm.

**Figure 13.**
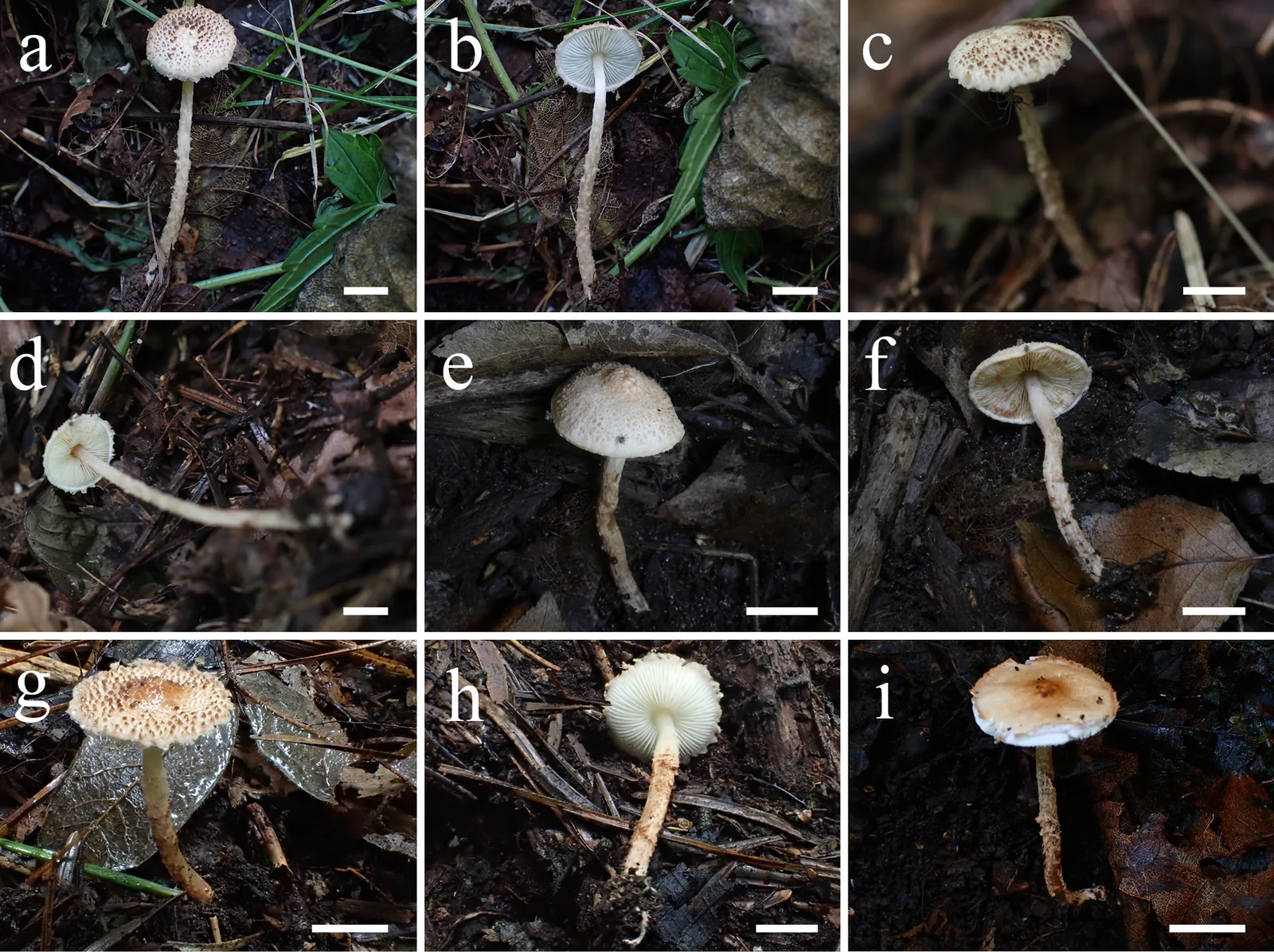
Basidiomata of *Lepiota* sect. *Eriophorae* species. **a–f**. *Lepiota granulosquama* (**a, b** FJAU79253, **c, d** FJAU79252, **e, f** FJAU79254); **g–i**. *Lepiota huadianensis* (**g, h** FJAU79267, **i** FJAU79268). Scale bars: 1 cm.

**Figure 14.**
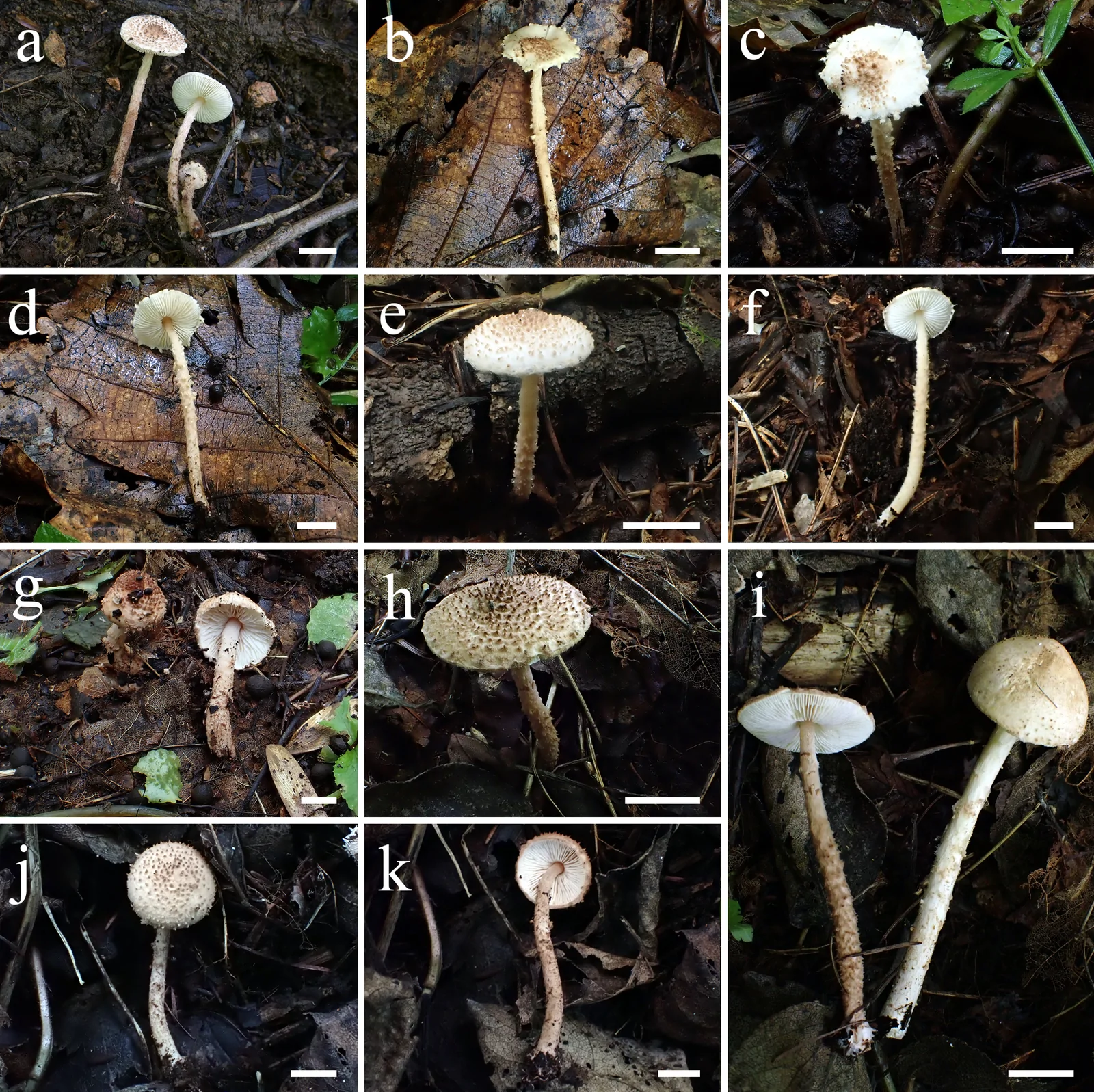
Basidiomata of *Lepiota* sect. *Eriophorae* species. **a–f**. *Lepiota scabra* (**a** FJAU79248, **b–d** FJAU79250, **e, f** FJAU79249); **g–k**. *Lepiota subhuadianensis* (**g** FJAU79264, **h, i** FJAU79263, **j, k** FJAU79265). Scale bars: 1 cm.

**Figure 15.**
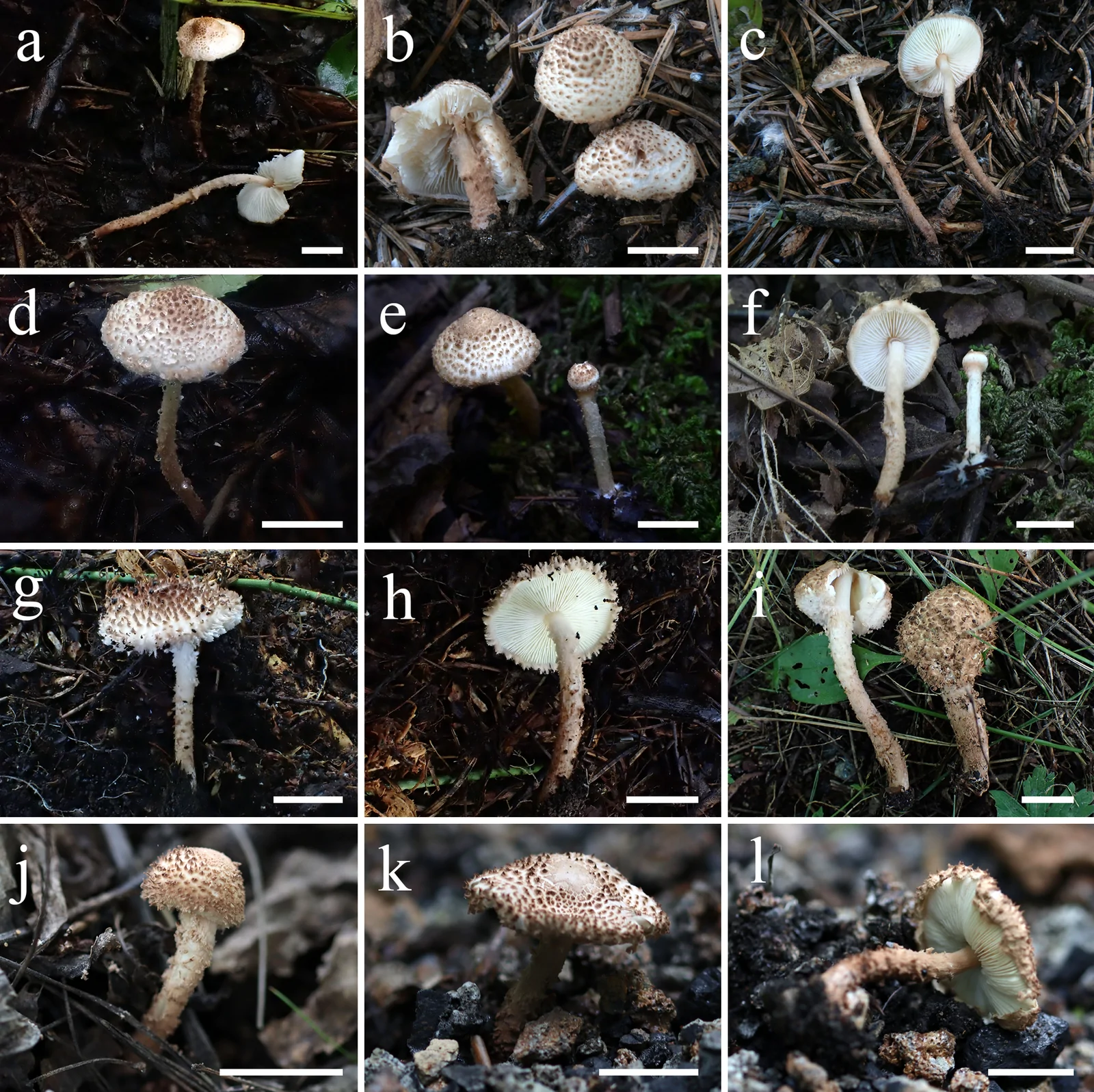
Basidiomata of *Lepiota* sect. *Eriophorae* species. **a–f**. *Lepiota verruculosa* (**a** FJAU79255, **b** FJAU79260, **c** FJAU79259, **d** FJAU79258, **e, f** FJAU79261); **g, h**. *Lepiota aff. jacobi* 1 (FJAU79321); **i–l**. *Lepiota aff. jacobi* 2 (**i** FJAU79327, **j** FJAU79325, **k, l** FJAU79323). Scale bars: 1 cm.

###### Notes.

In this study, 11 major clades within *Lepiota* were identified, corresponding to seven formally defined sections and four unplaced transitional lineages. Sixteen novel *Lepiota* species are described below, distributed across the following sections: sect. *Cristatae* (one new species), sect. *Eriophorae* (six new species, including one within the newly established subsect. *Pseudocystolepiotae*), sect. *Fuscovinaceae* (one new species), sect. *Lepiota* (four new species), and sect. *Stenosporae* (four new species).

**Figure 16.**
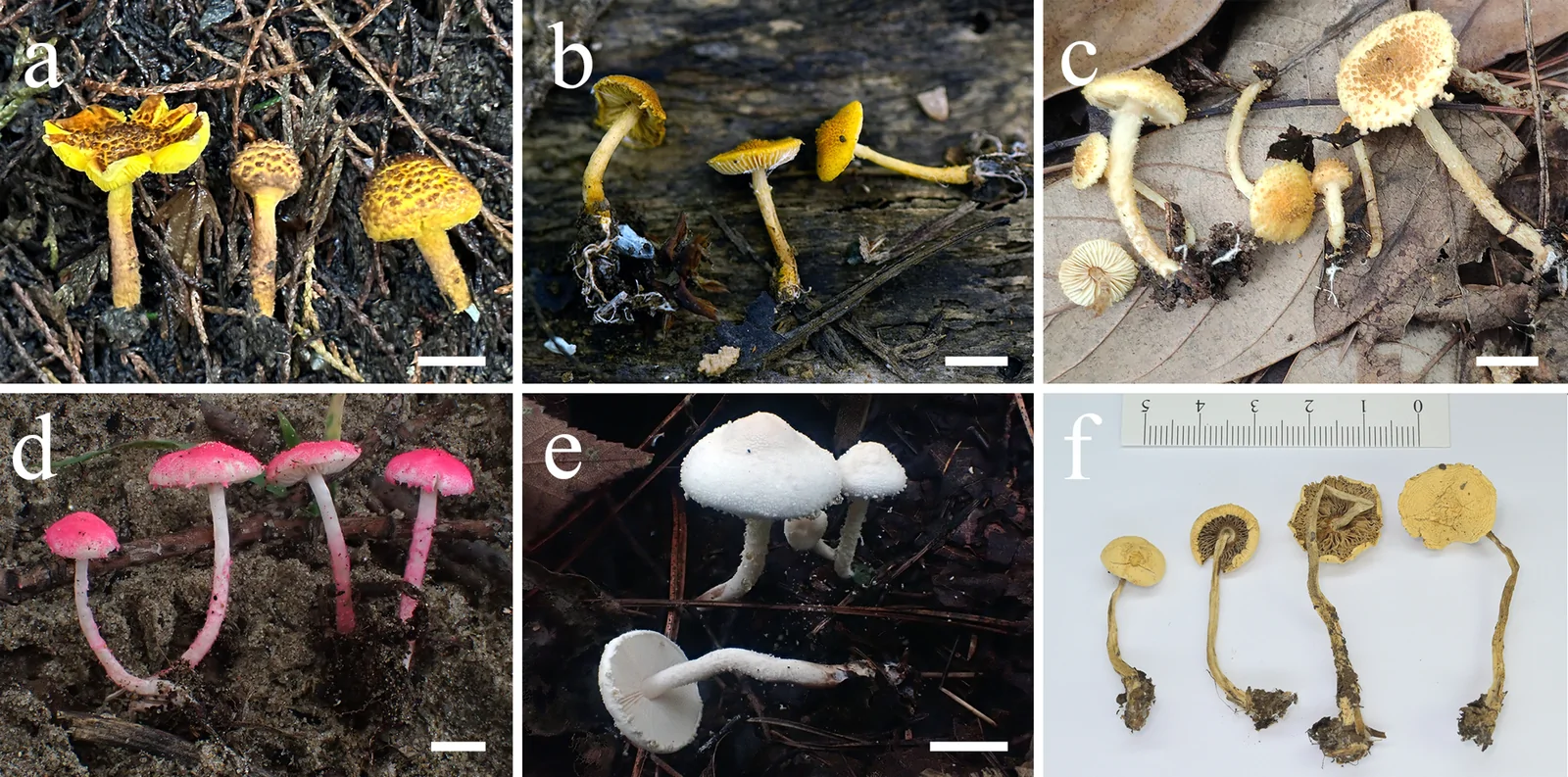
Basidiomata of *Lepiota* sect. *Eriophorae* species. **a**. *Lepiota flavocarpa* (FJAU79332); **b, c**. *Lepiota omninoflava* (**b** FJAU79331, **c** FJAU79330); **d**. *Lepiota squamulosa* (FJAU79475); **e, f**. *Lepiota fugaciosquamosa* (**e** FJAU79282, **f** left two FJAU79282, right two FJAU79281). Scale bars: 1 cm.

**Figure 17.**
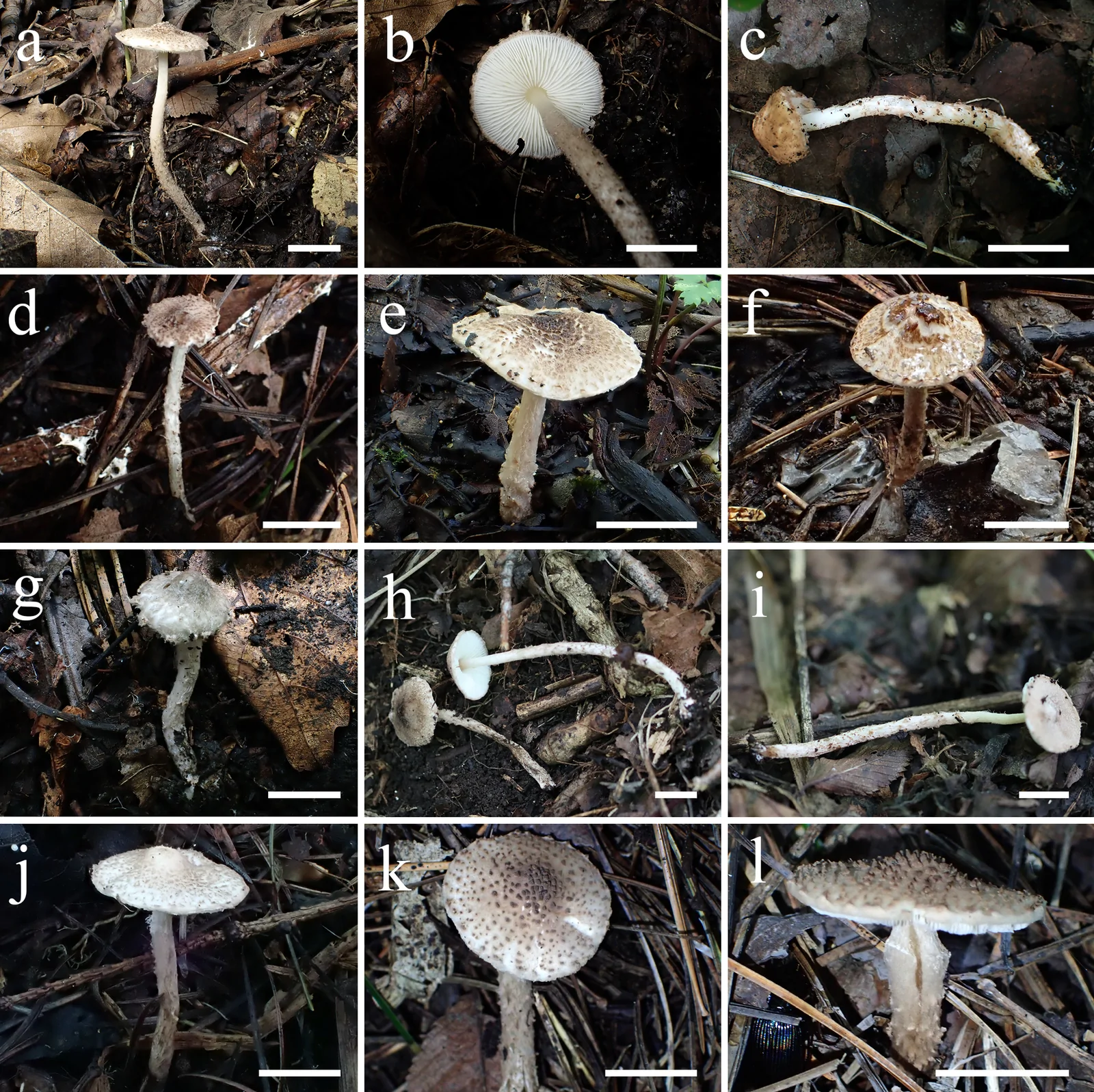
Basidiomata of *Lepiota spinulosa*. **a, b**. FJAU78314; **c**. FJAU78319; **d**. FJAU78321; **e**. FJAU78317; **f**. FJAU78323; **g**. FJAU78315; **h**. FJAU78316; **i**. FJAU78322; **j**. FJAU78320; **k, l**. FJAU78318. Scale bars: 1 cm.

**Figure 18.**
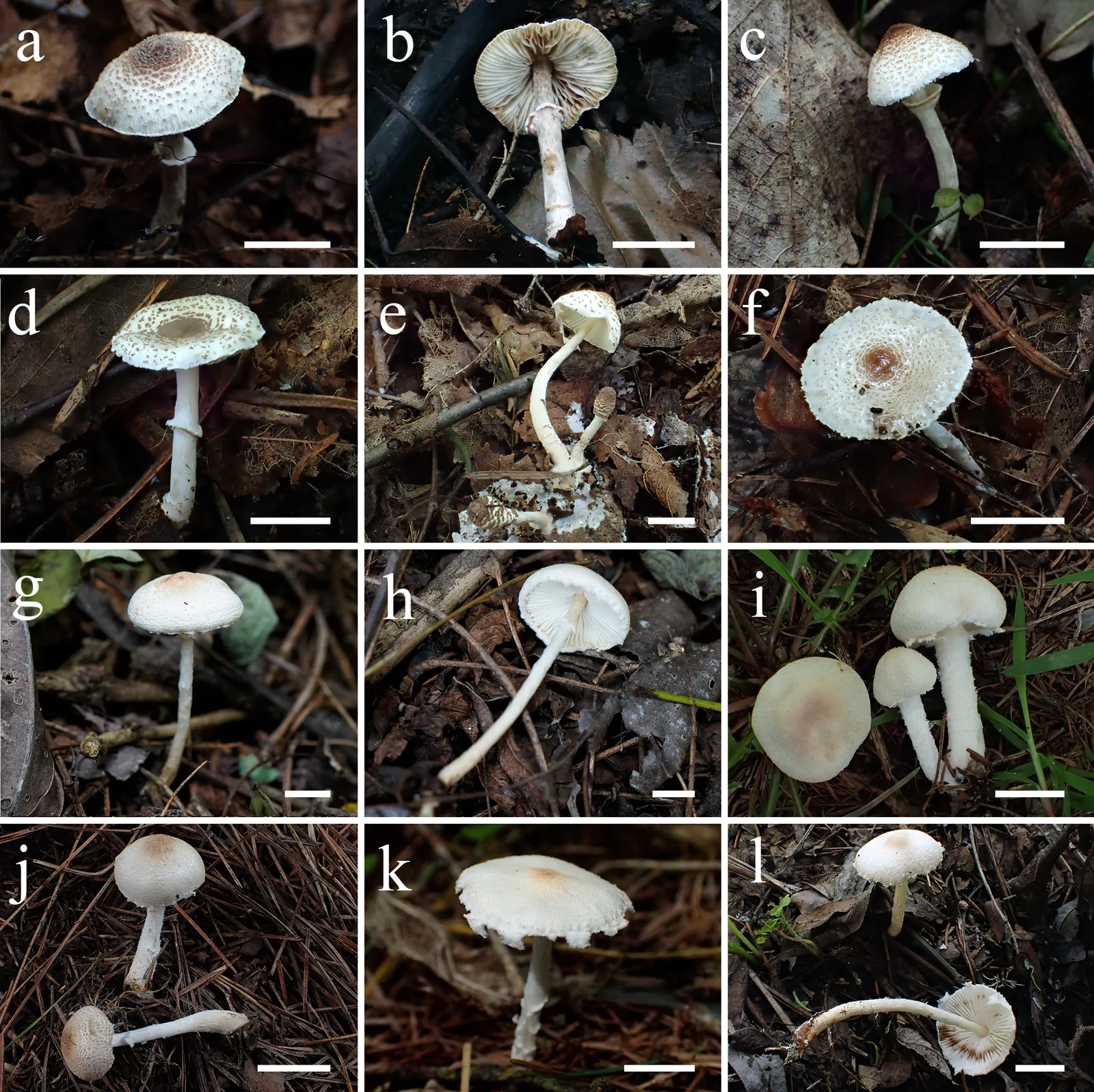
Basidiomata of *Lepiota* sect. *Lepiota* species. **a–f**. *Lepiota basozonata* (**a, b** FJAU79181, **c** FJAU79185, **d** FJAU79186, **e** FJAU79187, **f** FJAU79183); **g–l**. *Lepiota pallidovelata* (**g, h** FJAU79167, **i** FJAU79170, **j** FJAU79171, **k** FJAU79169, **l** FJAU79168). Scale bars: 1 cm.

**Figure 19.**
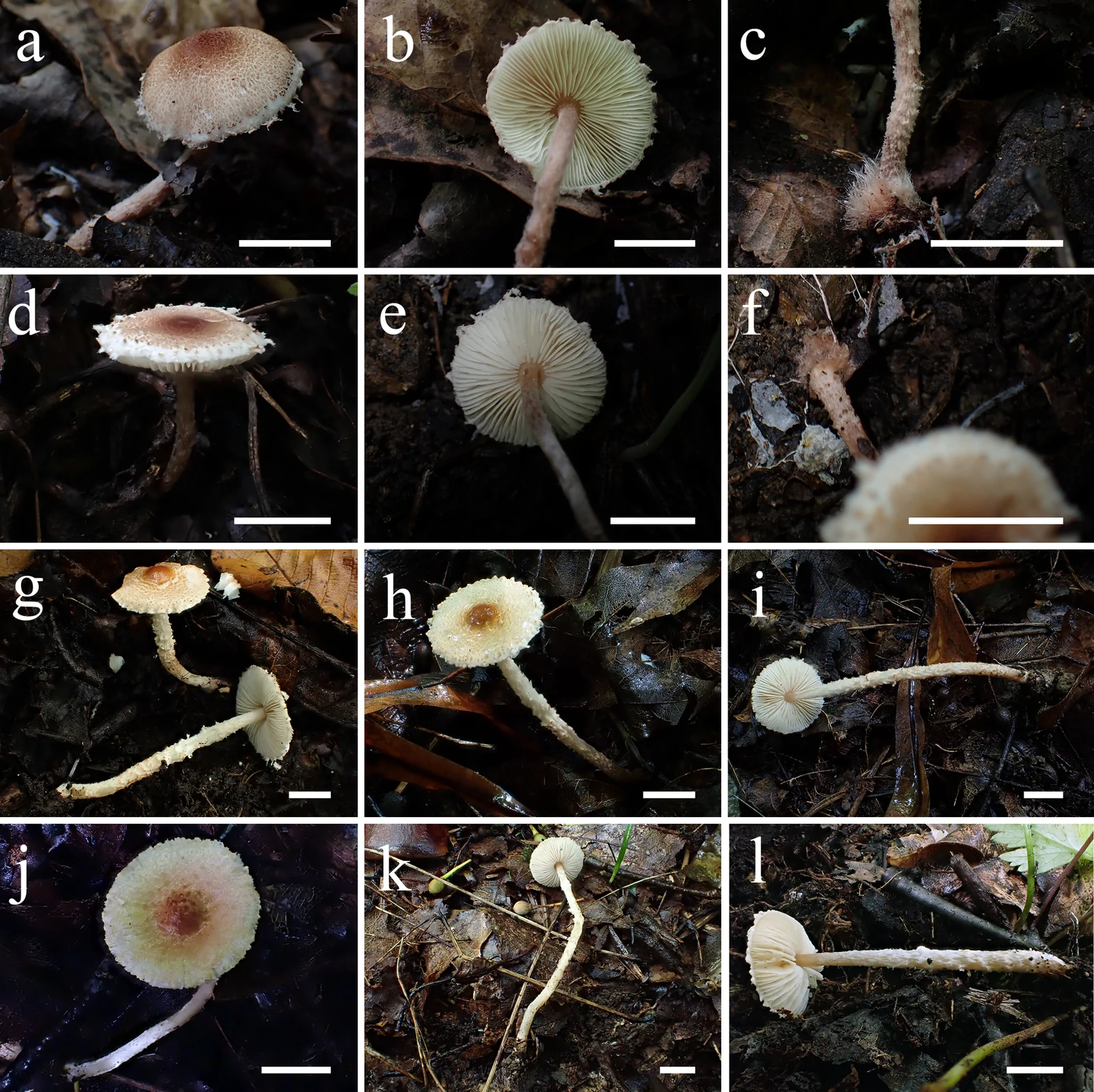
Basidiomata of *Lepiota* sect. *Lepiota* species. **a–f**. *Lepiota jianensis* (**a–c** FJAU79175, **d–f** FJAU79174); **g–l**. *Lepiota aff. echinella* (**g** FJAU79320, **h, i** FJAU79335, **j, k** FJAU79336, **l** FJAU79334). Scale bars: 1 cm.

**Figure 20.**
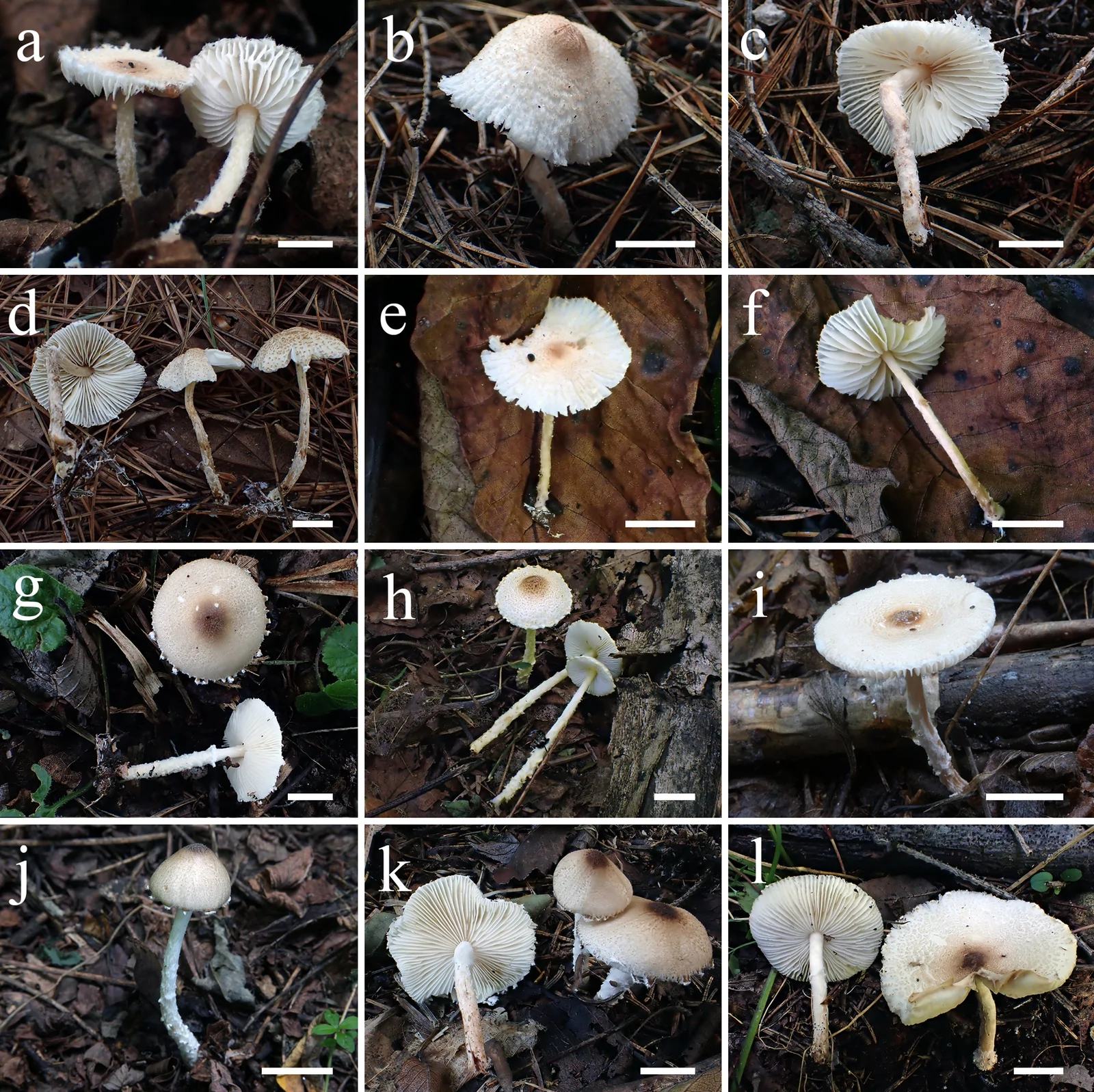
Basidiomata of *Lepiota* sect. *Lepiota* species. **a–f**. *Lepiota rostratospora* (**a** FJAU79178, **b, c** FJAU79177, **d** FJAU79180, **e, f** FJAU79179); **g–l**. *Lepiota subforquignonii* (**g** FJAU79190, **h** FJAU79216, **i** FJAU79200, **j** FJAU79202, **k** FJAU79198, **l** FJAU79192). Scale bars: 1 cm.

**Figure 21.**
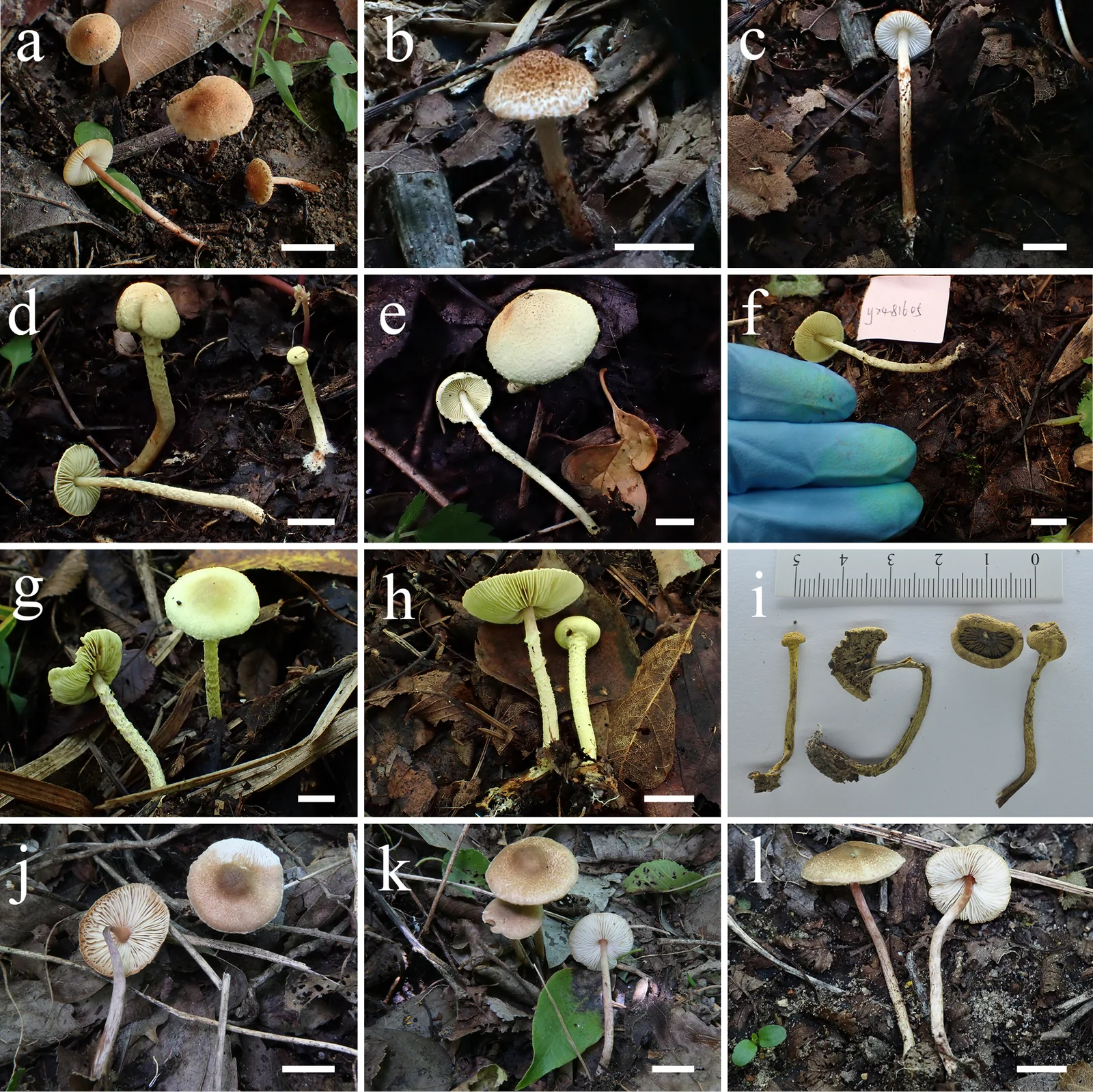
Basidiomata of *Lepiota* sect. *Stenosporae* species. **a**. *Lepiota brunneotacta* (FJAU78296); **b, c**. *Lepiota changbaiensis* (FJAU78297); **d–i**. *Lepiota flavoviridis* (**d, i** FJAU78219, **e** FJAU78234, **f** FJAU78241, **g** FJAU78239, **h** FJAU78240); **j–l**. *Lepiota fulvoolivacea* (**j, k** FJAU78250, **l** FJAU78251). Scale bars: 1 cm.

Furthermore, based on our blast (Suppl. material 2), two species previously placed in *Lepiota* are shown to nest within *Leucocoprinus*. Consequently, they are formally transferred to *Leucocoprinus* as new combinations below.

##### Leucocoprinus booloola

Taxon classificationAnimaliaAgaricalesAgaricaceae

(Grgur.) T. Bau comb. nov.

3D141568-23E2-554C-A4EF-BA567F21C9E0

865517

###### Basionym.

*Lepiota booloola* Grgur., Larger Fungi of South Australia: 457 (1997) [MycoBank No: 443376].

##### Leucocoprinus gomezii

Taxon classificationAnimaliaAgaricalesAgaricaceae

(Singer) T. Bau comb. nov.

D19DDC0C-2DAC-5354-9413-5BAE8B97A891

865518

###### Basionym.

*Lepiota gomezii* Singer, Fieldiana Bot. 21: 99 (1989) [MycoBank No: 124873].

##### Lepiota

Taxon classificationAnimaliaAgaricalesAgaricaceae

sect. Cristatae Kühner ex Wasser, Ukr. Bot. J. 35: 517.

33A2A47A-B643-5A2A-B881-FF236DA6A7C1

###### Type species.

*Lepiota cristata* (Bolton, nom. sanct. Fr.) P. Kumm.

##### Lepiota cristatosimilis

Taxon classificationAnimaliaAgaricalesAgaricaceae

T. Bau & X.Y. Zhou sp. nov.

8B69D76C-B10D-5FDE-9DFE-BE294506EFAB

Index Fungorum: IF905872

Figs 12a–c, 23

###### Etymology.

“*cristatosimilis*” refers to its strong macroscopic resemblance to *Lepiota cristata*, with which it is easily confused in the field due to the similar pileus ornamentation and size.

###### Holotype.

CHINA • Jilin: Jiaohe City, Qianjin Forest Farm, 43°57'10"N, 127°42'7"E, alt. ca. 650 m, 24 July 2022, coll. T. Bau FJAU78310.

###### Diagnosis.

*Lepiota cristatosimilis* is macroscopically similar to the *L. cristata* complex, but is readily distinguished by its smaller basidiomata (pileus < 3 cm) and its distinctly congophilous, rectangular, and nodulose basidiospores (5.4–6.6 × 2.2–3.1 μm).

###### Description.

Basidiomata small. Pileus 1.5–2.7 cm in diam., plano-convex; surface white, center with a reddish orange (7C6–C8) to brownish orange (6C6–C8) intact disc, progressively disrupting towards the margin into somewhat concentrically arranged, blocky small squamules; margin with velar remnants. Lamellae free, white to cream, crowded, with 2–3 tiers of lamellulae; edge concolorous. Stipe 3.6–6.2 × 0.2–0.4 cm, subcylindrical, slightly broadening towards the base, white. Context in pileus white, thin, unchanging when bruised; in stipe reddish brown (8E5–E8). Annulus superior, white, membranous. Odor and taste not recorded.

Basidiospores [40/2/2] 5.4–6.6 × 2.2–3.1 μm, avl × avw = 5.9 × 2.6 μm, Q = 2.02–2.73, Qav = 2.27, rectangular with indistinct knobs, sub-triangular in side view, bilaterally convex in frontal view, non-dextrinoid, smooth, congophilous, non-metachromatic. Basidia 14–22 × 5–8 μm, clavate, thin-walled, 4-spored, occasionally 2-spored. Cheilocystidia 22–41 × 7–11 μm, clavate, thin-walled. Pleurocystidia not observed. Pileus covering hymeniform, composed of clavate terminal cells, 22–49 × 6–12 μm, thin-walled, with orange white (5A3–A3) intracellular pigments. Stipe covering a cutis consisting of cylindrical hyphae; hyphae repent, 4–13 μm wide, thin-walled, hyaline. Clamp connections present.

###### Habit and habitat.

Solitary to scattered on humus-rich grassy soil in broad-leaved forests.

###### Distribution.

Currently known only from Jilin Province, China.

###### Additional specimens examined.

CHINA • Jilin: Jiaohe City, Qianjin Forest Farm, 43°57'10"N, 127°42'7"E, alt. ca. 650 m, 24 July 2022, coll. W.N. Hou, FJAU78311.

###### Notes.

Macroscopically, *Lepiota cristatosimilis* exhibits strong affinities with the *L. cristata* complex and a suite of allied species, including *L. cristata*, *L. cristata* var. *macrospora*, *L. cristatanea*, as well as *L. paracristata*, *L. insimulata*, *L. recondita*, *L. castaneidisca*, *L. phaeoderma*, and *L. condylospora*. These taxa share a pileus densely covered with reddish-brown, dark brown, or pale orange to orange-yellow squamules, often featuring a distinct and intact central disc. However, microscopic characters provide sufficient diagnostic evidence to segregate *L. cristatosimilis* from the aforementioned species. First, while *L. cristata*, *L. cristata* var. *macrospora*, and *L. cristatanea* typically produce larger basidiomata (with *L. cristata* reaching up to 7 cm in diam.) (Yang et al. 2019), the mature pileus of *L. cristatosimilis* generally measures less than 3 cm. Furthermore, the former three taxa are non-congophilous or react only sluggishly in Congo Red, whereas *L. cristatosimilis* is distinctly congophilous. Regarding chemical reactions, the basidiospores of *L. castaneidisca* and *L. condylospora* are dextrinoid (Sysouphanthong et al. 2020a), while those of *L. cristatosimilis* are non-dextrinoid. In terms of spore morphology, *L. phaeoderma* and *L. hymenoderma* possess ellipsoid spores, whereas those of *L. cristatosimilis* are rectangular. Most crucially, although *L. condylospora* and *L. cristata* var. *macrospora* also feature nodulose spores, their dimensions (5.0–7.5 × 3.5–5.5 µm and 6.0–8.0 × 3.0–4.0 µm, respectively) are significantly broader than those of *L. cristatosimilis* (5.4–6.6 × 2.2–3.1 μm), with markedly different Q-value ranges.

##### Lepiota

Taxon classificationAnimaliaAgaricalesAgaricaceae

sect. Eriophorae (Bon) Reschke & Sarawi, in Sarawi, Piepenbring & Reschke, Fungal Syst. Evol. 15: 243

62506F28-02C3-5312-9EEF-0B309A8C489E

###### Type species.

*Lepiota eriophora* Peck.

###### Notes.

Based on our phylogenetic analysis (Figs 2, 3), the species previously transferred to *Cystolepiota* as *C. squamulosa* is shown to nest securely within *Lepiota* sect. *Eriophorae*. Consequently, this study formally reinstates its original taxonomic combination, *Lepiota squamulosa*.

##### Lepiota squamulosa

Taxon classificationAnimaliaAgaricalesAgaricaceae

T. Bau & Yu Li, J. Fungal Res. 2(3): 49 (2004)

79730704-8AE5-54C3-A2A9-300761026DB1

542274

###### Homotypic synonym.

*Cystolepiota squamulosa* (T. Bau & Yu Li) Zhu L. Yang, Mycosystema 36(5): 545 (2017) [MycoBank No: 818787].

##### Lepiota granulosquama

Taxon classificationAnimaliaAgaricalesAgaricaceae

T. Bau & X.Y. Zhou sp. nov.

45F5678D-5134-52D7-BF35-3AAF83C3FCD9

Index Fungorum: IF905873

Figs 9d, 13a–f, 24

###### Etymology.

“*granulosquama*” (from Latin granulo-, a compounding prefix derived from granulum = granule or small grain, and squama = scale) refers to the characteristic granular squamules present on both the pileus and stipe of this species.

###### Holotype.

CHINA • Jilin: Baishan City, Lushuihe National Forest Park, 42°32'53"N, 128°0'10"E, alt. ca. 660 m, 28 August 2024, coll. J.L. Wei, FJAU79253.

###### Diagnosis.

*Lepiota granulosquama* is characterized by its small basidiomata lacking cheilocystidia, and cream to reddish-brown pileus squamules. It is morphologically most similar to *L. verruculosa*, but can be distinguished by its distinctly colored squamules and its typical occurrence in broadleaf-dominated mixed forests.

###### Description.

Basidiomata small. Pileus 1.3–2.3 cm in diam., plano-convex to applanate; surface densely covered with fine, floccose, cream squamules, superimposed by a layer of granular, cream to reddish brown (8E6–E8) squamules, which are densely packed at the center; margin occasionally with velar remnants, concolorous with the squamules. Context thin, white to cream, unchanging when bruised. Lamellae free, white to cream, crowded, with lamellulae. Stipe 3.8–5.7 × 0.2–0.4 cm, subcylindrical, slightly broadening towards the base, cream; annular zone present; nearly glabrous above the annular zone, densely covered with concolorous to pinkish white (7A2) tomentose to granular squamules below. Odor and taste not recorded.

Basidiospores [40/2/2] 3.6–4.4 × 1.9–2.6 μm, avl × avw = 4.0 × 2.3 μm, Q = 1.49–2.03, Qav = 1.75, ellipsoid to oblong, occasionally cylindrical, dextrinoid, thin-walled, smooth-walled under the LM and SEM, non-metachromatic. Basidia 14–21 × 4–7 μm, clavate, thin-walled, 4-spored, occasionally 2-spored. Cheilocystidia not observed. Pleurocystidia not observed. Pileus covering 3-layered: basal hyphae hyaline, thin-walled; intermediate elements slightly thick-walled, composed of elongate-ellipsoid to cylindrical cells arranged in long chains, 7–19 wide, with light brown intracellular pigments; terminal elements forming moniliform chains of abundant inflated cells, subglobose, obovoid to oblong, 5–43 μm, with light brown to brown intracellular pigments. Stipe covering similar to pileus covering in layer structure, cellular shape, and pigmentation; intermediate elements 5–13 μm wide; terminal elements with inflated cells slightly smaller, measuring 12–31 μm. Clamp connections present.

###### Habit and habitat.

Solitary on humus soil in broad-leaved forests.

###### Distribution.

Known from Jilin and Inner Mongolia, China.

###### Additional specimens examined.

CHINA • Jilin: Dunhua City, state-owned forest farm, 27 July 2022, coll. F. Guo, FJAU79252; Inner Mongolia: Tongliao City, Daqinggou National Nature Reserve, 42°46'38"N, 122°11'46"E, alt. ca. 240 m, 31 July 2025, coll. X.Y. Zhou and T. Bau, FJAU79254.

###### Notes.

Among the three collections of *L. granulosquama* examined in this study, specimen FJAU79254—collected from sandy soil in a broadleaf forest dominated by *Ulmus* sp., *Quercus mongolica*, and *Acer* sp.—exhibited a distinct bruising reaction: its white to cream lamellae turned deep carrot-orange upon injury. However, this phenomenon was not observed in the other two collections. Therefore, it remains unclear whether this discoloration is a stable diagnostic character for the species or an environmentally induced variation.

Within *Lepiota* sect. *Eriophorae*, *Lepiota granulosquama* belongs to a subgroup of seven species characterized by the absence of cheilocystidia, alongside *L. carinii* Bres., *L. jacobi* Vellinga & Knudsen, *L. omninoflava* Y.J. Hou & Z.W. Ge, *L. pseudoasperula* (Knudsen) Knudsen, *L. squamulose* T. Bau & Yu Li, and *L. verruculosa* T. Bau & X.Y. Zhou (Knudsen 1980; Vellinga 2001b; Bau and Li 2004; Hou and Ge 2020; Sarawi et al. 2025a). However, *L. granulosquama* can be readily distinguished from these allied taxa through a combination of morphological, ecological, and biogeographical features.

Macroscopically, *L. granulosquama* produces a relatively small pileus measuring 1.3–2.3 cm in diameter with cream to reddish-brown squamules. This coloration clearly separates it from *L. squamulosa*, which exhibits pink squamules (Bau and Li 2004), and *L. omninoflava* (Hou and Ge 2020), characterized by yellow squamules. Additionally, *L. jacobi* is easily distinguished by its significantly larger basidiomata, featuring a pileus 1.9–5.1 cm across, and slightly larger basidiospores measuring 4.1–5.1 × 2.5–2.9 μm (Vellinga 2001b; Sarawi et al. 2025a).

Biogeographically and micromorphologically, *L. granulosquama* differs from *L. carinii* and *L. pseudoasperula*. The latter two species are primarily distributed in Europe and North America, with rare occurrences in Africa and Asia. Furthermore, both possess relatively broader basidiospores, recording 4.0–4.5 × 2.5–3.1 μm for *L. carinii* and 4.0–4.8 × 2.4–2.8 μm for *L. pseudoasperula* (Knudsen 1980; Sarawi et al. 2025a).

Morphologically, *L. granulosquama* is most similar to *L. verruculosa*, as both develop small basidiomata and share similar basidiospore dimensions. However, they can be segregated by pileus coloration and habitat preferences. *L. verruculosa* features grayish-orange to light brown squamules and predominantly occurs in coniferous-dominated mixed forests. In contrast, *L. granulosquama* possesses cream to reddish-brown squamules and typically inhabits broadleaf-dominated mixed forests.

##### Lepiota huadianensis

Taxon classificationAnimaliaAgaricalesAgaricaceae

T. Bau & X.Y. Zhou sp. nov.

ABCE2F59-0575-5457-80E0-515C58ED2EE3

Index Fungorum: IF905874

Figs 9e, 13g–i, 25

###### Etymology.

“*huadianensis*” refers to Huadian City, Jilin Province, China, the type locality of this new species.

###### Holotype.

CHINA • Jilin Province: Huadian City, Hongshi National Forest Park, 42°50'01"N, 127°07'57"E, alt. ca. 490 m, 31 August 2025, Y.F. Han, FJAU79267.

###### Diagnosis.

*Lepiota huadianensis* is characterized by its grayish-orange to light brown pileus squamules and distinctly fusiform to clavate cheilocystidia. It differs from its sister taxon, *L. subhuadianensis*, primarily by having noticeably larger basidiospores (4.0–5.0 × 1.9–2.8 μm).

###### Description.

Basidiomata small. Pileus 1.7–2.2 cm in diam., plano-convex to applanate; surface densely covered with grayish orange (6B4–B8) fine, floccose squamules that are easily disrupted, superimposed by erect, pyramidal to granular, light brown (6D6–D8) squamules which are densely packed at the disc; margin occasionally with velar remnants concolorous with the squamules. Context thin, white to cream, unchanging when bruised. Lamellae free, white to cream, crowded, with 3 tiers of lamellulae; edge concolorous. Stipe 2.0–4.9 × 0.2–0.3 cm, subcylindrical, slightly broadening towards the base, cream; annular zone present, nearly glabrous above, densely covered with concolorous to grayish orange (6B4–B6) tomentose to floccose squamules below, sometimes with light brown (6D6–D8) granular squamules overlaid on the floccose layer; squamules easily detachable, exposing the reddish brown (8E5–E8) stipe background. Odor and taste not recorded.

Basidiospores [40/2/2] 4.0–5.0 × 1.9–2.8 μm, avl × avw = 4.5 × 2.3 μm, Q = 1.69–2.31, Qav = 1.95, oblong to cylindrical, dextrinoid, non-metachromatic, thin-walled, smooth under both LM and SEM. Basidia 13–17 × 4–7 μm, clavate, 4-spored, occasionally 2-spored, thin-walled. Cheilocystidia 20–32 × 4–10 μm, fusiform to clavate, thin-walled. Pleurocystidia absent. Pileus covering 3-layered: basal hyphae hyaline, thin-walled; intermediate elements slightly thick-walled, composed of elongate-ellipsoid to cylindrical cells arranged in long chains, 5–17 wide, with light brown intracellular pigments; terminal elements forming moniliform chains of abundant inflated cells, subglobose, obovoid to oblong, 9–57 μm, with light brown to brown intracellular pigments. Stipe covering similar to pileus covering in layer structure, cellular shape, and pigmentation; intermediate elements 4–11 μm wide; terminal elements with inflated cells measuring 10–32 μm. Clamp connections present.

###### Habit and habitat.

Solitary on humus-rich soil in mixed conifer-broadleaf forests dominated by broad-leaved trees.

###### Distribution.

Currently known only from Jilin Province, China.

###### Additional specimens examined.

CHINA • Jilin: Huadian City, Hongshi National Forest Park, 42°50'01"N, 127°07'57"E, alt. ca. 490 m, 30 July 2025, J.H. Zu, FJAU79268.

###### Notes.

Within *Lepiota* sect. *Eriophorae*, *L. huadianensis*, *L. scabra* T. Bau & X.Y. Zhou, and *L. subhuadianensis* T. Bau & X.Y. Zhou belong to a morphologically similar group of ten species known to possess cheilocystidia (sharing this trait with *L. boertmannii* Knudsen, *L. cylindrocystidia* Sysouph. & K.D. Hyde, *L. echinobispora* Sarawi & Reschke, *L. flavocarpa* Sysouph. & K.D. Hyde, *L. haroonabadensis* Niazi, Asif, Izhar & Khalid, *L. maerimensis* Sysouph. & K.D. Hyde, and *L. minutoflava* Sarawi) (Knudsen 1980; Tibpromma et al. 2017; Niazi et al. 2021; Sarawi et al. 2025b).

Microscopically, the shape of the cheilocystidia serves as a key diagnostic character to segregate them. *Lepiota huadianensis* produces fusiform to clavate cheilocystidia (Niazi et al. 2021), which easily separate it from *L. scabra* (clavate to broadly clavate), *L. subhuadianensis* (narrowly clavate to narrowly fusiform), and *L. cylindrocystidia* (mostly cylindrical) (Tibpromma et al. 2017). Furthermore, these species differ from *L. echinobispora*, which typically possesses 2-spored basidia (Sarawi et al. 2025b). In terms of chemical reactions, their dextrinoid basidiospores distinguish them from *L. maerimensis* and *L. boertmannii* (non-dextrinoid) as well as *L. haroonabadensis* (weakly dextrinoid) (Knudsen 1980; Tibpromma et al. 2017).

Macroscopically, *L. huadianensis* (grayish-orange to light brown), *L. scabra* (orange-gray to brownish-orange), and *L. subhuadianensis* (brownish-orange to brown) share similarly dull-colored squamules. This clearly distinguishes them from noticeably brighter species in the section, such as *L. minutoflava* (yellow to orange) and *L. flavocarpa* (yellow to vivid yellow) (Tibpromma et al. 2017; Sarawi et al. 2025b).

Phylogenetically, *L. huadianensis* is recovered as the sister taxon to *L. subhuadianensis*. Despite their macroscopic similarities, *L. huadianensis* can be reliably separated from *L. subhuadianensis* by its notably larger basidiospores (4.0–5.0 × 1.9–2.8 μm vs. 3.6–4.3 × 1.7–2.3 μm), in addition to the aforementioned differences in cheilocystidia shape.

##### Lepiota scabra

Taxon classificationAnimaliaAgaricalesAgaricaceae

T. Bau & X.Y. Zhou sp. nov.

5EA9A77B-9259-511C-ABDC-C74B6CBAAB0D

Index Fungorum: IF905875

Figs 9h, 14a–f, 26

###### Etymology.

“*scabra*” (Latin, meaning rough or scurfy) refers to the macroscopic texture of the basidiomata, characterized by the pileus and stipe being densely covered with erect, conical to granular squamules.

###### Holotype.

CHINA • Jilin: Huadian City, Hongshi National Forest Park, 42°49'43"N, 127°8'28"E, alt. ca. 530 m, coll. X.Y. Zhou, FJAU79249.

###### Diagnosis.

*Lepiota scabra* is morphologically distinguished from its closest sister species, *L. pseudoasperula* and *L. squamulosa*, by the presence of cheilocystidia (completely absent in both) and the lack of pink squamules typical of *L. squamulosa*.

###### Description.

Basidiomata small. Pileus 0.8–1.8 cm in diam., obtusely conical when young, becoming plano-convex to applanate with age; surface densely covered with fine, floccose, cream to orange gray (6B2–B3) squamules, superimposed by a layer of erect, pyramidal to granular, brownish orange (6D3–D5), which are densely packed at the center. margin occasionally with velar remnants, concolorous with the squamules. Lamellae free, white to cream, crowded, with 3 tiers of lamellulae. Stipe 2–4.9 × 0.2–0.3 cm, subcylindrical, slightly broadening towards the base, cream; annular zone present; nearly glabrous above the annular zone, densely covered with concolorous to orange gray tomentose squamules below, which are sometimes dotted with brownish orange granular squamules. Context in pileus white to cream, thin, unchanging when bruised; in stipe cream to pale brownish, hollow. Odor and taste not recorded.

Basidiospores [40/2/2] 3.5–4.5 × 2.0–2.4 μm, avl × avw = 3.9 × 2.2 μm, Q = 1.58–1.98, Qav = 1.75, oblong, dextrinoid, non-metachromatic, thin-walled, smooth under LM and SEM. Basidia 12–20 × 4–7 μm, clavate, thin-walled, 4-spored, occasionally 2-spored. Cheilocystidia 21–40 × 5–12 μm, clavate to broadly clavate, thin-walled. Pleurocystidia not observed. Pileus covering 3-layered: basal hyphae hyaline, thin-walled; intermediate elements slightly thick-walled, composed of elongate-ellipsoid to cylindrical cells arranged in long chains, 5–18 wide, with light brown intracellular pigments; terminal elements forming moniliform chains of abundant inflated cells, subglobose, obovoid to oblong, 9–57 μm, light brown to brown. Stipe covering similar to pileus covering in layer structure, cellular shape, and pigmentation; intermediate elements 5–11 μm wide; terminal elements with inflated cells measuring 13–35 μm diam. Clamp connections present in all parts.

###### Habit and habitat.

Solitary to scattered on humus soil in mixed coniferous and broad-leaved forests, predominantly dominated by broad-leaved trees.

###### Distribution.

Currently known from Jilin and Liaoning Provinces, China.

###### Additional specimens examined.

CHINA • Jilin: Baishan City, Fusong county, 28 July 2022, coll. F. Guo, FJAU79249; 28 August 2022, coll. X. Wang, FJAU79251; Liaoning: Benxi city, Tuotanggou Greenstone Valley National Forest Park, 41°4'8"N, 124°12'59"E, alt. ca. 560 m, 8 July 2025, coll. X.Y. Zhou, FJAU79250.

###### Notes.

Morphologically, *Lepiota scabra* is highly similar to *L. huadianensis* and *L. subhuadianensis*, sharing dull-colored pileus squamules and distinctly dextrinoid spores. However, it can be differentiated by its clavate to broadly clavate cheilocystidia. For a comprehensive morphological comparison with other cheilocystidia-bearing species in sect. *Eriophorae*, see the taxonomic notes under *L. huadianensis*.

Phylogenetically, *L. scabra* is most closely related to *L. squamulosa*. However, they are easily separated morphologically, as *L. squamulosa* exhibits bright pink pileus squamules, contrasting sharply with the orange-gray to brownish-orange squamules of *L. scabra*.

##### Lepiota subhuadianensis

Taxon classificationAnimaliaAgaricalesAgaricaceae

T. Bau & X.Y. Zhou sp. nov.

4371BE0B-A99F-5ACC-8FD6-DECFE84B85CA

Index Fungorum: IF906077

Figs 9j, 14g–k, 27

###### Etymology.

“*subhuadianensis*” (from the Latin prefix sub-, meaning near, somewhat, or closely related to, and *huadianensis*, referring to *Lepiota huadianensis*) refers to its strong macroscopic resemblance and close phylogenetic affinity to *L. huadianensis*.

###### Holotype.

CHINA • Jilin: Huadian City, Hongshi National Forest Park, 42°50'01"N, 127°07'57"E, alt. ca. 490 m, 16 August 2024, coll. X.Y. Zhou, FJAU79264.

###### Diagnosis.

*Lepiota subhuadianensis* is characterized by its brownish-orange to brown pileus squamules and narrowly clavate to narrowly fusiform cheilocystidia. It is readily separated from its closest relative, *L. huadianensis*, by its smaller basidiospores (3.6–4.3 × 1.7–2.3 μm).

###### Description.

Basidiomata small. Pileus 1.9–2.4 cm in diam., obtusely conical when young, becoming plano-convex to applanate with age; surface densely covered with fine, floccose, orange gray (6B2–B3) to brownish orange (6C3–C5) squamules, superimposed by a layer of erect, pyramidal to granular, light brown (6E4–E6) to brown (6E5–E8) squamules, which are densely packed at the center; margin occasionally with velar remnants, concolorous with the squamules. Context white to cream, thin. Lamellae free, white to cream, crowded; lamellulae present; edge concolorous. Stipe 3.5–6.9 × 0.3–0.5 cm, subcylindrical, slightly broadening towards the base, cream; annular zone present; nearly glabrous above the annular zone, densely covered with light brown to brown tomentose to granular squamules below. Odor and taste not recorded.

Basidiospores [40/2/2] 3.6–4.3 × 1.7–2.3 μm, avl × avw = 3.9 × 2.0 μm, Q = 1.67–2.24, Qav = 1.95, oblong to cylindrical, dextrinoid, thin-walled, smooth under LM and SEM, non-metachromatic. Basidia 12–20 × 4–7 μm, clavate, thin-walled, 4-spored, occasionally 2-spored. Cheilocystidia 23–40 × 4–10 μm, narrowly clavate to narrowly fusiform, slightly thick-walled. Pleurocystidia not observed. Pileus covering 3-layered: basal hyphae hyaline, thin-walled; intermediate elements slightly thick-walled, composed of elongate-ellipsoid to cylindrical cells arranged in long chains, 5–18 μm wide, with light brown intracellular pigments; terminal elements forming moniliform chains of abundant inflated cells, subglobose, obovoid to oblong, 8–45 μm, with brown intracellular pigments. Stipe covering similar to pileus covering in layer structure, cellular shape, and pigmentation; intermediate elements 5–18 μm wide; terminal elements with inflated cells slightly smaller, measuring 9–38 μm. Clamp connections present.

###### Habit and habitat.

Solitary to scattered on humus soil in broad-leaved forests.

###### Distribution.

Currently known only from Jilin Province, China.

###### Additional specimens examined.

CHINA • Jilin: Helong City, Xianfeng Forest Park, 22 August 2022, coll. T. Bau, FJAU79266; Baishan City, Lushuihe National Forest Park, 42°29'56"N, 128°0'17"E, alt. ca. 780 m, 8 August 2024, coll. H. Cheng and Y. Wang, FJAU79263, FJAU79265.

###### Notes.

*Lepiota subhuadianensis* is the phylogenetic sister taxon to *L. huadianensis*. While they are macroscopically difficult to separate, *L. subhuadianensis* is primarily characterized by its smaller basidiospores (3.6–4.3 × 1.7–2.3 μm) and narrowly clavate to narrowly fusiform cheilocystidia. For a detailed discussion and morphological comparison with other allied species in sect. *Eriophorae*, please refer to the notes under *L. huadianensis*.

##### Lepiota verruculosa

Taxon classificationAnimaliaAgaricalesAgaricaceae

T. Bau & X.Y. Zhou sp. nov.

08AF3C32-0D28-533E-82B1-1EB7091FF239

Index Fungorum: IF905878

Figs 9k, 15a–f, 28

###### Etymology.

“*verruculosa*” (from Latin verruculosus, meaning finely verrucose or warty) refers to the prominent verrucose squamules densely covering both the pileus and the lower part of the stipe.

###### Holotype.

CHINA • Liaoning: Benxi city, Tuotanggou Greenstone Valley National Forest Park, 41°4'8"N, 124°12'59"E, alt. ca. 560 m, 8 July 2025, coll. X.Y. Zhou, FJAU79261.

###### Diagnosis.

*Lepiota verruculosa* is characterized by its small basidiomata lacking cheilocystidia, and grayish-orange to light brown pileus squamules. It differs from its closest morphological counterpart, *L. granulosquama*, primarily by its squamule coloration and its preference for coniferous-dominated mixed forests.

###### Description.

Basidiomata small. Pileus 0.3–2.0 cm in diam., hemispherical when young, becoming plano-convex to applanate with age; surface densely covered with fine, floccose, cream to grayish orange (6B2–B3) squamules; superimposed by a layer of erect, verrucose to granular, light brown (6D5–D8) squamules, which are densely packed at the center; margin occasionally with velar remnants, concolorous with the squamules. Context white, thin. Lamellae free, white to cream, crowded; lamellulae present; edge concolorous. Stipe 1.8–5.0 × 0.2–0.4 cm, subcylindrical, slightly broadening towards the base, cream; annular zone present; nearly glabrous above the annular zone, densely covered with grayish orange to light brown tomentose to verrucose squamules below. Odor and taste not recorded.

Basidiospores [60/3/3] 3.7–4.5 × 2.0–2.6 μm, avl × avw = 4.0 × 2.3 μm, Q = 1.49–2.06, Qav = 1.76, ellipsoid to oblong, occasionally cylindrical, dextrinoid, non-metachromatic, thin-walled, smooth under LM and SEM. Basidia 11–16 × 4–7 μm, clavate, thin-walled, 4-spored, occasionally 2-spored. Cheilocystidia not observed. Pleurocystidia not observed. Pileus covering 3-layered: basal hyphae hyaline, thin-walled; intermediate elements slightly thick-walled, composed of elongate-ellipsoid to cylindrical cells arranged in long chains, 9–18 μm wide, with light brown intracellular pigments; terminal elements forming moniliform chains of abundant inflated cells, subglobose, obovoid to oblong, 11–41 μm, with light brown to brown intracellular pigments. Stipe covering similar to pileus covering in layer structure, cellular shape, and pigmentation; intermediate elements 5–17 μm wide; terminal elements with inflated cells measuring 11–35 μm. Clamp connections present.

###### Habit and habitat.

Solitary to scattered on humus soil in coniferous or mixed coniferous and broad-leaved forests.

###### Distribution.

Currently known from Heilongjiang, Jilin, and Liaoning Provinces, China.

###### Additional specimens examined.

CHINA • Jilin: Jiaohe City, Qianjin Forest Farm, 43°57'10"N, 127°42'7"E, alt. ca. 650 m, 23 July 2022, coll. L.S. Mu, FJAU79257, FJAU79258; 24 July 2022, coll. X. Wang and W.N. Hou FJAU79255, FJAU79262; Baishan City, Lushuihe National Forest Park, 42°32'53"N, 128°0'10"E, alt. ca. 660 m, 8 August 2024, Q.R. Liu, FJAU79256; Heilongjiang: Daxinganling area, Guqi Valley National Wetland Park, 52°19'53"N, 124°43'10"E, alt. ca. 350 m, 14 August 2025, coll. X.Y. Zhou, FJAU79259, FJAU79260.

###### Notes.

Among the eight collections of *L. verruculosa* examined in this study, the two basidiomata of specimen FJAU79259—collected from the humus layer in a coniferous forest dominated by *Larix* sp., *Pinus sylvestris* var. mongolica, and *Picea* sp.—exhibited a distinct bruising reaction: their white to cream lamellae turned copper upon injury. However, this phenomenon was absent in the remaining seven collections. Therefore, it remains unclear whether this discoloration represents a stable diagnostic character or merely an environmentally induced variation.

Within *Lepiota* sect. *Eriophorae*, *Lepiota verruculosa* belongs to the morphological subgroup characterized by the absence of cheilocystidia. Morphologically, it is most likely to be confused with *L. granulosquama*, as both species produce small basidiomata and share highly similar basidiospore dimensions. However, *L. verruculosa* can be readily distinguished by its grayish-orange to light brown pileus squamules and its distinct ecological preference for coniferous-dominated mixed forests. For a comprehensive morphological and biogeographical comparison with other cheilocystidia-lacking species in this section, please refer to the taxonomic notes under *L. granulosquama*.

##### Lepiota

Taxon classificationAnimaliaAgaricalesAgaricaceae

sect. Eriophorae subsect. Pseudocystolepiotae T. Bau & X.Y. Zhou subsect. nov.

62B50DA8-0D05-504F-AC55-EBA49812F366

865351

###### Etymology.

The subsectional epithet refers to the *Cystolepiota*-like macroscopic appearance of the representative species.

###### Type species.

*Lepiota fugaciosquamosa* T. Bau & X.Y. Zhou.

###### Diagnosis.

Pileus covered with *Cystolepiota*-like powdery and floccose-mealy squamules, or *Eriophorae*-like fine, acute squamules; pileus covering 2- or 3-layered. Annulus indistinct. Basidiomata typically unchanging upon drying, though discoloration occurs in *L. fugaciosquamosa*. Basidiospores dextrinoid or non-dextrinoid. Cheilocystidia present. Clamp connections present.

###### Notes.

Phylogenetically, this novel subsection is introduced here to accommodate an early-diverging lineage (96/0.93) comprising *Lepiota fugaciosquamosa*, *L. icterina* (formerly *Cystolepiota icterina*), and *L. boertmannii*. This clade is recovered as the sister group to the core clade of *Lepiota* sect. *Eriophorae* (95/0.93).

Currently, this subsection encompasses three species. Morphologically, *L. fugaciosquamosa* and *L. icterina* exhibit typical *Cystolepiota*-like pileal features, characterized by powdery, floccose-mealy squamules and a 2-layered pileus covering. Although *L. icterina* was previously placed in *Cystolepiota* sect. *Pseudoamyloideae* (Knudsen 1978), phylogenetic evidence firmly nests it within *Lepiota*. Retaining these taxa in *Cystolepiota* would severely complicate generic boundaries and render the genus polyphyletic. Furthermore, the inclusion of *L. boertmannii*—an independent, long-branch lineage formerly placed at the base of sect. *Eriophorae* that features fine, acute squamules and a 3-layered pileus covering—highlights the highly transitional nature of this clade (Kimura 1980). Therefore, subsect. *Pseudocystolepiotae* is formally established within sect. *Eriophorae* to accommodate these taxa, which represent a morphological and evolutionary bridge between *Cystolepiota* and the core *Lepiota* sect. *Eriophorae*.

##### Lepiota icterina

Taxon classificationAnimaliaAgaricalesAgaricaceae

(F.H. Møller ex Knudsen) T. Bau comb. nov.

6386ABB8-11A0-55BB-B319-D1ACBC8B1F4C

865519

###### Basionym.

*Cystolepiota icterina* F.H. Møller ex Knudsen, Bot. Tidsskr. 73(2): 130 (1978) [MycoBank No: 312550].

###### Synonym.

*Lepiota icterina* F.H. Møller, Friesia 7(3): 453 (1965) [nom. inval., Art. 39.1, 40.1] [MycoBank No: 477259].

##### Lepiota fugaciosquamosa

Taxon classificationAnimaliaAgaricalesAgaricaceae

T. Bau & X.Y. Zhou sp. nov.

87181D3B-1586-5A48-8C7A-141BB1728724

Index Fungorum: IF906078

Figs 9l, 16e, f, 29

###### Etymology.

“*fugaciosquamosa*” (from Latin fugax, fleeting, and squamous, squamous), refers to the delicate, pulverulent squamules on the pileus and stipe that are easily rubbed off or lost during development and handling.

###### Holotype.

CHINA • Jilin: Baishan City, Jingyu National Nature Reserve; 29 July 2022, coll. L.S. Mu, FJAU79282.

###### Diagnosis.

*Lepiota fugaciosquamosa* is characterized by its white pileus squamules, non-dextrinoid basidiospores, and a distinct discoloration of the basidiomata upon drying. It is readily distinguished from its phylogenetic sister taxon, *L. icterina*, which produces sulfur-yellow to yellowish-brown squamules, possesses pseudamyloid spores, and lacks such drying-induced discoloration.

###### Description.

Basidiomata small. Pileus 0.9–2.5 cm in diam., obtusely conical when young, becoming plano-convex with age; surface white, densely covered with concolorous pulverulent to granular squamules that are easily deciduous; center white to cream; margin occasionally with velar remnants, concolorous with the squamules. Context white, thin. Lamellae free, white to cream, crowded; lamellulae present; edge concolorous. Stipe 2.9–5.2 × 0.3–0.4 cm, subcylindrical, slightly broadening towards the base, white; annular zone present; nearly glabrous above the annular zone, densely covered with concolorous pulverulent squamules below; stipe context hollow, fibrous, near the base reddish brown (8E5–E8). Upon drying, the pileus and stipe turn light orange (5A4–A6), and the lamellae turn light brown (6D4–D6). Odor and taste not recorded.

Basidiospores [40/2/2] 4.3–5.3 × 2.1–2.9 μm, avl × avw = 4.7 × 2.4 μm, Q = 1.59–2.31, Qav = 1.96, oblong to cylindrical, non-dextrinoid, metachromatic, thin-walled, smooth-walled under the LM and SEM. Basidia 15–22 × 4–8 μm, clavate, thin-walled, 4-spored, occasionally 2-spored. Cheilocystidia 26–53 × 5–13 μm, narrowly clavate to narrowly fusiform, slightly thick-walled. Pleurocystidia not observed. Pileus covering 2-layered: basal hyphae hyaline, thin-walled; apical layer composed of sparsely arranged hyphae and abundant inflated cells; inflated cells subglobose, obovoid, oblong to sphaeropedunculate, sometimes 2–5 connected in short moniliform chains, easily detachable, 12–58 μm, hyaline. Stipe covering similar to pileus covering in layer structure, cellular shape, and absence of pigmentation. Clamp connections present.

###### Habit and habitat.

Scattered on humus soil in mixed coniferous and broad-leaved forests.

###### Distribution.

Currently known only from Jilin Province, China.

###### Additional specimens examined.

CHINA • Jilin: Helong City, Xianfeng Forest Park, 22 August 2020, coll. T. Bau, FJAU79281.

###### Notes.

Macroscopically, *Lepiota fugaciosquamosa* strongly resembles several white-basidiomata species within *Cystolepiota*, such as *C. hongshiensis* T. Bau & X.Y. Zhou, *C. nivalis* T. Bau & X.Y. Zhou, and the well-known *C. seminuda* (Lasch) Bon (Qu et al. 2023; Zhou and Bau 2024). However, it can be easily distinguished in the fungarium: while these *Cystolepiota* species remain unchanged upon drying, *L. fugaciosquamosa* exhibits a distinct color shift, with its white pileus and stipe turning pale orange, and its white lamellae becoming pale brown.

Furthermore, while some whitish *Cystolepiota* taxa-namely *C. changbaishanensis* T. Bau & X.Y. Zhou and *C. mastoidea*—also undergo orange-brown or brown discoloration upon drying (Zhou and Bau 2024), they can be reliably separated from the new species by micromorphological features. Specifically, *L. fugaciosquamosa* produces smooth basidiospores under SEM and cheilocystidia lacking apical appendages. In contrast, both *C. changbaishanensis* and *C. mastoidea* feature distinctly ornamented spores and appendiculate cheilocystidia (Zhou and Bau 2024).

Phylogenetically, *L. fugaciosquamosa* is recovered as the sister taxon to *L. icterina*. Despite their close genetic affinity, they are morphologically distinct. As noted in the diagnosis, *L. icterina* is characterized by its sulfur-yellow to yellowish-brown squamules and pseudamyloid basidiospores (Knudsen 1978), contrasting sharply with the white squamules, non-dextrinoid spores, and characteristic drying discoloration of *L. fugaciosquamosa*.

##### Lepiota

Taxon classificationAnimaliaAgaricalesAgaricaceae

sect. Fuscovinaceae Bon & Candusso, In: Candusso & Lanzoni, Fungi Eur. 4: 296

28A75B80-D2DF-5D84-B423-7A88EB241959

###### Type species.

*Lepiota fuscovinacea* F.H. Møller & J.E. Lange.

##### Lepiota spinulosa

Taxon classificationAnimaliaAgaricalesAgaricaceae

T. Bau & X.Y. Zhou sp. nov.

B68B7CFB-13E7-5595-82C4-C73ED1AC5708

Index Fungorum: IF905879

Figs 17, 30

###### Etymology.

“*spinulosa*” refers to the characteristic erect, spinulose squamules on the pileus.

###### Holotype.

CHINA • Jilin: Huadian City, Hongshi National Forest Park, 42°50'01"N, 127°07'57"E, alt. ca. 490 m, 16 August 2024, coll. X.Y. Zhou, FJAU78317.

###### Diagnosis.

*Lepiota spinulosa* is primarily distinguished by its spinose to granular pileus squamules formed by trichodermial, multi-septate hyphae, a feature that easily separates it from allied species possessing globose pileal cells. It differs from the closely related *L. fuscovinacea* mainly by lacking vinaceous pileus tones and possessing significantly larger basidiospores.

###### Description.

Basidiomata small. Pileus 0.7–2 cm in diam., campanulate when young, becoming plano-convex to applanate with age; surface white to cream, densely covered with variously colored tomentose to floccose squamules, cream, orange-white (6A2–A3), light brown (6D4–D6, 7D5–D8), brown (6E5–E8, 7E6–E8), dark brown (6F6–F8), pinkish white (8A2), gray (6C1, D1, E1), reddish gray (7B2–B3), and grayish orange (6B2–B4); surface superimposed by concolorous or darker, erect, spinulose or granular scale elements on the floccose squamules; margin with velar remnants, concolorous with the pileal squamules. Context thin, white to cream, unchanging when bruised. Lamellae free, white to cream, crowded; lamellulae in 2–3 tiers; edge concolorous. Stipe 2–5 × 0.2–0.3 cm, cylindrical, white to cream; nearly glabrous above the annular zone, covered with appressed or slightly reflexed, tomentose to floccose squamules concolorous with the pileal squamules below, with concolorous or darker, spinulose or granular elements on the floccose squamules. Annulus not observed. Odor and taste not recorded.

Basidiospores [60/3/3] 5.0–6.6 × 2.3–3.6 μm, avl × avw = 5.6 × 2.9, Q = 1.59–2.37, Qav = 1.97, oblong to cylindrical, hyaline, thin-walled, non-dextrinoid to weakly dextrinoid, non-metachromatic. Basidia 14–23 × 4–7 μm, clavate, 4-spored, occasionally 2-spored, thin-walled. Cheilocystidia 23–39 × 7–14 μm, clavate to broadly clavate or oblong, occasionally with fine crystals at the apex, slightly thick-walled. Pileus covering 3-layered: basal hyphae hyaline, thin-walled; intermediate hyphae slightly thick-walled, 4–15 μm wide, with light brown intracellular and parietal pigments; terminal elements forming a trichoderm, septate without clamp connections, 122–392 × 12–25 μm, containing brown intracellular pigments. Stipe covering similar to pileus covering in layer structure, cellular shape, and pigmentation; intermediate hyphae 3–15 μm wide; terminal elements forming a trichoderm with cells measuring 88–252 × 12–21 μm. Clamp connections not observed.

###### Habit and habitat.

Solitary to scattered on the humus layer or soil in mixed coniferous and broad-leaved forests.

###### Distribution.

Currently known only from Jilin Province, China.

###### Additional specimens examined.

CHINA • Jilin Province, Jiaohe City, Qianjin Forest Farm, 43°57'10"N, 127°42'7"E, alt. ca. 650 m, 23 July 2023, coll. H.B. Song and T. Bau, FIAU78322, FJAU78316; 25 August 2023, coll. M. Liu, H. Chenh and T. Bau, FJAU78315; FJAU78321; Tonghua City, Baijifeng National Forest Park, 41°33'38"N, 126°4'19"E, alt. ca. 840 m, 8 July 2023, coll. Q.R. Liu, FJAU78314; 14 July 2024, coll. J.L. Wei, FJAU78318; Huadian City, Hongshi National Forest Park, 42°50'01"N, 127°07'57"E, alt. ca. 490 m, 28 August 2023, coll. S.Y. Li and T. Bau, FJAU7832; 16 September 2025, coll. Y.F. Han, FJAU78323; Baishan City, Songjiang River National Forest Park, 42°10'32"N, 127°30'37"E, alt. ca. 760 m, 14 July 2024, coll. Q.R. Liu, FJAU78319; Baishan City, Lushuihe National Forest Park, 42°32'53"N, 128°0'10"E, alt. ca. 660 m, 8 August 2024, coll. Y. Wang, FJAU78320.

###### Notes.

Macroscopically, *Lepiota spinulosa* possesses spinose to granular squamules, making it highly similar to species within *Lepiota* sect. *Fuscovinaceae*, sect. *Eriophorae*, and *Echinoderma*. However, the uppermost layer of the pileal squamules in this species is composed of trichodermial, multi-septate hyphae. This crucial microscopic feature clearly distinguishes it from members of sect. *Eriophorae* and *Echinoderma*; although the latter two groups also exhibit spinose to granular squamules macroscopically, their uppermost pileal squamules are fundamentally different, being composed of globose to sphaeropedunculate cells.

Furthermore, *L. spinulosa* is macroscopically and phylogenetically closely related to *L. fuscovinacea*. Nevertheless, *L. fuscovinacea* can be separated by its pinkish, vinaceous to pale brown pileal squamules, along with its significantly smaller basidiospores measuring 4.5–6 × 2–2.5 μm (Yang et al. 2019).

##### Lepiota

Taxon classificationAnimaliaAgaricalesAgaricaceae

sect. Lepiota (Pers.) Gray, Nat. Arr. Brit. Pl. (London) 1: 601

DC592C6B-97FD-523B-8B64-B87EB88888BD

###### Type species

**(typ. cons.)**. *Agaricus colubrinus* Pers. (= *Lepiota clypeolaria* (Bull.) P. Kumm).

##### Lepiota basozonata

Taxon classificationAnimaliaAgaricalesAgaricaceae

T. Bau & X.Y. Zhou sp. nov.

F9DBCF30-AD7A-54BC-B05D-1D0E1C616599

Index Fungorum: IF905880

Figs 18a–f, 31

###### Etymology.

“*basozonata*” (from Latin baso-, a compounding prefix derived from Greek basis = base, and Latin zonata = zonate or banded) refers to the discontinuous concentric bands of coffee brown squamules near the base of the stipe.

###### Holotype.

CHINA • Jilin: Huadian City, Hongshi National Forest Park, 42°50'01"N, 127°07'57"E, alt. ca. 490 m, 28 August 2023, coll. X.Y. Zhou and T. Bau, FJAU79181.

###### Diagnosis.

*Lepiota basozonata* features distinctive coffee brown squamules, concentric girdles on the lower stipe, and a dark-margined annulus. It differs from allied taxa possessing similar stipe girdles—namely *L. felina*, *L. pseudolilacea*, and *L. helveola*—primarily by having noticeably smaller basidiospores (5.7–6.9 × 3.0–4.0) and completely lacking pinkish, lilacinous, or flesh-red pileal tones.

###### Description.

Basidiomata small. Pileus 0.5–2.2 cm in diam., ovoid when young, becoming plano-convex to applanate with age, with an indistinct obtuse umbo; surface white; central squamules intact, brown (6E6–E8), gradually tearing into small, patch-like squamules towards the margin; marginal squamules somewhat arranged in concentric rings, light brown (6E4–E7); margin sometimes with white velar remnants. Context white, thin. Lamellae free, white to cream, crowded; lamellulae in 2–3 tiers; edge concolorous. Stipe 1.8–4.1 × 0.2–0.4 cm, subcylindrical, slightly broadening towards the base, white to cream, nearly glabrous, often with discontinuous concentric bands of light brown (6E4–E6) squamules near the base. Annulus median to superior, membranous, white, lower surface with a pastel light brown encircling margin. Odor and taste not recorded.

Basidiospores [40/2/2] 5.7–6.9 × 3.0–4.0 μm, avl × avw = 6.2 × 3.6 μm, Q = 1.59–2.06, Qav = 1.74, oblong, occasionally ellipsoid or cylindrical, hyaline, thin-walled, dextrinoid, non-metachromatic. Basidia 16–26 × 7–10 μm, clavate, 4-spored, occasionally 2-spored, thin-walled. Cheilocystidia 22–34 × 5–10 μm, clavate to fusiform, thin-walled. Pleurocystidia not observed. Pileus covering composed of a trichoderm: terminal cells 67–282 × 6–13 μm, subcylindrical to narrowly clavate, slightly acute at the apex, with light brown intracellular pigments, completely lacking parietal pigments; basal layer with short clavate elements, slightly thick-walled. Stipe covering similar to pileus covering in cellular shape and pigmentation, but structured as a shorter trichoderm; terminal cells 40–181 × 6–10 μm, subcylindrical, slightly acute at the apex. Clamp connections present.

###### Habit and habitat.

Solitary on the humus layer in mixed coniferous and broad-leaved forests.

###### Distribution.

Currently known only from Jilin Province, China.

###### Additional specimens examined.

CHINA • Jilin: Huadian City, Hongshi National Forest Park, 42°50'01"N, 127°07'57"E, alt. ca. 490 m, 31 July 2025, coll. Y.F. Han, FJAU79183; 15 August 2025, coll. W. Sun and Y.F. Han, FJAU79186, FJAU79187; 16 August 2025, coll. Y. Wang, FJAU79182; Jilin City, Zhuque Mountain, 43°46'40"N, 126°40'59"E, alt. ca. 280 m, coll. Y.F. Han and T.Y. Zhang, FJAU79184, FJAU79185.

###### Notes.

Macroscopically, *Lepiota basozonata* is highly similar to *L. felina* (Pers.) P. Karst., *L. pseudolilacea* Huijsman, and *L. helveola* Bres. These four species are all typically characterized by the presence of concentric girdles of squamules on the lower stipe and an annulus decorated with dark-colored margins or patches.

However, *L. felina* can be easily distinguished from the new species by its significantly larger and wider basidiospores (6.0–8.5 × 4.0–5.0 μm), cheilocystidia that often possess an acuminate apex and septa, and a narrow annulus tightly clasping the stipe (Vellinga 2001c).

*Lepiota pseudolilacea* differs from *L. basozonata* by having much longer and wider basidiospores measuring 6.0–10.0 × 4.0–5.0 μm. Macroscopically, its pileus usually exhibits pinkish gray-brown, dark red-brown, or even weak lilacinous tinges, which is distinctly different from the coffee brown squamules of *L. basozonata* (Vellinga 2001c).

Furthermore, *L. helveola* possesses distinctly larger and wider basidiospores (6.5–9.5 × 5.5–6.5 μm). It can be further separated from *L. basozonata* by its flesh-red, pinkish-brown to brown pileal squamules, and flesh-red to pinkish-brown squamules below the annulus, in contrast to the coffee brown squamules of the new species (Yang et al. 2019).

##### Lepiota jianensis

Taxon classificationAnimaliaAgaricalesAgaricaceae

T. Bau & X.Y. Zhou sp. nov.

2FFF5D98-8536-50CD-8A96-88A310956863

Index Fungorum: IF906079

Figs 19a–f, 32

###### Etymology.

“*jianensis*” refers to Ji’an City (Jilin Province, China), the type locality where the specimens of this new species were collected.

###### Holotype.

CHINA • Jilin: Ji’an City, Maxian Township, 7 August 2023, coll. Q.R. Liu, FJAU79175.

###### Diagnosis.

*Lepiota jianensis* features reddish brown, patch-like pileus squamules and narrowly to broadly fusiform cheilocystidia. It differs from macroscopically similar taxa primarily by lacking laterally penguin-shaped basidiospores or red-brown granular squamules. It is easily separated from its closest phylogenetic relatives, *L. umbrosa* and *L. subincarnata*, by lacking concentric stipe girdles and possessing much smaller basidiospores measuring 4.8–5.7 × 2.6–3.5 μm, entirely lacking the large 7.0–8.5 × 4.0–5.0 μm dimensions typical of *L. subincarnata*.

###### Description.

Basidiomata small. Pileus 1–2 cm in diam., hemispherical to applanate; surface white; central squamules intact, reddish brown (9D5–D8), composed of fine tomentose elements arranged in tufts, gradually tearing towards the margin into slightly reflexed, patch-like squamules; marginal squamules grayish red (8B3–B6, 9B3–B6); margin with velar remnants, concolorous with the squamules. Context white to cream, thin. Lamellae free, white to cream, crowded; lamellulae in 2–3 tiers; edge concolorous. Stipe 3–4 × 0.1–0.2 cm, subcylindrical, grayish red, densely covered with white to cream tomentose to floccose squamules; basal mycelium distinct, white to grayish red. Annulus not observed. Odor and taste not recorded.

Basidiospores [40/2/2] 4.8–5.7 × 2.6–3.5 μm, avl × avw = 5.3 × 3.1 μm, Q = 1.52–2.02, Qav = 1.71, ellipsoid to oblong, dextrinoid, thin-walled, smooth under light microscopy, non-metachromatic. Basidia 13–20 × 5–8 μm, clavate, thin-walled, 4-spored, occasionally 2-spored. Cheilocystidia 12–28 × 5–11 μm, narrowly to broadly fusiform, apex mucronate to subcapitate, slightly thick-walled, occasionally with fine apical incrustations. Pleurocystidia not observed. Pileus covering squamules composed of a trichoderm: terminal cells 106–328 × 6–12 μm, subcylindrical, slightly thick-walled, with light brown intracellular pigments; basal layer with short clavate elements, slightly thick-walled. Stipe covering similar to pileus covering in cellular shape and pigmentation, but structured as a shorter trichoderm; terminal cells 62–158 × 8–15 μm, subcylindrical to clavate. Clamp connections present.

###### Habit and habitat.

Solitary on the humus layer in broad-leaved forests.

###### Distribution.

Currently known only from the type locality (Maxian Township, Ji’an City, Jilin Province, China).

###### Additional specimens examined.

CHINA • Jilin: Ji’an City, Maxian Township, 7 August 2023, coll. Q.R. Liu, FJAU79174.

###### Notes.

Macroscopically, *Lepiota jianensis* resembles *L. rostratospora* T. Bau & X.Y. Zhou, *L. attenuata* Jun F. Liang & Zhu L. Yang, and *L. metulispora* (Berk. & Broome) Sacc. However, these three species can be readily distinguished from the new species by their laterally penguin-shaped basidiospores (Liang et al. 2011; Yang et al. 2019).

Furthermore, *L. jianensis* is morphologically comparable to the *Lepiota echinella* complex. Nevertheless, the latter differs in having red-brown, granular pileal squamules and lageniform to utriform cheilocystidia, which contrasts starkly with the apricot brown, patch-like squamules and narrowly to broadly fusiform cheilocystidia of *L. jianensis* (Vellinga 2001c).

Phylogenetically, *L. jianensis* clusters in a single clade with *L. umbrosa* and *L. subincarnata*. However, *L. umbrosa* Morgan can be separated by its predominantly yellowish-brown squamules and slightly larger basidiospores (5–6 × 3 μm) (Morgan 1906). Meanwhile, *L. subincarnata* (a member of *L.* sect. *Helveolae*) is fundamentally distinguished from the new species by its pileus covering structure. The pileus covering in sect. *Helveolae* features a trichoderm consisting exclusively of elongated cells and lacking basal clavate cells. In contrast, the pileus covering of *L. jianensis* (sect. *Lepiota*) possesses distinct basal clavate cells. Additionally, *L. subincarnata* differs macromorphologically by having a white to pinkish stipe decorated with concentric girdles of squamules, and possesses significantly larger basidiospores (7.0–8.5 × 4.0–5.0 μm) (Yang et al. 2019; Sarawi et al. 2025a).

##### Lepiota rostratospora

Taxon classificationAnimaliaAgaricalesAgaricaceae

T. Bau & X.Y. Zhou sp. nov.

30EAA778-79B8-5DDD-B7E1-02EA505609EC

Index Fungorum: IF905884

Figs 20a–f, 33

###### Etymology.

The specific epithet “*rostratospora*” (from Latin rostrato-, a compounding prefix derived from rostratus = beaked, and spora = spore) refers to the laterally penguin-shaped basidiospores with a distinctively constricted, beak-like apex.

###### Holotype.

CHINA • Jilin: Ji’an City, Yushan Park, 41°8'5"N, 126°11'2"E, alt. ca. 270 m, 2 August 2023, coll. Q.R. Liu, FJAU79178.

###### Diagnosis.

*Lepiota rostratospora* differs from its closest phylogenetic relative, *L. spheniscispora*, by possessing cheilocystidia with a distinctly constricted apex and narrower basidiospores measuring 14.1–16.1 × 3.6–4.6, lacking the wider 13–18.5 × 4.5–5.5 μm dimensions typical of the latter.

###### Description.

Basidiomata small to medium-sized. Pileus 1.2–4.2 cm in diam., plano-convex to applanate when mature; surface white; central squamules intact, reddish orange (7B7–B8), gradually tearing into small patch-like to punctate squamules towards the margin; marginal squamules pastel red (7A4–A6) to grayish red (7B4–B6); margin with velar remnants, occasionally with indistinct short striations. Context white, thin. Lamellae free, white to cream, crowded; lamellulae present; edge concolorous. Stipe 2–5 × 0.2–0.4 cm, subcylindrical, cream, with an annular zone; nearly glabrous above the annular zone, densely covered with concolorous tomentose squamules below, which are sometimes dotted with pastel red finely punctate squamules; with conspicuous white mycelium at the base. Context in stipe white to cream, near the base turning light brown (7D4–D8) to brown (7E6–E8), hollow. Odor and taste not recorded.

Basidiospores [40/2/2] (13.7–)14.1–16.1(–17.5) × 3.6–4.6 μm, avl × avw = 15.1 × 4.1 μm, Q = (3.12–)3.57–3.83(–4.41), Qav = 3.67, penguin-shaped in lateral view, with a suprahilar depression, adaxial face ventricose, abaxial face nearly straight, apex distinctly constricted and narrowed, subfusiform in frontal view, slightly thick-walled, dextrinoid, non-metachromatic. Basidia 19–30 × 9–12 μm, clavate, thin-walled, 4-spored, occasionally 2-spored. Cheilocystidia 13–37 × 8–20 μm, fusiform to clavate, mucronate to subcapitate at the apex, slightly thick-walled. Pleurocystidia not observed. Pileus covering squamules composed of a trichoderm: terminal cells 75–382 × 5–13 μm, subcylindrical, sometimes constricted at the apex, slightly thick-walled, with light brown intracellular pigments; basal layer with short clavate elements. Stipe covering similar to pileus covering in cellular shape and pigmentation, but structured as a shorter trichoderm; terminal cells 45–102 × 6–15 μm, subcylindrical. Clamp connections present.

###### Habit and habitat.

Solitary to scattered on the humus layer in mixed coniferous and broad-leaved forests.

###### Distribution.

Currently known only from Jilin Province, China.

###### Additional specimens examined.

CHINA • Jilin: Ji’an City, Mirror groove, 8 August 2023, coll. Q.R. Liu, FJAU79178; FJAU79177; Changchun City, Jingyuetan National Forest Park, 43°46'1"N, 125°29'19"E, alt. ca. 235 m, 19 August 2023, coll. J.L. Wei, FJAU79180; Huadian City, Huaxi Valley, 42°55'51.85"N, 127°6'2.63"E, alt. ca. 360 m, 15 August 2024, coll. X.Y. Zhou, FJAU79179.

###### Notes.

Macroscopically, *L. rostratospora* resembles *Lepiota attenuata* and *L. metulispora*, as all three species share a pileus with pale, fine squamules and a striate margin. However, *L. attenuata* produces a larger pileus (3–6 cm in diam.) and is further characterized by pileal squamules with basal elements composed of subglobose to broadly ellipsoid swollen cells arranged in chains (Liang et al. 2011). On the other hand, *L. metulispora* can be distinguished by its clavate to fusiform cheilocystidia, which lack the distinctly constricted, mucronate to subcapitate apices observed in the new species (Yang et al. 2019).

Phylogenetically, *L. rostratospora* is recovered as the sister taxon to *Lepiota spheniscispora* Vellinga (Vellinga 2001e), a species originally described from California, USA. Nevertheless, *L. spheniscispora* differs in having wider basidiospores (13–18.5 × 4.5–5.5 μm) and cheilocystidia lacking a constricted apex.

##### Lepiota subforquignonii

Taxon classificationAnimaliaAgaricalesAgaricaceae

T. Bau & X.Y. Zhou sp. nov.

B91DAC0F-E40E-52E1-82EE-3A69CC6058E9

Index Fungorum: IF905885

Figs 20g–l, 34

###### Etymology.

“*subforquignonii*” was chosen to reflect its close phylogenetic affinity and morphological resemblance to *L. forquignonii*.

###### Holotype.

CHINA • Jilin: Jilin City, Zhuque Mountain, 43°46'40"N, 126°40'59"E, alt. ca. 280 m, 19 July 2023, coll. S.E. Wang, FJAU79190.

###### Diagnosis.

*Lepiota subforquignonii* differs from its sister taxon, *L. forquignonii*, by possessing pastel brown to garnet red pileus squamules devoid of greenish tinges, alongside distinctively septate and branched cheilocystidia. Furthermore, it produces comparatively broader basidiospores with a lower average Q value of 1.75, lacking the narrower profiles and higher 1.8–1.9 average Q value characteristic of the latter species.

###### Description.

Basidiomata small to medium-sized. Pileus 1.5–4.0 cm in diam., initially obtuse-conical, becoming plano-convex to applanate; surface cream, densely covered with cream to brownish gray (6C2) fine squamules; squamules occasionally diffracted, exposing the white context; disc squamules reddish brown (7E7–E8) to brown (6E6–E8), intact, not diffracted; margin with white or concolorous veil remnants. Context white, thin. Lamellae free, white to cream, crowded; lamellulae of various lengths present; edge concolorous. Stipe 3.0–7.0 cm long, 0.2–0.4 cm thick, subcylindrical, slightly broadening towards the base, white to cream; annulus median to superior, white, fibrillose-membranous, fugacious; surface nearly glabrous above the annulus; covered with white villose to floccose squamules below; floccose squamules occasionally interspersed with fine punctate squamules concolorous with the pileus. Odor and taste not recorded.

Basidiospores [80/4/4] 5.7–7.6(–8.2) × 3.0–4.0(–4.4) μm, avl × avw = 6.4 × 3.7 μm, Q = 1.50–2.10(–2.24), Qav = 1.75, ellipsoid to cylindrical, hyaline, slightly thick-walled, dextrinoid, non-metachromatic. Basidia 16–24 × 5–7 μm, clavate, thin-walled, 4-spored, occasionally 2-spored. Cheilocystidia 15–30 × 4–7 μm, clavate, septate, occasionally branched, thin-walled. Pleurocystidia not observed. Pileus covering composed of a trichoderm: terminal cells 145–367 × 6–12 μm, subcylindrical, with yellowish white (4A2) intracellular pigments; basal layer with densely arranged clavate elements. Clamp connections present.

###### Habitat.

Solitary to scattered on soil, the humus layer, or grasslands in mixed coniferous and broad-leaved forests.

###### Distribution.

Known from Heilongjiang, Jilin, and Inner Mongolia, China.

###### Additional specimens examined.

CHINA • Jilin: Jilin City, Tumenling, 17 July 2003, coll. T. Bau, HMJAU5286; Jilin City, Beishan Park, 43°50'25"N, 126°31'15"E, alt. ca. 260 m, 2 August 2025, coll. J.H. Zu, FJAU79214; Changchun City, Jilin Agricultural University, 43°48'28"N, 125°25'16"E, alt. ca. 230 m, 8 July 2008, coll. T. Bau, HMJAU6441; 19 July 2022, coll. S.E. Wang, FJAU79197; Changchun City, Jingyuetan National Forest Park, 43°46'1"N, 125°29'19"E, alt. ca. 235 m, Changchun City, Jingyuetan National Forest Park, 43°46'1"N, 125°29'19"E, alt. ca. 240 m, 14 September 2016, coll. T. Bau, HMJAU37536; 19 August 2023, coll. M. Liu, FJAU79188; 30 July 2024, coll. Y.F. Han, J.L. Wei and Z.Q. Chen, FJAU79189, FJAU79199, FJAU79207; 11 August 2023, coll. J.L. Wei and M. Liu, FJAU79210, FJAU79211, FJAU79212; Changchun City, Nanhu Park, 43°51'13.47"N, 125°18'7.15"E, alt. ca. 220 m, 17 August 2022, coll. W.N. Hou, FJAU79192; Jiaohe City, Qianjin Forest Farm, 43°57'10"N, 127°42'7"E, alt. ca. 650 m, 23 July 2022, coll. W.N. Hou, FJAU79209; Jiaohe City, Shansongling, 43°40'51"N, 127°12'0"E, alt. ca. 430 m, 26 July 2022, F. Guo, FJAU79213; 29 July 2023, coll. S.E. Wang, FJAU79205; Jiaohe City, Red Leaf Valley, 43°44'23"N, 127°5'5"E, alt. ca. 430 m, 31 July 2023, FJAU79191, FJAU79193; Jiaohe City, Laoyeling, 43°40'57"N, 127°11'54"E, 430 m, 28 July 2023, coll. X. Wang, FJAU79195; Jiaohe City, Xinzhan Town, 25 August 2023, coll. X.Y. Zhou, FJAU79194; Jiaohe City, Lafashan National Forest Part, 43°48'21"N, 127°23'50"E, alt. ca. 370 m, 5 September 2024, coll. J.H. Zu, FJAU79208; Dunhua City, Beishan Park, 43°22'50"N, 128°13'20"E, alt. ca. 570 m, 17 August 2024, coll. X.Y. Zhou and R.H. Lin, FJAU79198, FJAU79215; Ji’an City, Yushan Park, 41°8'5"N, 126°11'2"E, alt. ca. 270 m, 24 July 2025, coll. X.Y. Zhou, FJAU79202, FJAU79203; 26 July 2025, coll. X.Y. Zhou, FJAU79204; Huadian City, Hongshi National Forest Park, 42°50'01"N, 127°07'57"E, alt. ca. 490 m, 24 June 2021, 15 August 2025, coll. T.Y. Zhang, FJAU79216; 16 August 2025, coll. Y. Wang, FJAU79217; Inner Mongolia: Tongliao City, Horqin Left Wing Rear Banner, 5 August 2021, coll. L.Y. Zhu, FJAU79196; Tongliao City, Horqin Left Middle Banner, Wusitu Nature Reserve, 21 August 2022, coll. W.N. Hou, FJAU79206; Heilongjiang: Helen City, Chenjiadian Forest Farm, 47°40'6"N, 127°18'26"E, alt. ca. 280 m, 12 August 2025, coll. X.Y. Zhou, FJAU79200, FJAU79201.

###### Notes.

Morphologically and phylogenetically, *Lepiota subforquignonii* is closely related to *Lepiota forquignonii* Quél., as both species share small basidiomata, a pileus with brownish squamules, and similarly sized basidiospores.

However, *L. forquignonii* can be easily distinguished microscopically by its cheilocystidia, which lack septa and branches, and relatively narrower basidiospores with a higher average Q value (Qav = 1.8–1.9). Macroscopically, the pileus of *L. forquignonii* exhibits dark gray-brown squamules often with a distinct greenish or olive tinge, contrasting with the pastel brown to garnet red squamules of the new species (Vellinga 2001c).

##### Lepiota

Taxon classificationAnimaliaAgaricalesAgaricaceae

sect. Stenosporae J.E. Lange ex Reschke & Sarawi, in Sarawi, Piepenbring & Reschke, Fungal Syst. Evol. 15: 238

223E11F3-8C41-5AF1-81AB-0263AE161086

###### Type species.

*Lepiota castanea* Quél.

##### Lepiota brunneotacta

Taxon classificationAnimaliaAgaricalesAgaricaceae

T. Bau & X.Y. Zhou sp. nov.

A3F37FD4-AD9D-58C9-8FAE-D1B7988747A6

Index Fungorum: IF905887

Figs 21a, 35

###### Etymology.

From Latin brunneus (brown) and tactus (touched), referring to the stipe bruising reaction that darkens to light brown upon handling.

###### Holotype.

CHINA • Fujian: Fuzhou City, Changle District, 24 August 2024, H.B. Song, FJAU78296.

###### Diagnosis.

*Lepiota brunneotacta* is primarily characterized by its pileus densely covered with grayish orange squamules; a distinct bruising reaction on the stipe, which darkens to light brown upon handling; cylindrical, projectile-shaped basidiospores (8.3–9.6 × 3.2–4.1 μm) bearing a prominent saccate spur; and a trichoderm pileipellis.

###### Description.

Basidiomata small. Pileus 0.8–1.4 cm in diam., plano-convex to applanate; surface cream, densely covered with grayish orange (6B3–B7) fine, easily disrupted squamules; squamules at the disc brownish orange (6C7–C8), densely packed and remaining intact. Lamellae free, white to cream, crowded; lamellulae in 2–3 tiers; edge concolorous. Stipe 1.4–2.9 cm long, 0.1–0.2 cm thick, subcylindrical, slightly broadening towards the base, cream to grayish orange; middle and lower parts bruising darker to light brown (7D6–D8) upon handling; distinct annular zone absent. Context in pileus white, thin; in stipe white to pale cream, near the base turning light brown. Odor and taste not recorded.

Basidiospores [40/2/2] 8.3–9.6 × 3.2–4.1 μm, avl × avw = 8.9 × 3.6 μm, Q = 2.10–2.95, Qav = 2.45, spurred, commonly with a distinct saccate spur at the base, non-dextrinoid to weakly dextrinoid, hyaline, thin-walled, non-metachromatic. Basidia 16–24 × 7–9 μm, clavate, thin-walled, 4-spored (rarely 2-spored). Cheilocystidia 23–42 × 5–11 μm, clavate to oblong, thin-walled. Pleurocystidia absent. Pileus covering a trichoderm: terminal cells 62–192 × 7–13 µm, erect, cylindrical to clavate, with rounded apices and slightly thick walls, with grayish orange (6B3–B5) intracellular pigments. Stipe covering similar to pileus covering in cellular shape and pigmentation, but structured as a shorter trichoderm; terminal cells 36–148 × 9–13 µm, appressed, cylindrical to clavate. Clamp connections present.

###### Habit and habitat.

Scattered on grasslands within broadleaf forests.

###### Distribution.

Currently known from Fujian and Zhejiang Province, China.

###### Additional specimens examined.

CHINA • Zhejiang: Hangzhou City, Zhejiang University, 5 July 2021, L.Y. Zhu, FJAU78187.

###### Notes.

Macroscopically, *L. fulvescens* closely resembles species within the orange-brown complex of *Lepiota* sect. *Stenosporae*, as they share similar orange-brown pileal squamules (Zhou and Bau 2026). However, *L. fulvescens* can be readily distinguished from most members of this complex by its distinct bruising reaction, where the stipe darkens upon handling. Although *L. sinocastanea* T. Bau & X.Y. Zhou, another member of this complex, also exhibits a bruising stipe, it can be separated from *L. fulvescens* by its lamellae, which also discolor upon injury—a reaction absent in *L. fulvescens* (Zhou and Bau 2026).

Phylogenetically, *L. fulvescens* is closely related to *L. aurantiicolor* Sarawi, *L. brunneopileata* A. Rehman, Afshan, Usman & Khalid, *L. alopochroa* (Berk. & Broome) Sacc., and *L. annimonstra* Kun L. Yang & Jia Y. Lin. Morphologically, however, *L. aurantiicolor* (6.0–7.4 × 3.1–3.9 μm), *L. brunneopileata* (4.7–7.0 × 2.6–4.1 μm), and *L. annimonstra* (6.0–8.0 × 3.5 μm) produce significantly smaller basidiospores compared to *L. fulvescens* (8.3–9.6 × 3.2–4.1 μm) (Rehman et al. 2024; Sarawi et al. 2025a; Yang et al. 2026). Furthermore, *L. alopochroa* differs in possessing narrowly clavate to cylindrical cheilocystidia, contrasting with the clavate to oblong ones observed in *L. fulvescens* (Sysouphanthong et al. 2011).

##### Lepiota changbaiensis

Taxon classificationAnimaliaAgaricalesAgaricaceae

T. Bau & X.Y. Zhou sp. nov.

5AF946B1-8DB5-5DA3-B93E-C591E9308688

Index Fungorum: IF905888

Figs 21b, c, 36

###### Etymology.

“*changbaiensis*” refers to the type locality, Changbai Mountain, located in Jilin Province, Northeastern China, which is a major biodiversity hotspot for mycological research.

###### Holotype.

CHINA • Jilin: Changbai Mountain National Forest Park, 42°20'13"N, 128°7'20"E, alt. ca. 857 m, 13 August 2025, Y.F. Han, FJAU78297.

###### Diagnosis.

*Lepiota changbaiensis* is characterized by its orange-brown pileal squamules, capitate cheilocystidia, and basidiospores measuring 9.7–11.6 × 3.0–4.4 μm. it differs from *L. dolichospora* by having markedly smaller basidiospores and from *L. subcastanea*—which shares similar spore dimensions—by possessing capitate cheilocystidia, contrasting with the narrowly clavate or utriform cheilocystidia characteristic of the latter.

###### Description.

Basidiomata small. Pileus 0.8–1.6 cm in diam., plano-convex to applanate; surface cream, densely covered with brownish orange (7C4–C8) fine floccose squamules arranged in tufts; disc squamules darker and more densely packed; margin with white or concolorous veil remnants. Context white, thin. Lamellae free, white to cream, crowded; lamellulae present; edge concolorous. Stipe 2.3–3.6 cm long, 0.2–0.3 cm thick, subcylindrical, slightly broadening towards the base, cream; annular zone present; nearly glabrous above, densely covered with brownish orange floccose squamules arranged in discontinuous concentric bands below. Odor and taste not recorded.

Basidiospores [40/2/2] 9.7–11.6 × 3.0–4.4 μm, avl × avw = 10.5 × 3.9 μm, Q = 2.36–3.66, Qav = 2.67, spurred, commonly with a distinct saccate spur at the base, non- to weakly dextrinoid, hyaline, thin-walled, non-metachromatic. Basidia 17–28 × 7–10 μm, clavate, thin-walled, 4-spored, occasionally 2-spored. Cheilocystidia 21–37 × 5–10 μm, clavate to fusiform with a capitate apex, thin-walled. Pleurocystidia absent. Pileus covering a trichoderm: terminal elements 81–200 × 11–17 μm, subcylindrical, slightly thick-walled, grayish orange (6B3–B5), with brownish orange intracellular pigments. Stipe covering similar to pileus covering in layer structure, cellular shape, and pigmentation, but structured as a shorter trichoderm; terminal cells 36–140 × 8–21 µm, appressed, cylindrical to clavate. Clamp connections present.

###### Habit and habitat.

Solitary on humus-rich soil in broadleaf forests.

###### Distribution.

Currently known only from Jilin Province, China.

###### Additional specimens examined.

CHINA • Jilin: Changchun City, Jingyuetan National Forest Park, 43°46'1"N, 125°29'19"E, alt. ca. 240 m, 31 August 2022, F. Guo, FJAU78194.

###### Notes.

Macroscopically, *L. changbaiensis* possesses orange-brown tints on the pileal squamules, making it highly reminiscent of several species within the orange-brown complex of *Lepiota* sect. *Stenosporae* (Zhou and Bau 2026). However, its relatively large basidiospores distinguish it from a group of species with significantly smaller spores, such as *L. boudieri* Bres., *L. flavonigrescens* Sarawi & Reschke, *L. jilinensis* T. Bau & X.Y. Zhou, *L. microstenospora* T. Bau & X.Y. Zhou, *L. mandarina* Jun F. Liang & Zhu L. Yang, *L. sirupa* T. Bau & X.Y. Zhou, and *L. brunneoaurantia* Azeem & Jabeen 1 Stenospora (Liang 2016; Azeem et al. 2024; Sarawi et al. 2025a; Zhou and Bau 2026). Among the large-spored taxa in this section, *L. dolichospora* T. Bau & X.Y. Zhou produces basidiospores (11.5–14.1 × 3.3–4.7 μm) that are markedly larger (Zhou and Bau 2026) than those of *L. changbaiensis* (9.7–11.6 × 3.0–4.4 μm). While *L. subcastanea* Jun F. Liang & Zhu L. Yang shares similar spore dimensions with the new species, it can be clearly separated by the shape of its cheilocystidia, which are narrowly clavate or narrowly utriform (Liang 2016), contrasting with the capitate cheilocystidia observed in *L. changbaiensis*.

##### Lepiota flavoviridis

Taxon classificationAnimaliaAgaricalesAgaricaceae

T. Bau & X.Y. Zhou sp. nov.

34F1CD88-8DBD-5786-8866-4816F5D4B027

Index Fungorum: IF905889

Figs 21d–i, 37

###### Etymology.

“*flavoviridis*” (from Latin flavus = yellow, and viridis = green) refers to the striking grayish yellow to grayish green coloration of the pileal squamules, which is a distinctive hallmark of this new species.

###### Holotype.

CHINA • Jilin: Huadian City, Hongshi National Forest Park, 42°50'01"N, 127°07'57"E, alt. ca. 490 m, 27 August 2023, T. Bau and X.Y. Zhou, FJAU78219.

###### Diagnosis.

*Lepiota flavoviridis* is recognized by its overall cream-yellow to verona green basidiomata; its stipe surface is densely ornamented with concolorous squamules arranged in interrupted annular bands or a sub-serpentine pattern; basidiospores are spurred and rectangular in profile.

###### Description.

Basidiomata small. Pileus 0.5–3.5 cm in diam., initially hemispherical to plano-convex, expanding to applanate; surface densely covered with grayish yellow (1B4–B8) to grayish green (1C4–C8) fine squamules; squamules at the disc denser and darker, occasionally dark yellow (4C7–C8); margin occasionally with velar remnants, concolorous with the squamules. Lamellae free, grayish yellow to grayish green, becoming brown (6E5–E8) when dried, crowded; lamellulae present; edge concolorous. Stipe 1.4–7.5 cm long, 0.2–0.4 cm thick, subcylindrical, concolorous with the pileus; annular zone present; nearly glabrous above, covered with concolorous squamules arranged in serpentine, discontinuous concentric bands below. Context grayish yellow, thin; in stipe white to grayish yellow, near the base turning brown (7E5–7E8). Basidiomata easily stain the fingers grayish yellow upon handling. Odor and taste not recorded.

Basidiospores [90/3/3] 7.7–9.0(–9.5) × (2.5–)2.8–3.5(–3.8) μm, Q = (2.01–)2.33–3.26, rectangular in lateral view, base more or less with an elongated spur, slightly ventricose in adaxial view, occasionally slightly depressed at the abaxial base, oblong-ellipsoid in frontal view, hyaline, smooth, dextrinoid, non-metachromatic. Basidia 19–26 × 7–8 μm, clavate, thin-walled, 4-spored (rarely 2-spored). Cheilocystidia 12–46 × 5–14 μm, clavate to subfusiform, occasionally with an apical finger-like projection or a transverse septum, thin-walled. Pleurocystidia absent. Pileus covering a trichoderm: terminal elements 41–151 × 7–14 μm, subcylindrical, slightly thick-walled, with yellowish white (3A2) intracellular pigments. Stipe covering similar to pileus covering in cellular shape and pigmentation, but structured as a shorter trichoderm; terminal cells 29–113 × 8–17 µm, appressed, cylindrical to clavate. Clamp connections present.

###### Habit and habitat.

Solitary to scattered on soil in broad-leaved forests.

###### Distribution.

Currently known only from Jilin Province, China.

###### Additional specimens examined.

CHINA • Jilin: Jiaohe City, Qianjin Forest Farm, 43°57'10"N, 127°42'7"E, alt. ca. 650 m, 24 June 2021, 25 August 2023, S.Y. Li and M. Liu, FJAU78233, FJAU78234; 6 September 2024, Y.F. Han and T.Y. Zhang, JAU78290, FJAU78291; Huadian City, Hongshi National Forest Park, 42°50'01"N, 127°07'57"E, alt. ca. 490 m, 27 August 2023, T. Bau and X.Y. Zhou, FJAU78218; 27 August 2023, H. Cheng, FJAU78217; 16 August 2024; X.Y. Zhou, Z.Q. Chen and Y.F. Han, FJAU78236, FJAU78237, FJAU78238, FJAU78241, FJAU78242, FJAU78243; 23 August 2024, X.Y. Zhou, FJAU78239, FJAU78240; 4 September 2024, Y.F. Han, FJAU78289; 22 September Q.R. Liu, FJAU78235; 30 July 2025, Y.F. Han, FJAU78245; 31 July 2025, T.Y. Zhang, FJAU78292, FJAU78293; 15 August 2025, Y.F. Han and W. Sun, FJAU78244, FJAU78294.

###### Notes.

Macroscopically, *Lepiota flavoviridis* is reminiscent of *L. citrophylla* (Berk. & Broome) Sacc. and *L. subcitrophylla* Hongo due to their similar color. Nevertheless, *L. citrophylla* is characterized by significantly shorter and broader basidiospores (5.5–8.5 × 2.6–4.5 μm, Q = 1.4–2.2) (Sysouphanthong et al. 2020b), which contrast with the more elongated and narrower spores of the present species. *Lepiota subcitrophylla* is readily distinguished by its specific cyanescent reaction, in which the basidiomata turn blue upon injury. Furthermore, although *L. xanthophylla* also exhibits prominent yellowish basidiomata, it is easily differentiated by its ellipsoid to long-ellipsoid basidiospores that lack the spurred base typical of the current taxon (Yang et al. 2019).

##### Lepiota fulvoolivacea

Taxon classificationAnimaliaAgaricalesAgaricaceae

T. Bau & X.Y. Zhou sp. nov.

512A6C34-C3D3-5383-AFD4-86245B70505D

Index Fungorum: IF905890

Figs 21j–l, 38

###### Etymology.

“*fulvoolivacea*” (from Latin fulvus = yellow-brown, and olivaceus = olive-green) refers to the characteristic color combination of the basidiomata, highlighting the brownish-orange and olivaceous tones present on the pileal squamules.

###### Holotype.

CHINA • Inner Mongolia: Tongliao City, Daqinggou National Nature Reserve, 42°46'38"N, 122°11'46"E, alt. ca. 240 m, 31 July 2025, T. Bau and X.Y. Zhou FJAU78250.

###### Diagnosis.

*Lepiota fulvoolivacea* is phylogenetically closely related to *L. rhodophylla*, but can be readily distinguished by its dark olive-brown pileal squamules at the disc, and noticeably smaller basidiospores (avl × avw = 7.5 × 3.3 μm) compared to the larger spores (avl × avw = 8.2–9.2 × 3.7–4.0 μm) characteristic of *L. rhodophylla*.

###### Description.

Basidiomata small. Pileus 1.8–3.3 cm in diam., plano-convex; surface cream, densely covered with brownish orange (6C3–C5) to olive (3D4–D6) small squamules; squamules at the disc darker, olive brown (3E5–E8); margin with veil remnants. Context cream, thin. Lamellae free, white to cream, crowded; lamellulae present; edge concolorous. Stipe 4.8–5.3 cm long, 0.2–0.3 cm thick, subcylindrical, cream to grayish red (7C3–C6); covered with white to olive longitudinal fibrillose squamules; distinct annular zone absent. Odor and taste not recorded.

Basidiospores 6.9–8.4 × 2.8–3.8 μm, avl × avw = 7.5 × 3.3, Q = 1.94–2.56, Qav = 2.31, spurred, appearing rectangular in profile, sub-triangular in side view, hyaline, smooth, slightly thick-walled, non- to weakly dextrinoid, non-metachromatic. Basidia 15–25 × 6–10 μm, clavate, thin-walled, 4-spored (rarely 2-spored). Cheilocystidia 19–39 × 6–12 μm, clavate to broadly clavate, sometimes constricted in the middle or upper portion into a capitate head, thin-walled. Pleurocystidia absent. Pileus covering composed of a cutis of repent hyphae: terminal cells 5–20 μm diam., containing pale olivaceous to olive-brown intracellular pigments. Clamp connections present.

###### Habit and habitat.

Scattered on the humus layer in broad-leaved forests dominated by *Ulmus*.

###### Distribution.

Currently known from Inner Mongolia, China.

###### Additional specimens examined.

CHINA • Inner Mongolia: Tongliao City, Daqinggou National Nature Reserve, 42°46'38"N, 122°11'46"E, alt. ca. 240 m, 31 July 2025, W.N. Hou, FJAU78251.

###### Notes.

Macroscopically, *L. ochraceoviridis* is highly reminiscent of a series of greenish-brown taxa within *Lepiota* sect. *Stenosporae*, such as *L. griseovirens* Maire, *L. poliochloodes* Vellinga & Huijser, *L. grangei* (Eyre) Kühner, *L. hongshiensis* T. Bau & X.Y. Zhou, *L. brunneoolivacea* Sarawi, and *L. pilodes* Vellinga & Huijser. However, it can be easily distinguished from these look-alike species microscopically; the pileal squamules of the latter species are composed of a trichoderm, whereas *L. ochraceoviridis* possesses a cutis formed by prostrate hyphae (Zhou and Bau 2026).

Phylogenetically, sect. *Stenosporae* diverges into two major clades corresponding to their pileipellis structures: a trichoderm clade and a cutis clade. *L. ochraceoviridis* is nested within the cutis clade, which also includes known species such as *L. boudieri*, *L. andegavensis* Mornand, *L. aureofulvella* Sysouph., K.D. Hyde, Chukeat. & Vellinga, and *L. rhodophylla* Vellinga. Within this clade, *L. boudieri*, *L. andegavensis*, and *L. aureofulvella* all exhibit yellowish-brown to orange-brown pileal surfaces (Zhou and Bau 2026) which clearly differ from the greenish-brown tones of the new species. Furthermore, *L. andegavensis* produces significantly larger basidiospores (9.0–10.5 × 3.0–4.0 µm) compared to *L. ochraceoviridis* (6.9–8.4 × 2.8–3.8 µm) (Mornand 1982). *L. aureofulvella* possesses smaller, clavate to narrowly clavate cheilocystidia (17.5–27.5 × 5.0–10.0 µm), contrasting with the larger, clavate to broadly clavate ones (19–39 × 6–12 µm) observed in *L. ochraceoviridis* (Sysouphanthong et al. 2011). Finally, *L. rhodophylla* can be readily separated by its pinkish-brown pileal squamules, as opposed to the ochre-green coloration of the new species (Vellinga 2006).

##### Melanophyllum

Taxon classificationAnimaliaAgaricalesAgaricaceae

Velen., České Houby (Praze) 3: 569

E4BED639-AF61-597C-B535-77BE472DC74C

###### Type species.

*Melanophyllum haematospermum* (Bull.) Kreisel, Feddes Repert. Spec. Nov. Regni Veg. 95: 700 (1984).

##### Melanophyllum haematospermum

Taxon classificationAnimaliaAgaricalesAgaricaceae

(Bull.) Kreisel, Feddes Repert. Spec. Nov. Regni Veg. 95(9–10): 700 (1984)

A5A243E1-DEE8-5D39-8654-F1AF047C92B6

Fig. 22

###### Description.

Basidiomata small. Pileus 0.8–1.9 cm in diam., campanulate to hemispherical when young, becoming hemispherical to plano-convex with age; surface densely covered with orange (6A2) to grayish orange (6B4–B6), reddish brown (9E4–E9, 9F4–F9) pulverulent squamules, squamules darker at the center; margin with velar remnants, concolorous with the squamules. Lamellae free, violet-brown (11E4–E6) to grayish ruby (12E3–E5), crowded, with 3 tiers of lamellulae. Stipe 1.4–3.9 × 0.1–0.2 cm, subcylindrical, slightly broadening towards the base, grayish rose (12B2–B3) to grayish ruby (12C2–C3) to grayish red (#6F585B), densely covered with pulverulent squamules concolorous with the pileus; annular zone indistinct. Upon drying, the entire basidioma turns dark brown (8F3–F8).

**Figure 22.**
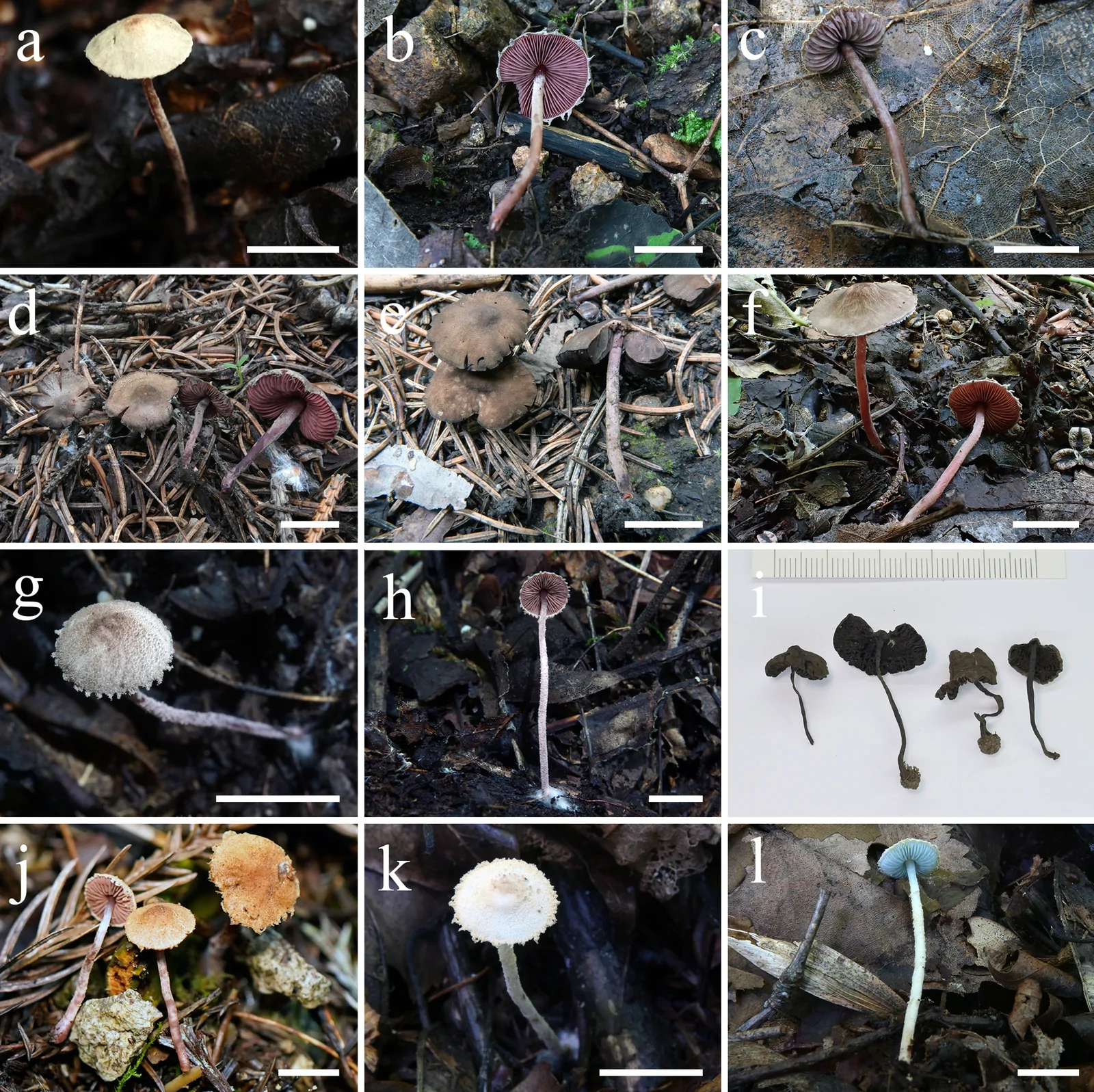
Basidiomata of *Melanophyllum* species. **a–i**. *Melanophyllum aff. haematospermum* (**a** FJAU79292; **b** FJAU79291; **c** FJAU79290; **d** FJAU79289; **e** FJAU79293; **f** FJAU79298; **g, h** FJAU79300; **i** left two FJAU79293; right two: FJAU79289); **j**. *Melanophyllum* sp. (HMJAU68255); **k, l**. *Melanophyllum eyrei* (FJAU79301). Scale bars: 1 cm.

Basidiospores 5.0–6.4 × 2.3–3.2 μm, avl × avw = 5.5 × 2.9 μm, Q = 1.67–2.25, Qav = 1.91, oblong to cylindrical, non-dextrinoid, thin-walled, minutely roughened under LM, distinct warts visible under SEM, grayish yellow (4C5–C6), metachromatic. Basidia 14–20 × 4–7 μm, clavate, pale yellow (543), thin-walled, with 4 (rarely 2) sterigmata. Cheilocystidia not observed. Pleurocystidia not observed. Pileus covering 2-layered: basal hyphae hyaline, thin-walled; apical layer composed of sparsely arranged hyphae and abundant inflated cells; inflated cells subglobose, obovoid, oblong to sphaeropedunculate, sometimes 2–5 connected in short moniliform chains, easily detachable, 19–42 μm, with light brown (6D5–D6) to brown (6E7–E8) intracellular pigments. Stipe covering similar to pileus covering in layer structure, cellular shape, and pigmentation. Clamp connections present.

**Figure 23.**
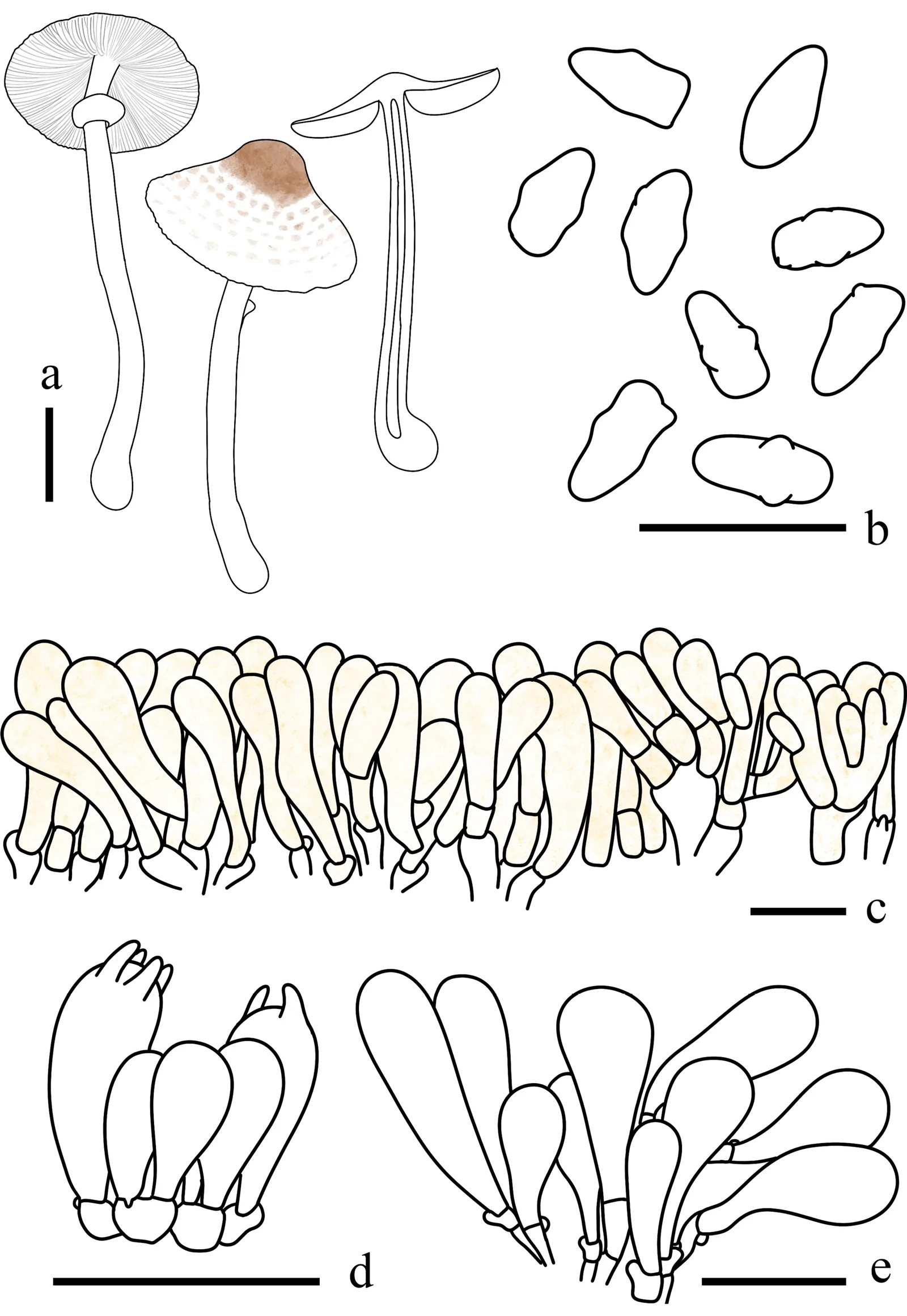
*Lepiota cristatosimilis* (FJAU78310). **a**. Basidiomata. **b**. Basidiospores. **c**. Pileus covering. **d**. Basidia and basidioles. **e**. Cheilocystidia. Scale bars: 1 cm (**a**); 10 μm (**b**); 50 μm (**c**); 20 μm (**d, e**).

###### Habit and habitat.

Solitary to scattered on humus soil in mixed coniferous and broad-leaved forests.

###### Distribution.

Widespread throughout the world.

###### Additional specimens examined.

CHINA • Jilin: Ji’an City, Wunufeng National Forest Park, 41°16'21"N, 126°7'39"E, alt. ca. 710 m, 25 July 2025, coll. X.Y. Zhou, FJAU79291; Heilongjiang: Helen City, Chenjiadian Forest Farm, 47°40'6"N, 127°18'26"E, alt. ca. 280 m, 12 August 2025, coll. X.Y. Zhou and W.N. Hou, FJAU79290, FJAU79292; Daxinganling area, Guqi Valley National Wetland Park, 52°19'53"N, 124°43'10"E, alt. ca. 350 m, 14 August 2025, coll. X.Y. Zhou, FJAU79293, FJAU79289.

**Figure 24.**
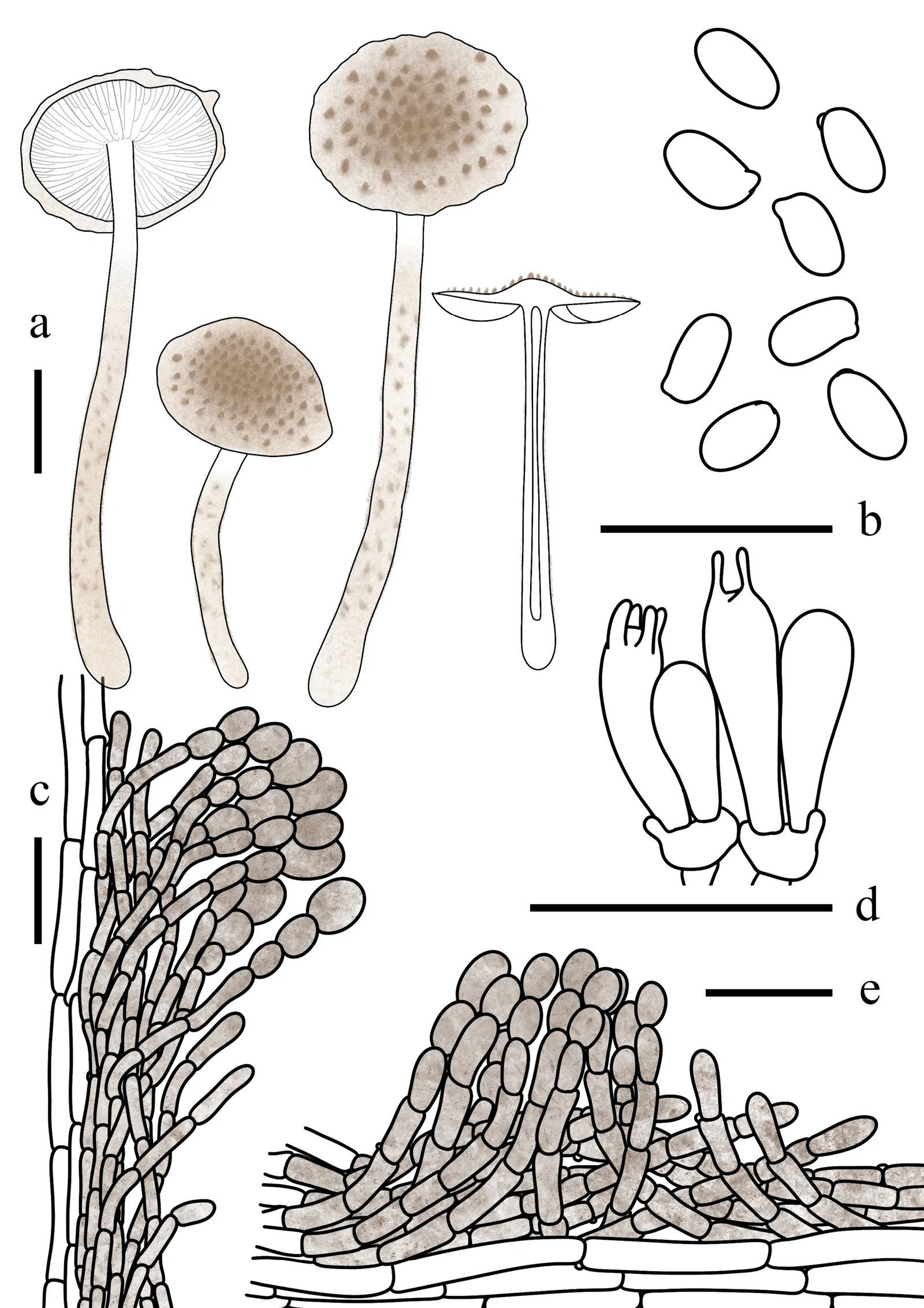
*Lepiota granulosquama* (FJAU79253). **a**. Basidiomata. **b**. Basidiospores. **c**. Stipe covering. **d**. Basidia and basidioles. **e**. Pileus covering. Scale bars: 1 cm (**a**); 10 μm (**b**); 50 μm (**c, e**); 20 μm (**d**).

###### Notes.

Traditionally, *M. haematospermum* is considered a cosmopolitan species; however, our multi-gene phylogenetic analysis indicates it is a cryptic species complex comprising several distinct lineages. This genetic differentiation is mirrored by morphological variations. For instance, our East Asian collections lack cheilocystidia and possess basidiospores measuring 5.0–6.4 × 2.3–3.2 μm, aligning closely with European core taxa but differing from the smaller spores (3.5–5.0 × 2.0–3.0 μm) reported in the Flora Fungorum Sinicorum (Candusso and Lanzoni 1990; Yang et al. 2019). Furthermore, this complex exhibits high phenotypic plasticity. We observed significant variations in the stipe-to-pileus ratio within a strongly supported clade (Velenovský 1921), suggesting microhabitat influences during basidioma development. Recent findings of atypical yellow-orange tones in phylogenetically nested specimens (Zhou and Bau 2024) further underscore this variability. From a generic perspective, *Melanophyllum* consistently clusters alongside *Cystolepiota* species, forming a monophyletic clade. To avoid premature nomenclatural combinations, we follow Zhou and Bau (2024) in temporarily adopting the broad concept of *Cystolepiota* s.l., whilst providing this detailed description to serve as a morphological baseline for future systematic revisions of the *M. haematospermum* complex.

**Figure 25.**
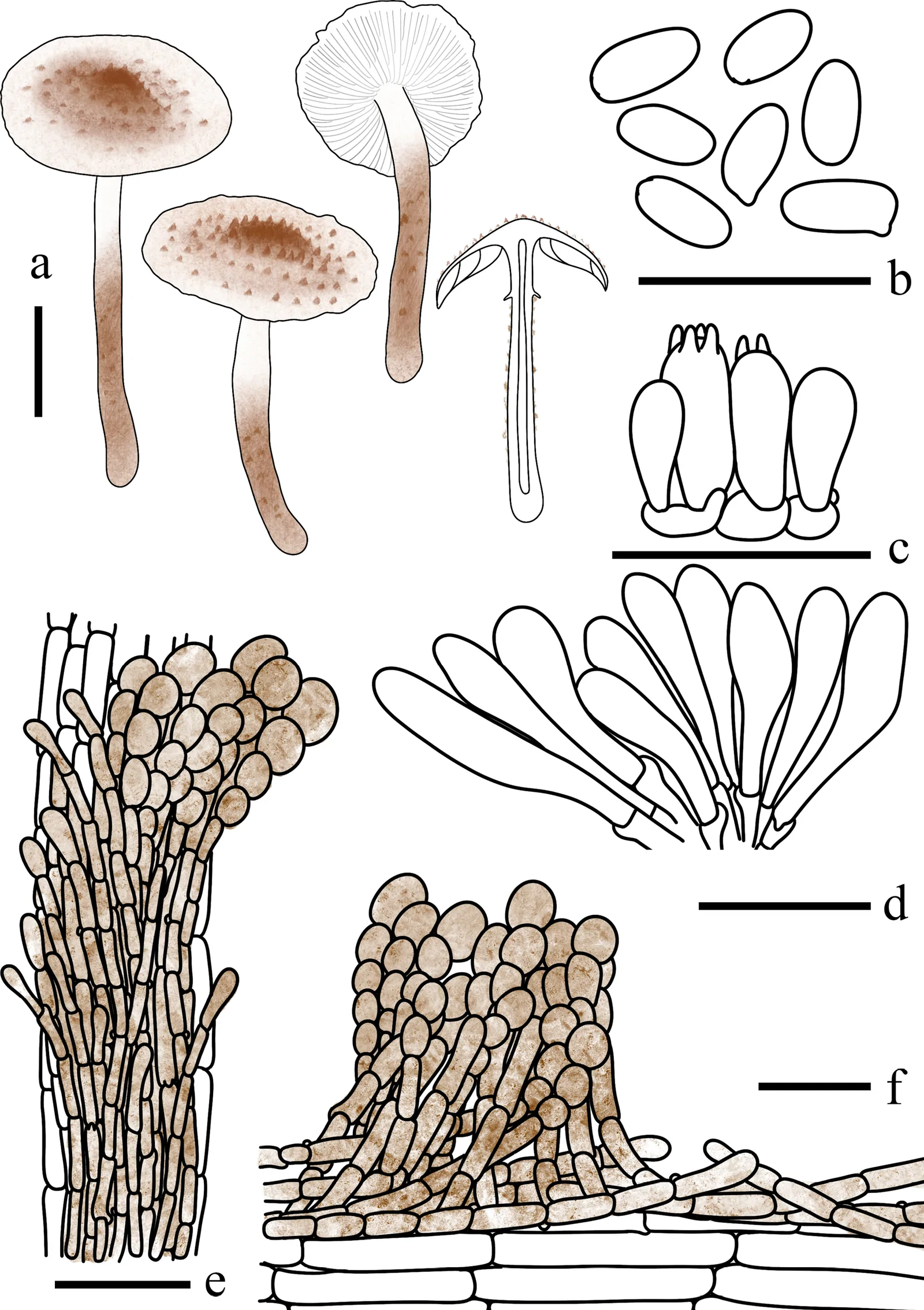
*Lepiota huadianensis* (FJAU79267). **a**. Basidiomata. **b**. Basidiospores. **c**. Basidia and basidioles. **d**. Cheilocystidia. **e**. Stipe covering. **f**. Pileus covering. Scale bars: 1 cm (**a**); 10 μm (**b**); 20 μm (**c, d**); 50 μm (**e, f**).

**Figure 26.**
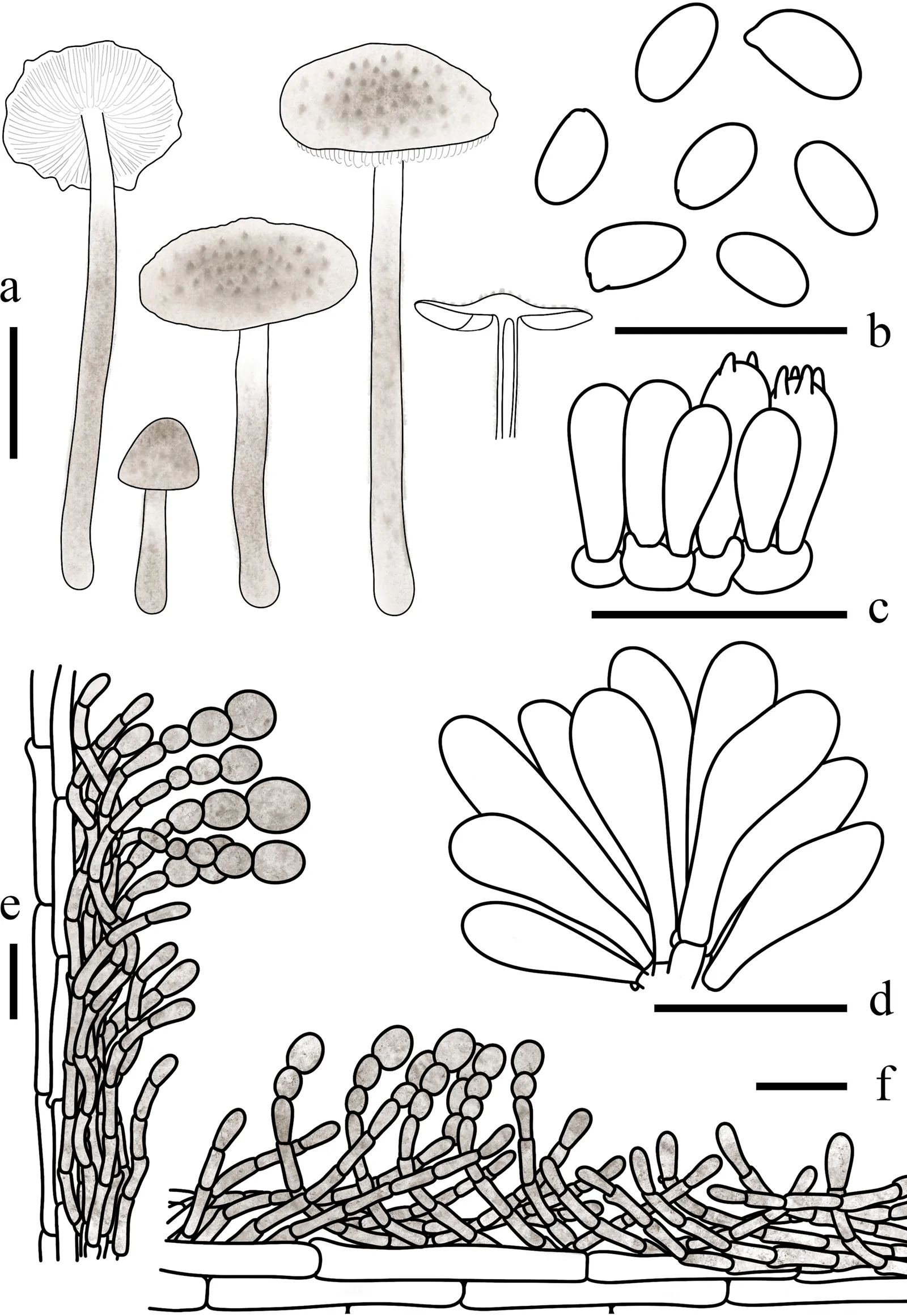
*Lepiota scabra* (FJAU79249). **a**. Basidiomata. **b**. Basidiospores. **c**. Basidia and basidioles. **d**. Cheilocystidia. **e**. Stipe covering. **f**. Pileus covering. Scale bars: 1 cm (**a**); 10 μm (**b**); 20 μm (**c, d**); 50 μm (**e, f**).

**Figure 27.**
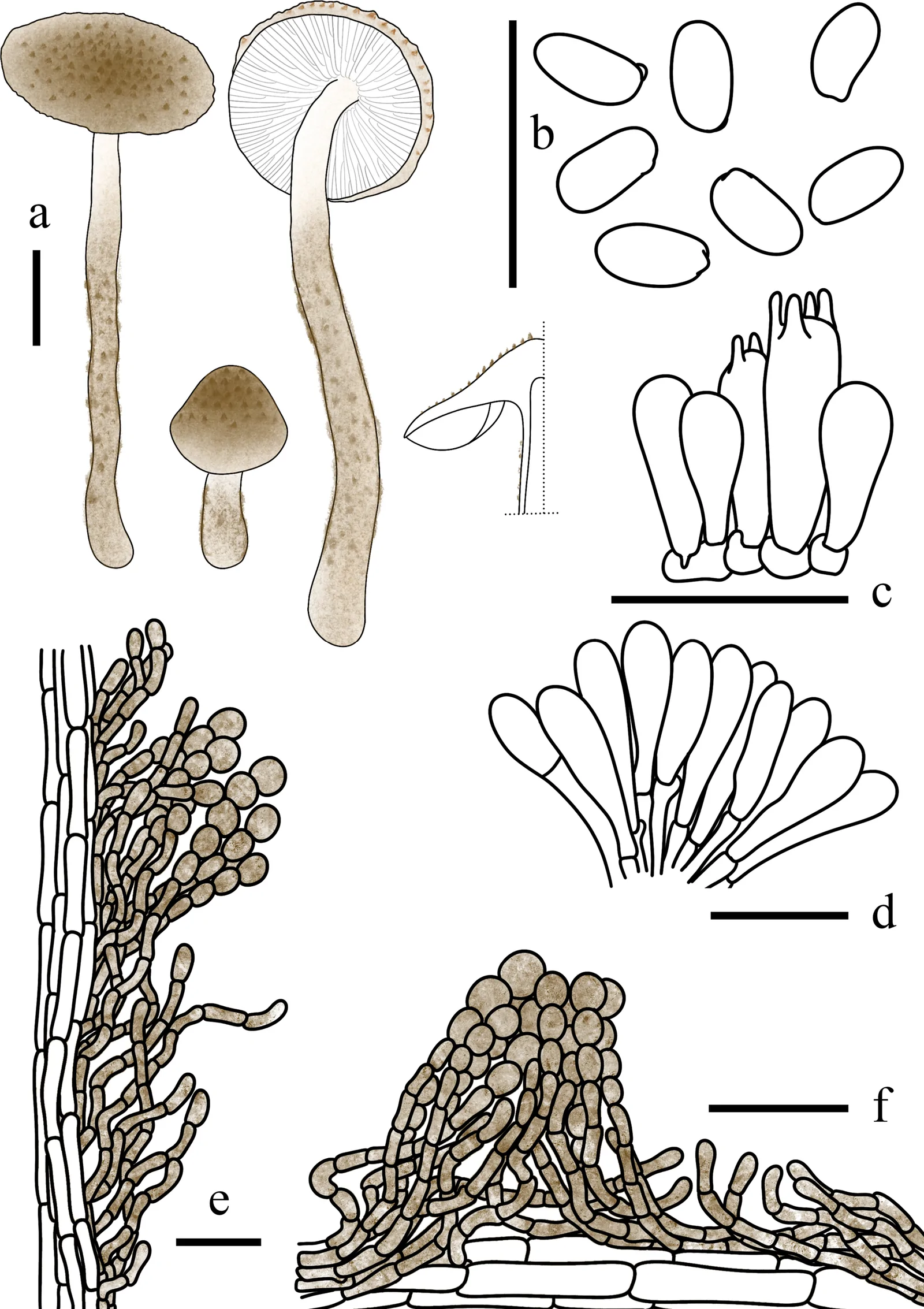
*Lepiota subhuadianensis* (FJAU79264). **a**. Basidiomata. **b**. Basidiospores. **c**. Basidia and basidioles. **d**. Cheilocystidia. **e**. Stipe covering. **f**. Pileus covering. Scale bars: 1 cm (**a**); 10 μm (**b**); 20 μm (**c, d**); 50 μm (**e, f**).

**Figure 28.**
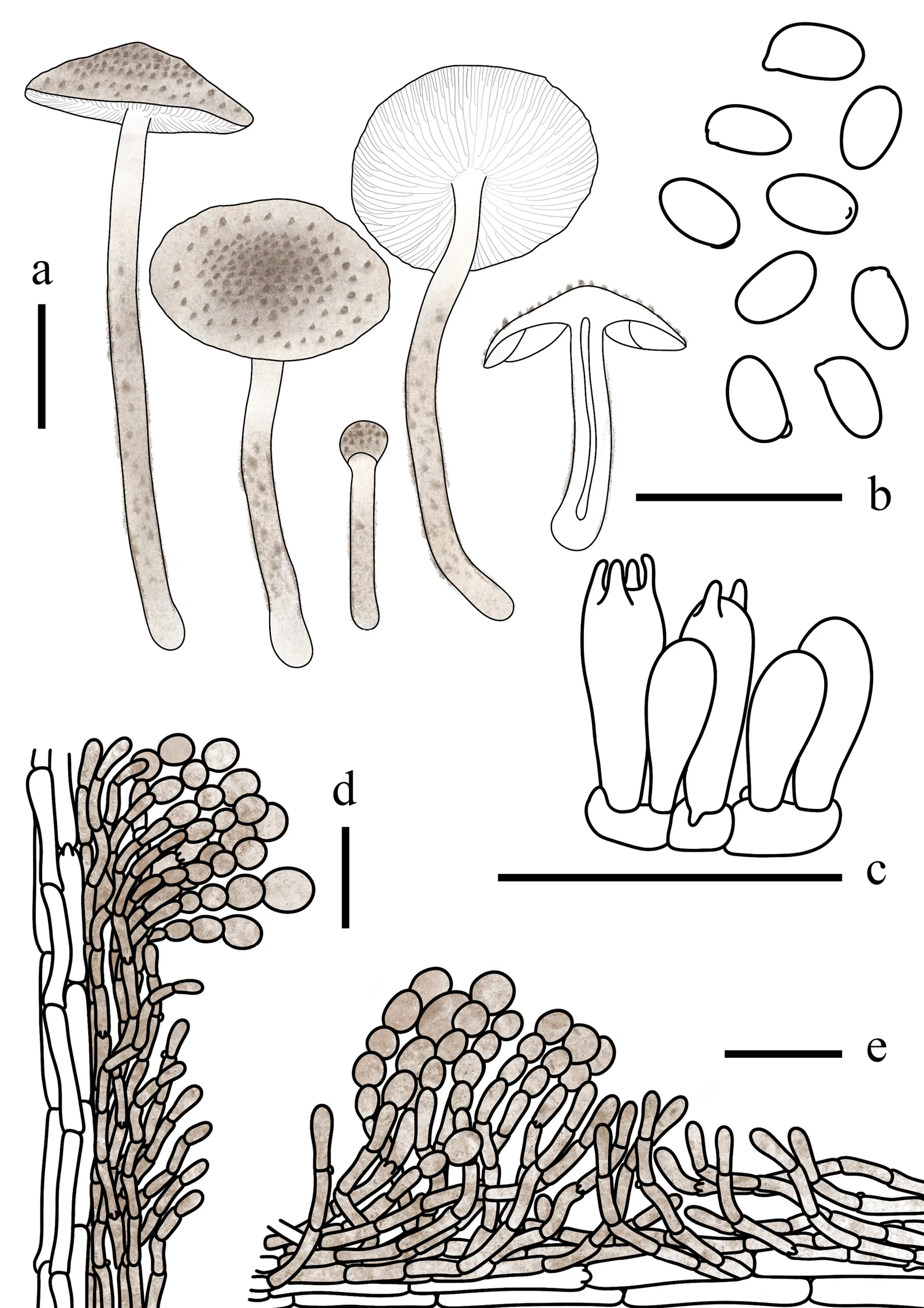
*Lepiota verruculosa* (FJAU79261). **a**. Basidiomata. **b**. Basidiospores. **c**. Basidia and basidioles. **d**. Stipe covering. **e**. Pileus covering. Scale bars: 1 cm (**a**); 10 μm (**b**); 20 μm (**c**); 50 μm (**d, e**).

**Figure 29.**
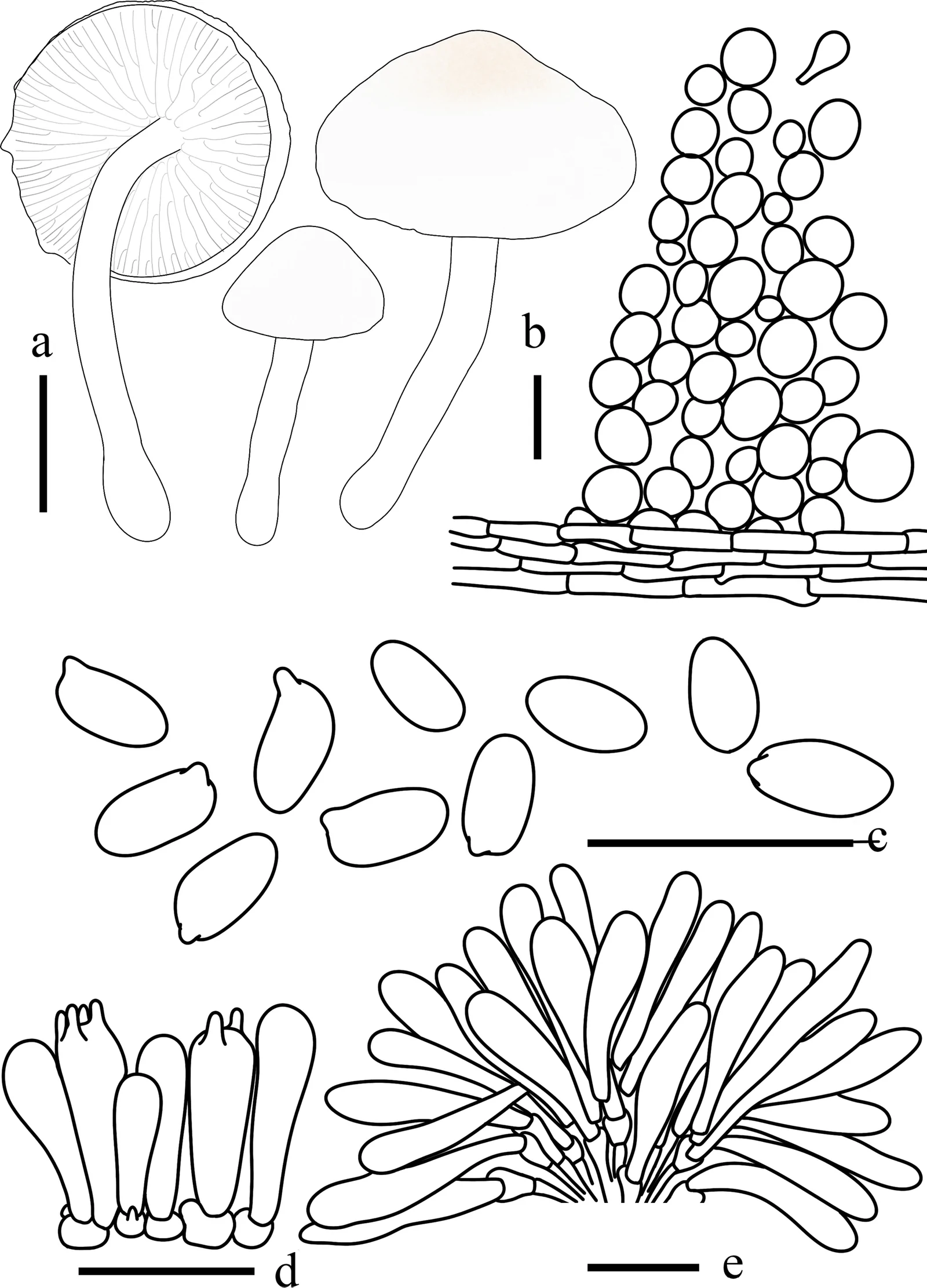
*Lepiota fugaciosquamosa* (FJAU79282). **a**. Basidiomata. **b**. Pileus covering. **c**. Basidiospores. **d**. Basidia and basidioles. **e**. Cheilocystidia. Scale bars: 1 cm (**a**); 50 μm (**b**); 10 μm (**c**); 20 μm (**d, e**).

**Figure 30.**
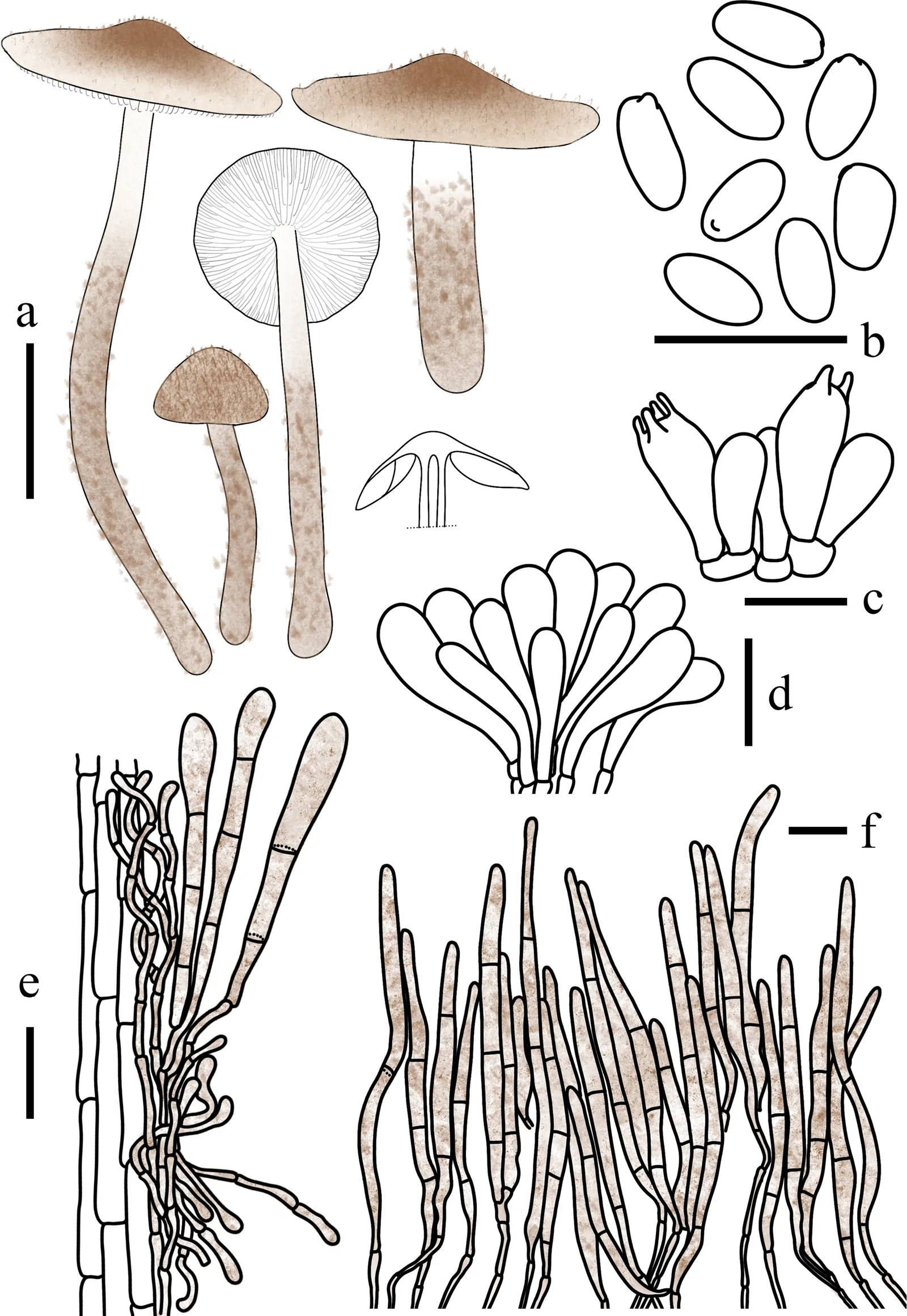
*Lepiota spinulosa* (FJAU78317). **a**. Basidiomata. **b**. Basidiospores. **c**. Basidia and basidioles. **d**. Cheilocystidia. **e**. Stipe covering. **f**. Pileus covering. Scale bars: 1 cm (**a**); 10 μm (**b**); 20 μm (**c, d**); 50 μm (**e, f**).

**Figure 31.**
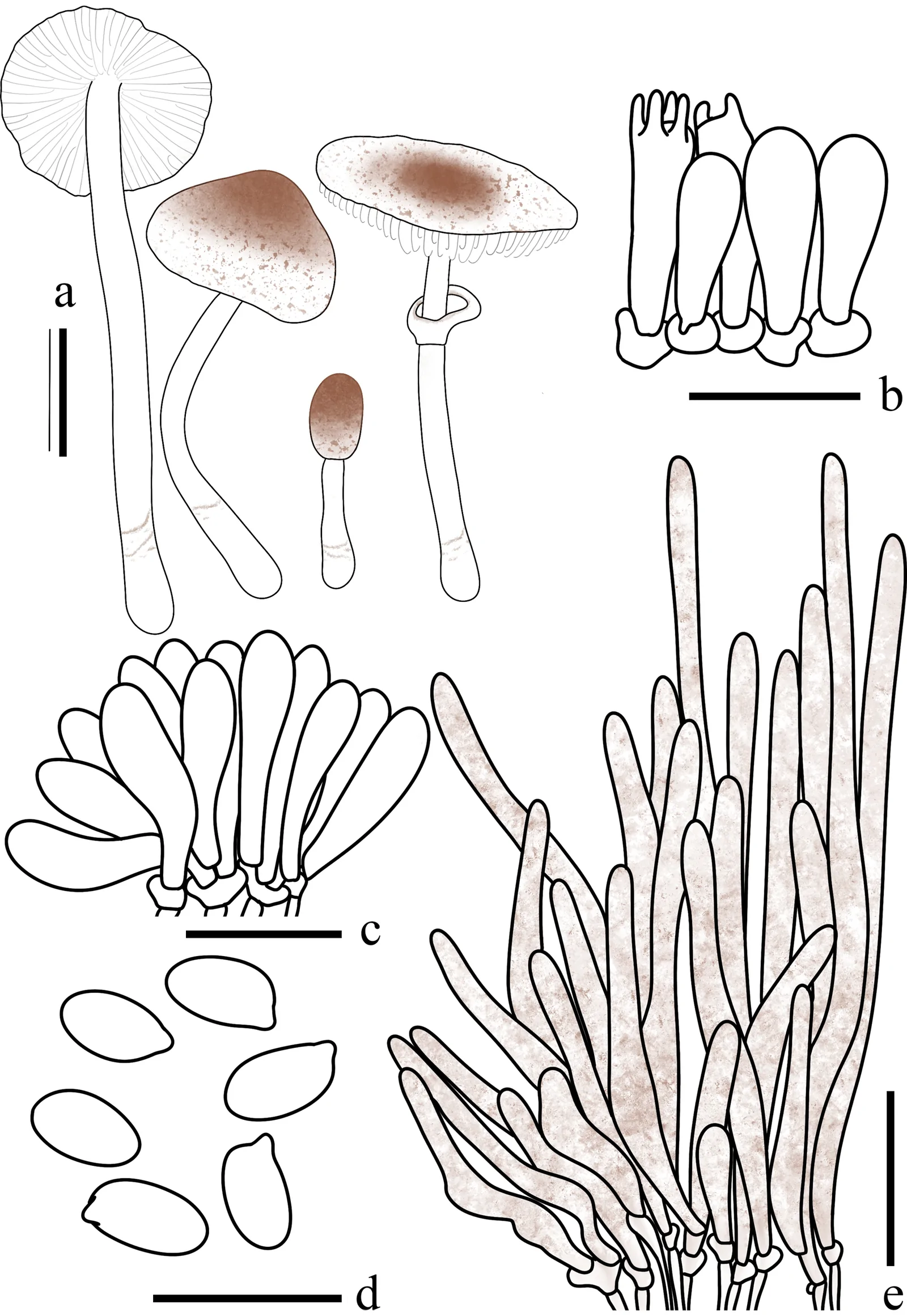
*Lepiota basozonata* (FJAU79181). **a**. Basidiomata. **b**. Basidia and basidioles. **c**. Cheilocystidia. **d**. Basidiospores. **e**. Pileus covering. Scale bars: 1 cm (**a**); 20 μm (**b, c**); 10 μm (**d**); 50 μm (**e**).

**Figure 32.**
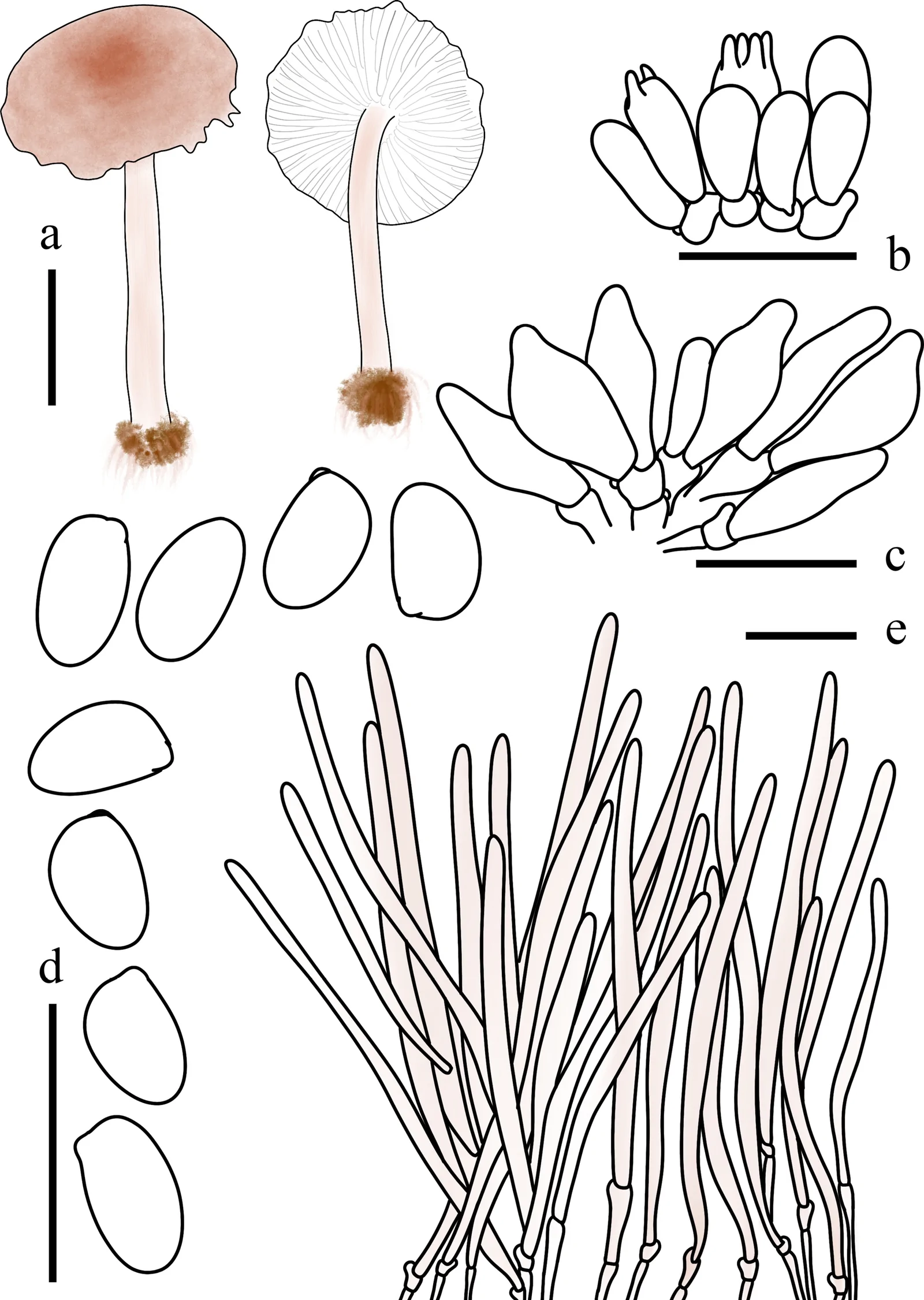
*Lepiota jianensis* (FJAU79175). **a**. Basidiomata. **b**. Basidia and basidioles. **c**. Cheilocystidia. **d**. Basidiospores. **e**. Pileus covering. Scale bars: 1 cm (**a**); 20 μm (**b, c**); 10 μm (**d**); 50 μm (**e**).

**Figure 33.**
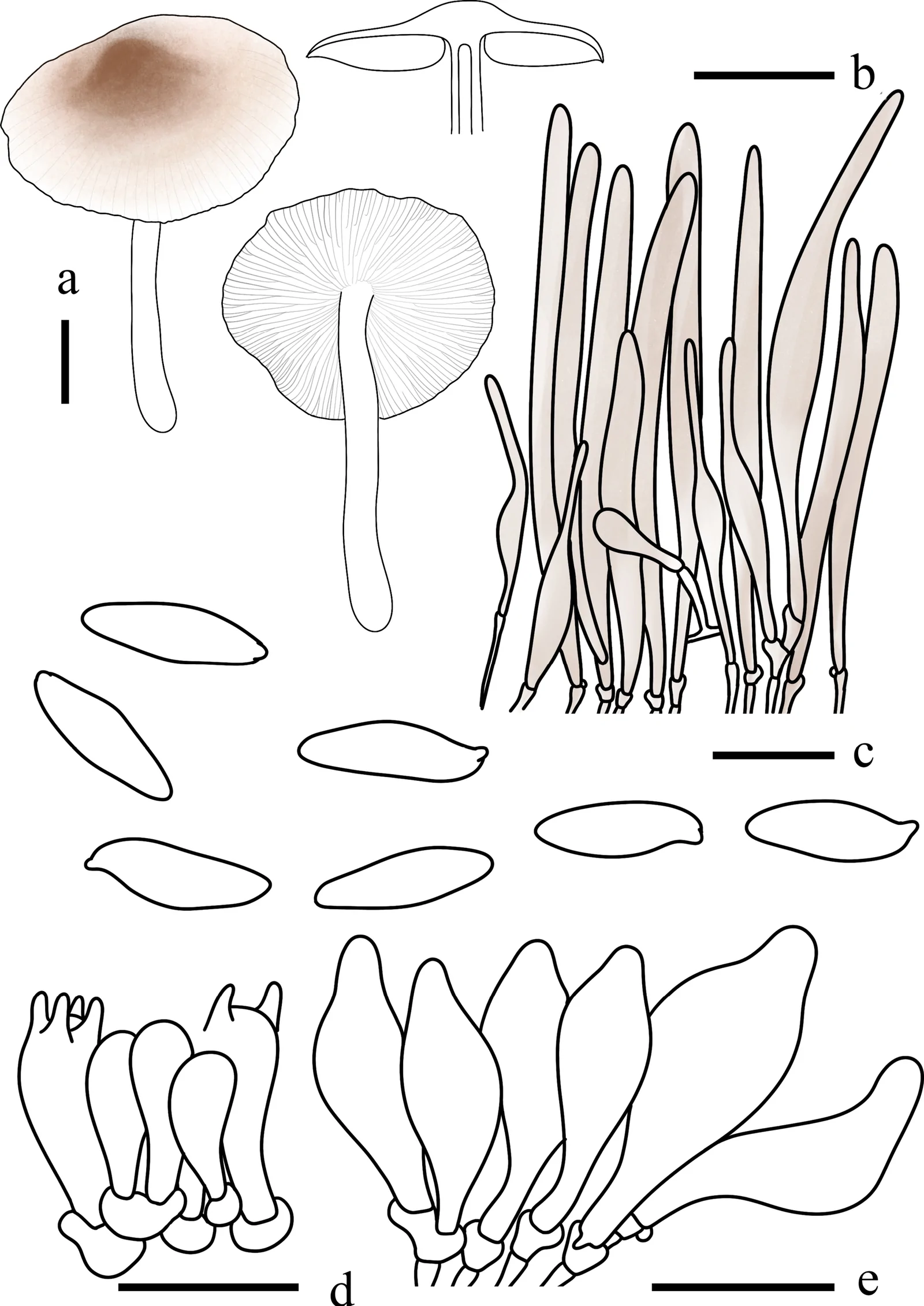
*Lepiota rostratospora* (FJAU79178). **a**. Basidiomata. **b**. Pileus covering. **c**. Basidiospores. **d**. Basidia and basidioles. **e**. Cheilocystidia. Scale bars: 1 cm (**a**); 50 μm (**b**); 10 μm (**c**); 50 μm (**d, e**).

**Figure 34.**
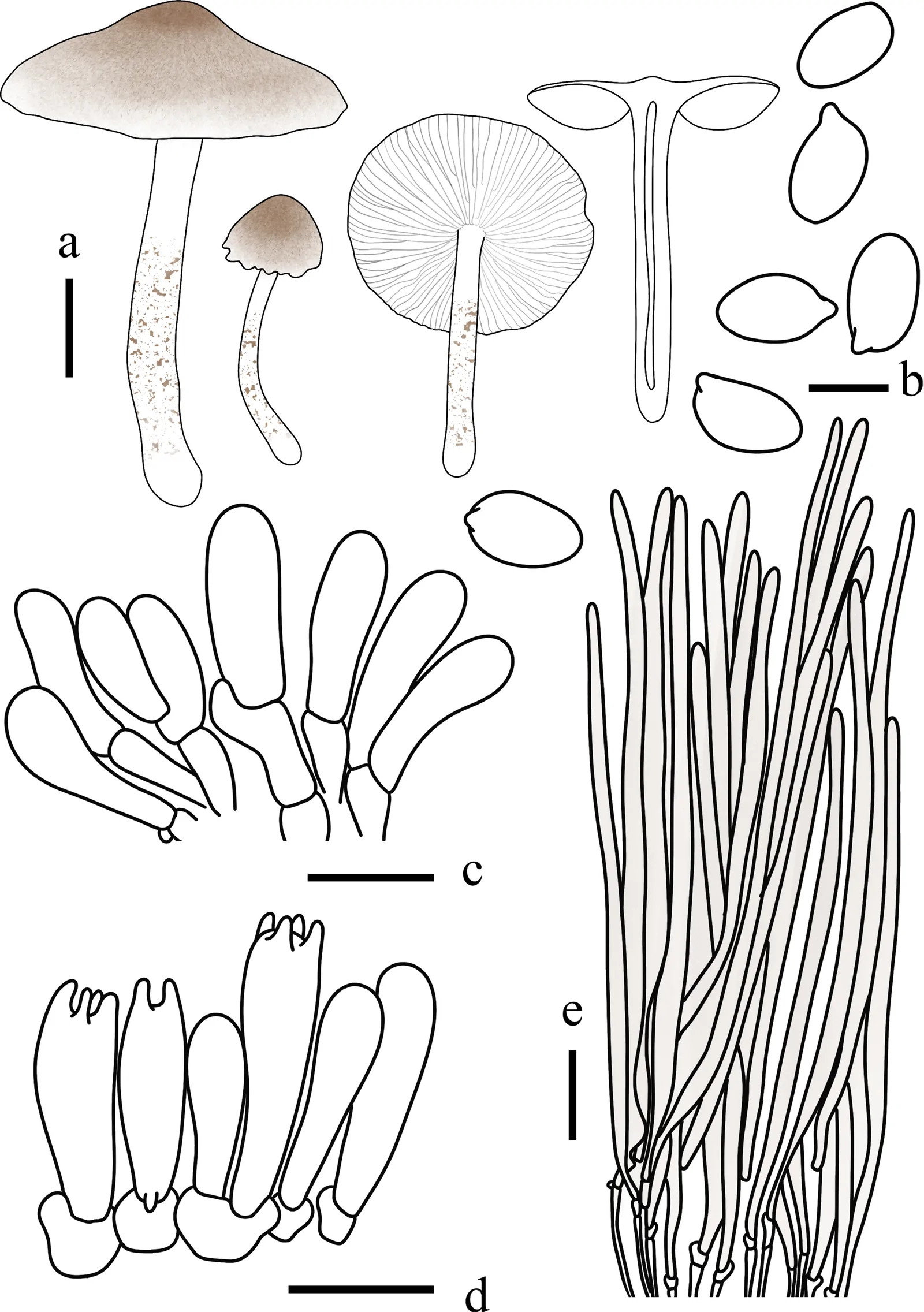
*Lepiota subforquignonii* (FJAU79190). **a**. Basidiomata. **b**. Basidiospores. **c**. Cheilocystidia. **d**. Basidia and basidioles. **e**. Pileus covering. Scale bars: 1 cm (**a**); 10 μm (**b**); 20 μm (**c, d**); 50 μm (**e**).

**Figure 35.**
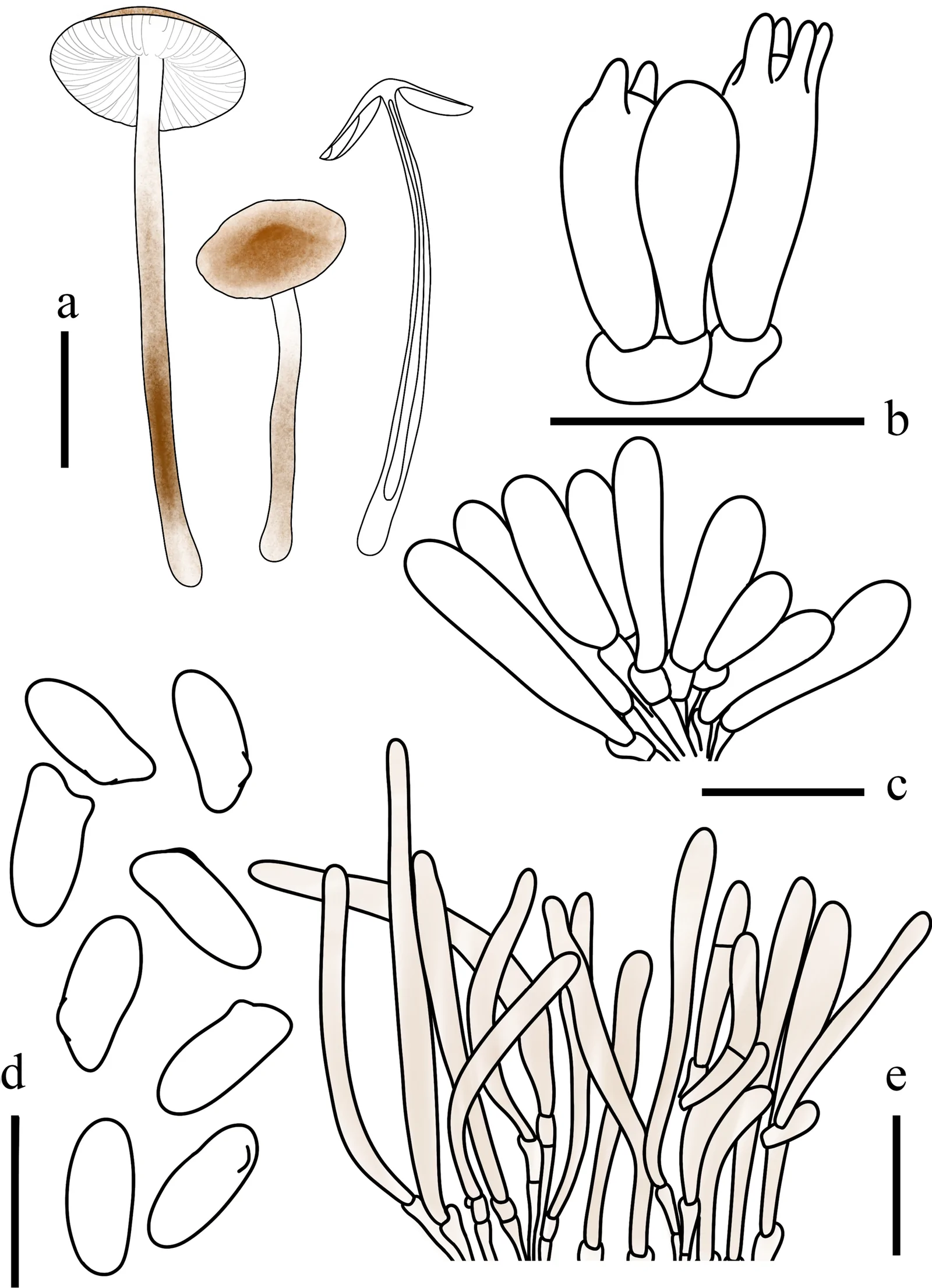
*Lepiota brunneotacta* (FJAU78296). **a**. Basidiomata. **b**. Basidia and basidioles. **c**. Cheilocystidia. **d**. Basidiospores. **e**. Pileus covering. Scale bars: 1 cm (**a**); 20 μm (**b, c**); 10 μm (**d**); 50 μm (**e**).

**Figure 36.**
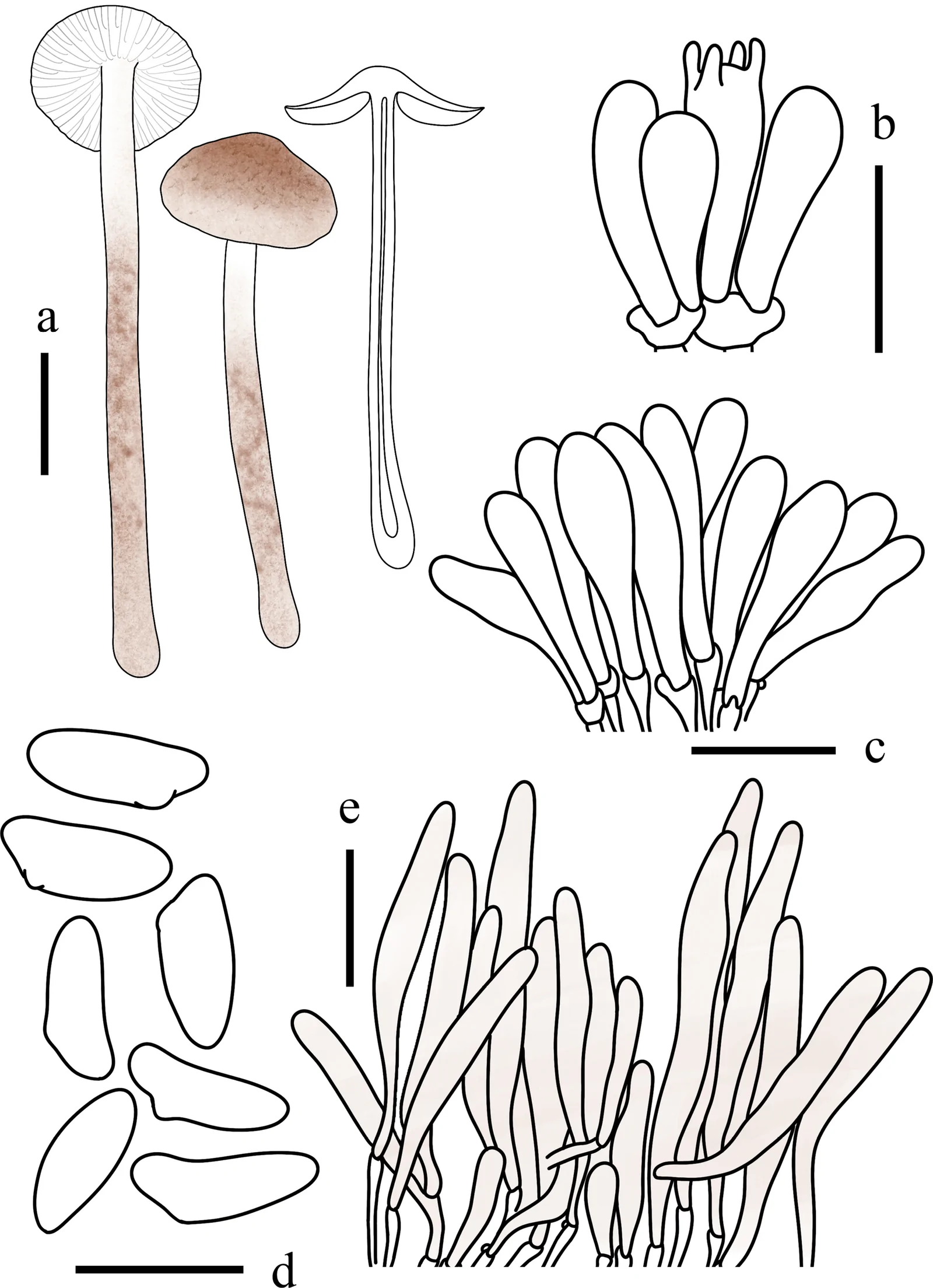
*Lepiota changbaiensis* (FJAU78297). **a**. Basidiomata. **b**. Basidia and basidioles. **c**. Cheilocystidia. **d**. Basidiospores. **e**. Pileus covering. Scale bars: 1 cm (**a**); 20 μm (**b, c**); 10 μm (**d**); 50 μm (**e**).

**Figure 37.**
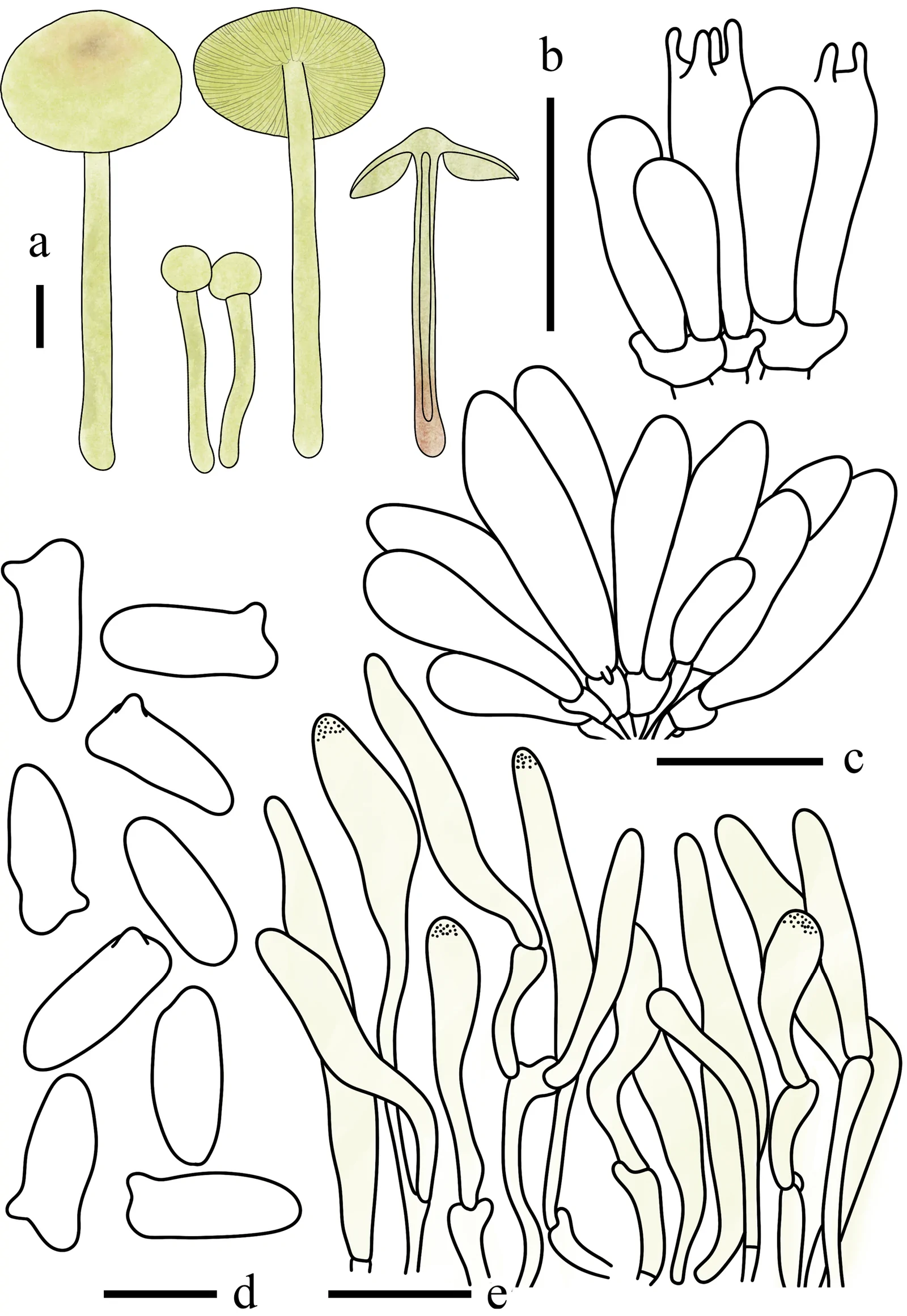
*Lepiota flavoviridis* (FJAU78219). **a**. Basidiomata. **b**. Basidia and basidioles. **c**. Cheilocystidia. **d**. Basidiospores. **e**. Pileus covering. Scale bars: 1 cm (**a**); 20 μm (**b, c**); 10 μm (**d**); 50 μm (**e**).

**Figure 38.**
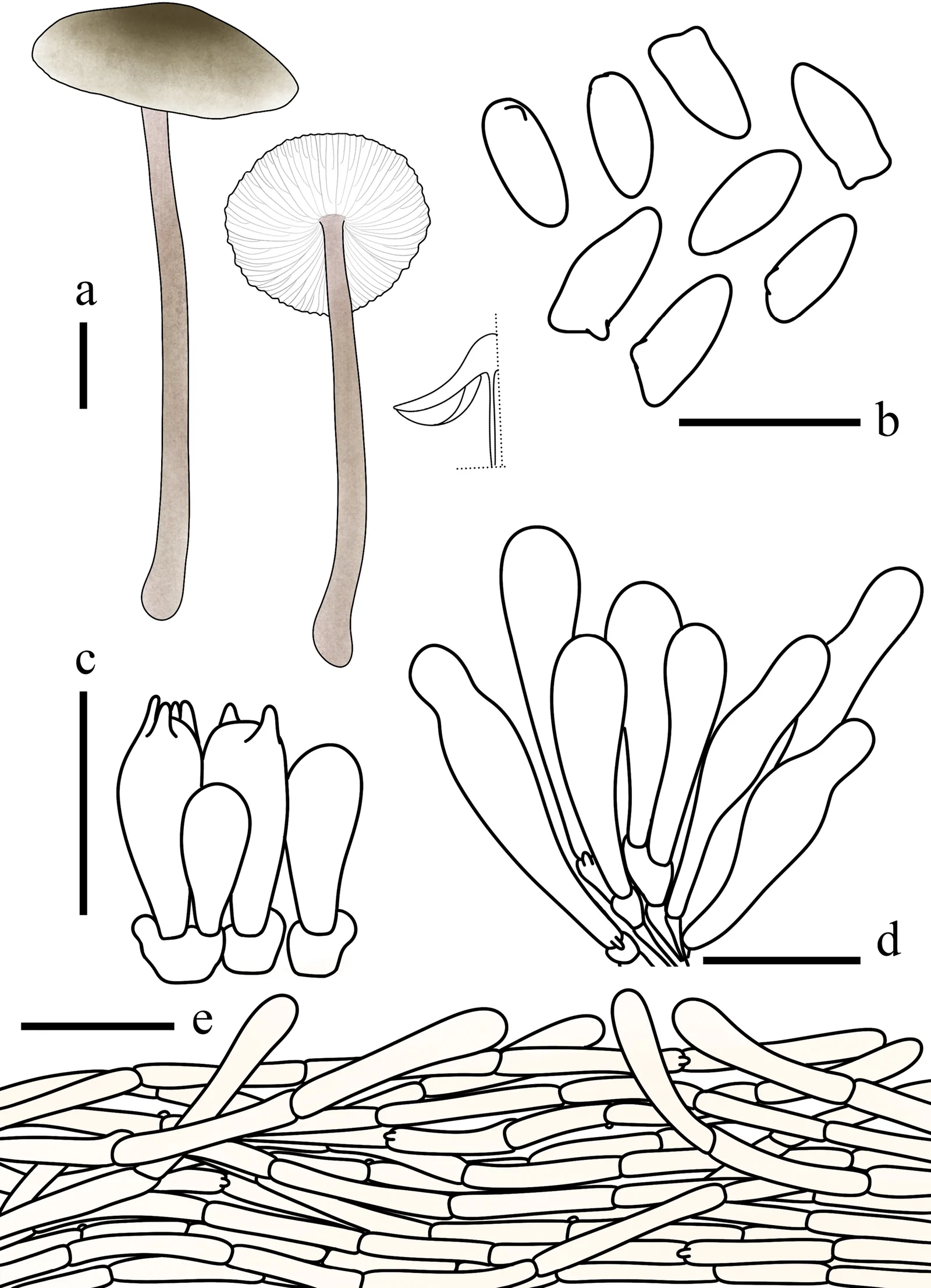
*Lepiota fulvoolivacea* (FJAU78250). **a**. Basidiomata. **b**. Basidiospores. **c**. Basidia and basidioles. **d**. Cheilocystidia. **e**. Pileus covering. Scale bars: 1 cm (**a**); 10 μm (**b**); 20 μm (**c, d**); 50 μm (**e**).

## Discussion

### Phylogenetic reassessment at the generic level

Based on a concatenated dataset of four gene regions (nrITS, nrLSU, *rpb2*, and *tef1-α*), we reconstructed the phylogenetic relationships of *Verrucosporaceae*. The resulting topologies align closely with the framework recently proposed by Li et al. (2025), confirming that *Verrucosporaceae* constitutes a well-supported monophyletic group. Within this framework, however, the generic boundaries of several core taxa warrant detailed re-evaluation.

Although the genus *Lepiota* is recovered as a monophyletic lineage, it received weak bootstrap support in our Maximum Likelihood (ML) analysis. This topological uncertainty is likely driven by several phylogenetically unstable or long-branched *incertae sedis* lineages within the genus, such as the *Lepiota angusticystidiata*, *L. brunneogranulosa*, *L. pyrochroa*, and *L. nigrescentipes* clades. These lineages occupied similarly unresolved positions in previous phylogenies (Li et al. 2025; Sarawi et al. 2025a).

At the base of *Verrucosporaceae*, *Cystolepiota* s.l. forms a strongly supported monophyletic clade (100/1) wherein *Melanophyllum* is deeply nested, which is consistent with recent studies (Qu et al. 2023; Zhou and Bau 2024; Li et al. 2025). Despite some macroscopic differences, these taxa share critical micro-morphological homologies, notably a pileus covering composed of loosely arranged sphaerocysts. Nomenclaturally, although *Melanophyllum* (1921) predates *Cystolepiota* (1952), the latter accommodates a significantly larger number of described species. To prevent extensive new combinations, we follow recent taxonomic treatments (Zhou and Bau 2024; Li et al. 2025) and continue to adopt the broad concept of *Cystolepiota* s.l. to preserve nomenclatural stability.

The genus *Verrucospora* was originally erected based on the African species *V. verrucispora* (Beeli) E. Horak (Horak 1967; Sysouphanthong et al. 2013). Recently, Li et al. (2025) demonstrated that its only other recognized member, *V. flavofusca* (Henn.) Jülich, is deeply nested within *Lepiota* sect. *Stenosporae* and formally recombined it as *L. flavofusca* (Henn.) R.L. Zhao & J.Xin Li. Our multi-locus analyses reveal that Asian collections of *V. verrucispora* also nest within this section, sharing a remarkably high nrITS sequence similarity (99.53%; Suppl. material 2) with *L. flavofusca*. These findings suggest that *Verrucospora* should be synonymized with *Lepiota*, and that the Asian *V. verrucispora* and *L. flavofusca* might represent a single species complex. However, because the African type specimen of *V. verrucispora* currently lacks molecular data, it remains uncertain whether the Asian collections represent the true African taxon or a distinct cryptic lineage. Adopting a conservative taxonomic approach, we refrain from formally synonymizing the genus or the two species, tentatively treating the Asian sequences as *Lepiota cf. verrucospora* pending the availability of sequence data from authentic African material to accurately resolve these boundaries.

### Infrageneric evolution and sectional delimitation in *Lepiota*

While the generic framework of *Verrucosporaceae* has gained stability, the sectional architecture of its most species-rich genus, *Lepiota*, remains complicated by morphological convergence. Our phylogenetic analyses help refine these sectional boundaries by resolving the placement of several morphologically ambiguous taxa.

In this study, we assessed *Cystolepiota icterina* and *C. squamulosa*, both historically placed near the base of *Cystolepiota* (Qu et al. 2023; Zhou and Bau 2024). Multi-locus analyses position *C. squamulosa* deeply within the core clade of *Lepiota* sect. *Eriophorae*, supporting the reinstatement of *Lepiota squamulosa*. Conversely, *C. icterina* clusters with *Lepiota boertmannii* and the newly described *L. fugaciosquamosa*. Together, they form an independent lineage sister to the core *L.* sect. *Eriophorae*. To accommodate this distinct clade, we propose a new subsection, *Lepiota* sect. *Eriophorae* subsect. *Pseudocystolepiotae*, and the new combination *Lepiota icterina*. This subsection represents a morphological and phylogenetic transition between *Cystolepiota* s.l. and core *Eriophorae*, combining *Cystolepiota*-like macroscopic traits with varying spore dextrinoidy.

Similar structural transitions are evident within *L.* sect. *Lepiota*. Clade B, diverging basally in this section, includes *L. efibulis*, *L. keralensis*, and *L. babruka*. Morphologically, the pileus covering of these basal taxa is either predominantly sphaerocystic or composed of a transitional cutis (Vellinga 2001b; Arun Kumar and Manimohan 2009; Pious and Arun 2025). This microstructure differs markedly from the typical trichoderm characteristic of its sister group, Clade A (Sarawi et al. 2025a). Such divergence suggests that Clade B may also act as an intermediate lineage bridging *Cystolepiota* and typical *Lepiota*, potentially explaining the moderate statistical support for *L.* sect. *Lepiota* as a whole.

Distinguishing the core *L.* sect. *Eriophorae* from *Echinoderma* has historically relied on basidiospore shape and the presence of cheilocystidia (Vellinga 2003; Hou and Ge 2020; Sarawi et al. 2025a). Mapping these diagnostic features onto our phylogeny (Fig. 3) supports earlier taxonomic observations: the basally diverging *Echinoderma* generally produces larger basidiomata and more slender spores (higher Q values), with distinct cheilocystidia. In contrast, terminal lineages within *L.* sect. *Eriophorae* largely lack cheilocystidia. Furthermore, scanning electron microscopy (SEM) confirms completely smooth basidiospore walls across all evaluated clades in these groups.

### Evolutionary and homological insights into pileus and stipe coverings

Pileus covering microstructure is a highly informative character in lepiotoid systematics (Vellinga 2003; Sarawi et al. 2025a). Knudsen (1978, 1980) demonstrated that the pileus covering in *L.* sect. *Echinatae* s.l. (encompassing current *L.* sect. *Eriophorae* and *Echinoderma*) differentiates into three cellular zones: basal hyphae, intermediate cells, and apical sphaerocysts. Cohesive chains of intermediate cells bind the squamules tightly. In contrast, *Cystolepiota* lacks this intermediate layer, causing the terminal sphaerocysts to readily detach and produce a characteristic mealy or powdery appearance.

Our observations support this three-layered framework for both *Echinoderma* and *L.* sect. *Eriophorae*, while identifying a notable divergence in the cellular composition of their intermediate layers. In *Echinoderma*, this layer consists of narrow, slender, septate hyphae. In *L.* sect. *Eriophorae*, it comprises broad, short-ellipsoid to long-cylindrical catenulate cells. This cellular differentiation provides a potentially diagnostic micro-character for separating these morphologically similar taxa. Although currently based on limited sampling—including the *Echinoderma asperum* complex, *E. petaloides*, and several *L.* sect. *Eriophorae* taxa (e.g., *L. granulosquarrosa*, *L. huadianensis*, *L. scabra*, *L. subhuadianensis*, *L. verruculosa*, *L. flavocarpa*, *L. omninoflava*, and *L. aff. jacobi* 1 and 2)—this structural pattern warrants further systematic validation.

Exceptions to this three-layered structure offer insights into pileus covering evolution. Despite nesting within the core clade of *L.* sect. *Eriophorae*, *Lepiota squamulosa* possesses a simplified two-layered pileus covering, structurally similar to *Cystolepiota* and *Melanophyllum*. However, its loosely arranged outermost sphaerocysts form persistent conical aggregations rather than a friable powder, indicating an independent structural reduction (Fig. 6).

A more complex structural transition is observed in the newly established *L.* subsect. *Pseudocystolepiotae*. While the basally diverging *L. boertmannii* retains the typical three-layered structure of core *Eriophorae*, its sister taxa *L. icterina* and *L. fugaciosquamosa* present a simplified two-layered structure. This microstructural variation suggests that *L.* subsect. *Pseudocystolepiotae* acts as an active structural transition state between the two-layered *Cystolepiota* s.l. and the three-layered core *L.* sect. *Eriophorae*.

Extending these observations reveals a strong structural homology between pileus and stipe coverings. On the stipes of most *Lepiota* and *Echinoderma* species, an initial fibrous basal layer is overlaid by punctate to granular scales. Microscopic examination shows that these granular stipe scales share the exact three-layered structure of the pileus covering (Fig. 7d–i). This correlation is supported by lineages exhibiting covering simplification: in Subclade D of *L.* sect. *Stenosporae*, although the pileus retains macroscopically small squamules, its covering is microscopically reduced to a cutis rather than the typical trichoderm. Correspondingly, the stipe covering directly mirrors this structural simplification, presenting entirely as a cutis devoid of trichodermal elements (Fig. 7a–c).

Consequently, these microstructural layers translate directly into macroscopic surface features. The basal micro-layer forms the macroscopic background tissue, the intermediate layer produces the surface fibrous or floccose elements, and the terminal sphaerocyst chains constitute the conical, verrucose, or granular squamules (Figs 4, 5). This macro-to-micro correlation provides a consistent morphological framework for tracing the structural evolution of basidioma coverings in lepiotoid fungi.

## Supplementary Material

XML Treatment for Cystolepiota

XML Treatment for Cystolepiota mastoidea

XML Treatment for Echinoderma

XML Treatment for Lepiota

XML Treatment for Leucocoprinus booloola

XML Treatment for Leucocoprinus gomezii

XML Treatment for Lepiota

XML Treatment for Lepiota cristatosimilis

XML Treatment for Lepiota

XML Treatment for Lepiota squamulosa

XML Treatment for Lepiota granulosquama

XML Treatment for Lepiota huadianensis

XML Treatment for Lepiota scabra

XML Treatment for Lepiota subhuadianensis

XML Treatment for Lepiota verruculosa

XML Treatment for Lepiota

XML Treatment for Lepiota icterina

XML Treatment for Lepiota fugaciosquamosa

XML Treatment for Lepiota

XML Treatment for Lepiota spinulosa

XML Treatment for Lepiota

XML Treatment for Lepiota basozonata

XML Treatment for Lepiota jianensis

XML Treatment for Lepiota rostratospora

XML Treatment for Lepiota subforquignonii

XML Treatment for Lepiota

XML Treatment for Lepiota brunneotacta

XML Treatment for Lepiota changbaiensis

XML Treatment for Lepiota flavoviridis

XML Treatment for Lepiota fulvoolivacea

XML Treatment for Melanophyllum

XML Treatment for Melanophyllum haematospermum

## Additional information

### Artificial Intelligence (AI) use

The authors accept full responsibility for the content of the manuscript, including the disclosure of any use of AI.

No AI tools were used in the preparation of this manuscript.

### Adherence to national and international regulations

All the fungal strains used in this study have been legally obtained, respecting the Convention on Biological Diversity (Rio Convention).

### Author ORCIDs

X.-Y. Zhou https://orcid.org/0009-0006-7119-8386

T. Bau https://orcid.org/0000-0003-2461-9345

### Data availability

The data that support the findings of this study are openly available in the National Center for Biotechnology Information (NCBI) GenBank database and the Index Fungorum database. All data generated or analyzed during this study are included in this published article and its Suppl. materials 1, 2.

## Supplementary materials

Supplementary material 1 Supplementary file 1 Data type zip https://binary.pensoft.net/file/1805418This dataset is made available under the Open Database License (http://opendatacommons.org/licenses/odbl/1.0/). The Open Database License (ODbL) is a license agreement intended to allow users to freely share, modify, and use this Dataset while maintaining this same freedom for others, provided that the original source and author(s) are credited. Xian-Yan Zhou, Tolgor Bau

Supplementary material 2 Supplementary file 2 Data type zip https://binary.pensoft.net/file/1805419This dataset is made available under the Open Database License (http://opendatacommons.org/licenses/odbl/1.0/). The Open Database License (ODbL) is a license agreement intended to allow users to freely share, modify, and use this Dataset while maintaining this same freedom for others, provided that the original source and author(s) are credited. Xian-Yan Zhou, Tolgor Bau
